# Supplementary material for: Impact of immunosuppressive therapy on SARS-CoV-2 mRNA vaccine effectiveness in patients with immune-mediated inflammatory diseases: a Danish nationwide cohort study
Source: BMJ Open. 2024 Feb 21;14(2):e077408. doi: 10.1136/bmjopen-2023-077408 (PMC10882296; doi:10.1136/bmjopen-2023-077408)

The Impact of Immunosuppressive Therapy on SARS-CoV-2 mRNA Vaccine Effectiveness in Patients with Immune-mediated Inflammatory Diseases: A Danish Nationwide Cohort Study

Rahma Elmahdi[*1,2] (0000-0002-7013-9548), Daniel Ward[*1] (0000-0003-0405-7250), Martin Thomsen Ernst[3] (0000-0001-9003-3823), Gry Juul Poulsen[1] (0000-0003-2744-2469), Jesper Hallas[3] (0000-0002-8097-8708), Anton Pottegård[3] (0000-0001-9314-5679), Tine Jess[1,4] (0000-0002-4391-7332)

SUPPLEMENTARY TABLES

Supplementary Table 1. Exposures and corresponding ATC and procedure codes for immunosuppressives

Medication	ATC code	Procedure code
**Selective immunosuppressants**		
Muromonab-CD3	L04AA02	
Antilymphocyte immunoglobulin	L04AA03	
(horse)	L04AA04	BOHJ12
Antithymocyte immunoglobulin (rabbit)	L04AA06	BOHJ22
Mycophenolic acid	L04AA10	BOHJ23
Sirolimus	L04AA13	
Leflunomide	L04AA18	BOHJ24
Everolimus	L04AA23	BOHJ26
Natalizumab	L04AA24	BOHJ18C1
Abatacept	L04AA25	BWHB84
Eculizumab	L04AA26	BOHJ19H6
Belimumab	L04AA27	BOHJ27
Fingolimod	L04AA28	
Belatacept	L04AA29	BOHJ28D
Tofacitinib	L04AA31	BOHJ28A
Teriflunomide	L04AA32	
Aprelimast	L04AA33	BOHJ19H4
Vedolizumab	L04AA34	BOHJ16A
Alemtuzumab	L04AA36	
Ocrelizumab	L04AA37	
Baricitinib	L04AA38	
Ozanimod	L04AA39	
Emapalumab	L04AA40	BWHA178
Cladribine	L04AA41	
Imlifidase	L04AA42	BWHB87
Siponimod	L04AA43	
Ravulizumab	L04AA44	
Upadacitinib		

**Tumour necrosis factor inhibitors**		
Etanercept	L04AB01	BOHJ18A2
Infliximab	L04AB02	BOHJ18A1
Adalimumab	L04AB04	BOHJ18A3
Certolizumab pegol	L04AB05	BOHJ18A5
Golimumab	L04AB06	BOHJ18A4
**Interleukin inhibitors**		
Basiliximab	L04AC02	
Anakinra	L04AC03	BOHJ18B1
Ustekinumab	L04AC05	BOHJ18B3
Tocilizumab	L04AC07	BOHJ18B2
Canakinumab	L04AC08	BOHJ18B4
Secukinumab	L04AC10	BOHJ18B5
Siltuximab	L04AC11	
Brodalumab	L04AC12	BOHJ18B6
Ixekizumab	L04AC13	
Sarilumab	L04AC14	BOHJ18B9
Guselkumab	L04AC16	
Tildrakizumab	L04AC17	BOHJ18B7
Risankizumab	L04AC18	BOHJ19N1
**Calcineurin inhibitors**		
Ciclosporin	L04AD01	BOHJ20
Tacrolimus	L04AD02	BOHJ21
**Other immunosuppressants**		
Azathioprine	L04AX01	BWHB83
Thalidomide	L04AX02	BWHB81
Methotrexate	L04AX03	BWHA115
Lenalidomide	L04AX04	BWHB82
Pirfenidone	L04AX05	BWHB85
Pomalidomide	L04AX06	BWHB86
Dimethyl fumarate	L04AX07	BOHJ28B
Darvadstrocel	L04AX08	
**Systemic corticosteroids**	H02AB	
**Rituximab**	L01XC02	

Supplementary Table 2. Minimum daily dose of corticosteroid in exposed persons

Compound	Equivalent dose
Cortisone	38mg
Hydrocortisone	30mg
Prednisone	7.5mg
Prednisolone	7.5mg
Triamcinolone	6mg
Methylprednisolone	6mg
Betamethasone	0.9mg
Dexamethasone	1.2mg

Supplementary Table 3. Covariates by disease cohort

IBD COHORT		
**Covariate**	**Categories/ ATC/ICD codes**	**Assessment window**
Age	-	At cohort entry
Sex	-	At cohort entry
SARS-CoV-2 tests in the previous month	-	At cohort entry
Ever previous SARS-CoV-2 positive test	-	From February 2020
IBD specific covariates		
Any IBD–related hospital admissions in the previous year	K50/K51	1 year before cohort entry
Crohn's disease	K50	At cohort entry
Ulcerative colitis	K51	At cohort entry
5-ASA/sulfasalazine	MA07EC0	1 year before matching date
Budesonide	MA07EA06	3 months before matching date
IBD related procedures<br>Operations of the small bowel and colon<br>Operations of the rectum<br>Operations of the anus and perianal tissue<br>Operational of the abdominal wall, peritoneum, mesentery and omentum<br>Lysis of adhesion in the abdominal cavity<br>Closure of intestinovaginal fistula<br>Closure of vesiculointestinal fisutal	 KJF<br>KJG<br>KJH<br>KJA<br>KJAP<br>KLEE30<br>KKCH30	Any time before cohort entry
Endoscopy of the gastrointestinal tract	KUJ	1 year before the cohort entry
Comorbidities		
Cardiovascular disease	DI1-I7	Any time before cohort entry
Pulmonary disease<br>Chronic disease of the lower airways<br>Other interstitial lung disease<br>Diseases with pus and necrosis in the lower airway<br>Interstitial lung emphysema<br>compensatory emphysema	 DJ4<br>DJ84<br>DJ85<br>DJ982<br>DJ983	Any time before cohort entry
Liver disease	DK70-K77	Any time before cohort entry
Kidney disease<br>Glomerular disease<br>Tubulointerstitial kidney disease and kidney insufficiency	 DN0<br>DN1	Any time before cohort entry
Other gastrointestinal diseases<br>Gingivitis and periodontal disease<br>Inflammation of the oral mucosa and related<br>Stomach and duodenal ulcers<br>Fissure and rifts in and around the anus<br>Abscess in and around the anus<br>Other diseases of the rectum and anus<br>Other bowel disease	 DK05<br>DK12<br>DK25-27<br>DK60<br>DK61<br>DK62<br>DK63	Any time before cohort entry

Skin disease<br>Bullous skin disease<br>Dermatitis and eczema<br>Alopecia areata<br>Vitiligo<br>Granulomatous disease in the skin and subcutaneous tissue<br>Lupus<br>Other localised connective tissue disease<br>Vasculitis limited to the skin	 DL10-14<br>DL20-30<br>DL63<br>DL80<br>DL92<br>DL93<br>DL94<br>DL95	Any time before cohort entry
Musculoskeletal disease<br>Generalised connective tissue diseases<br>Diseases of the muscles<br>Soft-tissue rheumatism	 DM30-<br>DM36<br>DM60-63<br>DM70-79	Any time before cohort entry
Medications		
Cardiovascular drugs	MC01-MC10	Any time before cohort entry
Antibiotics	MJ01	Any time before cohort entry
Oral anticoagulants	MB01AA<br>MB01AF	Any time before cohort entry
Drugs used in diabetes	MA10	Any time before cohort entry
Drugs for obstructive airway diseases	MR03	Any time before cohort entry
INFLAMMATORY ARTHROPATHIES COHORT		
Age	-	At cohort entry
Sex	-	At cohort entry
SARS-CoV-2 tests in the previous month	-	At cohort entry
Arthropathy-specific covariates		
Arthropathy-related procedures<br>Shoulder and upper arm<br>Primary insertion of joint prosthesis<br>Secondary insertion of joint prosthesis<br>Operations on the joint capsule and ligaments<br>Operations of the synovia and joint surface<br>Joint resections, arthroplasties, and arthrodesis<br>Excision of bursa<br>Elbow and lower arm<br>Primary insertion of joint prosthesis<br>Secondary insertion of joint prosthesis<br>Operations on the joint capsule and ligaments<br>Operations of the synovia and joint surface<br>Joint resections, arthroplasties, and arthrodesis<br>Excision of bursa<br>Wrist and hand<br>Primary insertion of joint prosthesis<br>Secondary insertion of joint prosthesis<br>Operations on the joint capsule and ligaments<br>Operations of the synovia and joint surface<br>Joint resections, arthroplasties, and arthrodesis<br>Operations on the fascia, tendon sheaths, ganglia and bursae	 <br>KNBB<br>KNBC<br>KNBE<br>KNBF<br>KNBG<br>KBM79<br><br>KNCB<br>KNCC<br>KNCE<br>KNCF<br>KNCG<br>KNCM79<br><br>KNDB<br>KNDC<br>KNDE<br>KNDF<br>KNDG<br>KNDM	Any time before cohort entry

Hip and thigh		
Primary insertion of joint prosthesis	KNFB	
Secondary insertion of joint prosthesis	KNFC	
Operations on the joint capsule and ligaments	KNFE	
Operations of the synovia and joint surface	KNFF	
Joint resections, arthroplasties, and arthrodesis	KNFG	
Excision of bursa in the	KNFM79	
Knee and lower leg		
Primary insertion of joint prosthesis	KNGB	
Secondary insertion of joint prosthesis	KNGC	
Operations on the joint capsule and ligaments	KNGE	
Operations of the synovia and joint surface	KNGF	
Joint resections, arthroplasties, and arthrodesis	KNGG	
Excision of bursae	KNGM79	
Ankle and foot		
Primary insertion of joint prosthesis	KNHB	
Secondary insertion of joint prosthesis	KNHC	
Operations on the joint capsule and ligaments	KNHE	
Operations of the synovia and joint surface	KNHF	
Joint resections, arthroplasties, and arthrodesis	KNHG	
Operations on the fascia, tendon sheaths, ganglia and bursae	KNHM	
Anti-inflammatory and anti-rheumatic drugs		3 months before before cohort entry
inc. specific anti-rheumatic therapies, non-steroidals, and combination medications	M01	
Hydroxychloroquine	P01BA02	
Comorbidities		
Cardiovascular disease	DI1-I7	Any time before cohort entry
Pulmonary disease		Any time before cohort entry
Chronic disease of the lower airways	DJ4	
Other interstitial lung disease	DJ84	
Diseases with pus and necrosis in the lower airway	DJ85	
Interstitial lung emphysema	DJ982	
compensatory emphysema	DJ983	
Liver disease	DK70-K77	Any time before cohort entry
Kidney disease		Any time before cohort entry
Glomerular disease	DN0	
Tubulointerstitial kidney disease and kidney insufficiency	DN1	
Other gastrointestinal diseases		Any time before cohort entry
Gingivitis and periodontal disease	DK05	
Inflammation of the oral mucosa and related	DK12	
Stomach and duodenal ulcers	DK25-27	
Fissure and rifts in and around the anus	DK60	
Abscess in and around the anus	DK61	
Other diseases of the rectum and anus	DK62	
Other bowel disease	DK63	
Skin disease		Any time before cohort entry
Bullous skin disease	DL10-14	
Dermatitis and eczema	DL20-30	
Alopecia areata	DL63	

Vitiligo<br>Granulomatous disease in the skin and subcutaneous tissue<br>Lupus<br>Other localised connective tissue disease<br>Vasculitis limited to the skin	DL80<br>DL92<br>DL93<br>DL94<br>DL95	
Musculoskeletal disease<br>Generalised connective tissue diseases<br>Diseases of the muscles<br>Soft-tissue rheumatism	DM30-<br>DM36<br>DM60-63<br>DM70-79	Any time before cohort entry
Medications		
Cardiovascular drugs	MC01-MC10	Any time before cohort entry
Antibiotics	MJ01	Any time before cohort entry
Oral anticoagulants	MB01AA<br>MB01AF	Any time before cohort entry
Drugs used in diabetes	MA10	Any time before cohort entry
Drugs for obstructive airway diseases	MR03	Any time before cohort entry
PSORIASIS COHORT		
Age	-	At cohort entry
Sex	-	At cohort entry
SARS-CoV-2 tests in the previous month	-	At cohort entry
Psoriasis-specific covariates		
Procedures<br>Ultraviolet therapy: UV-A with psoralen/broad-spectrum UVB / narrow-spectrum UVB	BNGA1/<br>BNGA2/<br>BNGA3	Any time before cohort entry
Therapeutic steroid injection in the joints and soft tissues	BLHN0	Any time before cohort entry
5-ASA/sulfasalazine	A07EC0	1 year before cohort entry
Topical corticosteroids	D07	1 year before cohort entry
Antipsoriatic medication	D05	1 year before cohort entry
Topical calcineurin inhibitors	D11AH01-02,	1 year before cohort entry
Comorbidities		
Cardiovascular disease	DI1-I7	Any time before cohort entry
Pulmonary disease<br>Chronic disease of the lower airways<br>Other interstitial lung disease<br>Diseases with pus and necrosis in the lower airway<br>Interstitial lung emphysema<br>compensatory emphysema	DJ4<br>DJ84<br>DJ85<br>DJ982<br>DJ983	Any time before cohort entry

Liver disease	DK70-K77	Any time before cohort entry
Kidney disease<br>Glomerular disease<br>Tubulointerstitial kidney disease and kidney insufficiency	 DN0<br>DN1	Any time before cohort entry
Other gastrointestinal diseases<br>Gingivitis and periodontal disease<br>Inflammation of the oral mucosa and related<br>Stomach and duodenal ulcers<br>Fissure and rifts in and around the anus<br>Abscess in and around the anus<br>Other diseases of the rectum and anus<br>Other bowel disease	 DK05<br>DK12<br>DK25-27<br>DK60<br>DK61<br>DK62<br>DK63	Any time before cohort entry
Skin disease<br>Bullous skin disease<br>Dermatitis and eczema<br>Alopecia areata<br>Vitiligo<br>Granulomatous disease in the skin and subcutaneous tissue<br>Lupus<br>Other localised connective tissue disease<br>Vasculitis limited to the skin	 DL10-14<br>DL20-30<br>DL63<br>DL80<br>DL92<br>DL93<br>DL94<br>DL95	Any time before cohort entry
Musculoskeletal disease<br>Generalised connective tissue diseases<br>Diseases of the muscles<br>Soft-tissue rheumatism	 DM30-<br>DM36<br>DM60-63<br>DM70-79	Any time before cohort entry
Medications		
Cardiovascular drugs	MC01-MC10	Any time before cohort entry
Antibiotics	MJ01	Any time before cohort entry
Oral anticoagulants	MB01AA<br>MB01AF	Any time before cohort entry
Drugs used in diabetes	MA10	Any time before cohort entry
Drugs for obstructive airway diseases	MR03	Any time before cohort entry

Supplementary Table 4. Risk of infection, hospitalisation and death associated with immunosuppressive therapy in vaccinated patients with immune-mediated inflammatory diseases.

	**Exposed**				**Unexposed**				**Crude HR (95% CI) *****	**Weighted HR (95% CI) *****
	**Total**	**Events**	**Incidence rate (95% CI) ****	**PY**	**Total**	**Events**	**Incidence rate (95% CI) ****	**PY**		
**COVID-19 Infection***										
IBD	10 477	287	54.7 (48.7-61.4)	5 246	46 974	885	42.5 (39.8-45.4)	20 809	1.7 (1.5-1.9)	1.6 (1.4-1.9)
Arthropathy	24 255	476	40.3 (36.8-44.1)	11 822	44 650	800	37.8 (35.3-40.6)	21 140	1.3 (1.1-1.4)	1.3 (1.1-1.4)
Psoriasis	5 023	103	45.3 (37.4-55.00)	2 273	20 995	392	41.5 (37.6-45.8)	9 441	1.1 (0.9-1.4)	1.1 (0.9-1.4)
Combined cohorts	39 755	866	-	19 341	112 619	2 077	-	51 390	1.4 (1.3-1.5)	1.4 (1.2-1.5)
**COVID-19 Hospitalization***										
IBD	10 477	15	2.8 (1.7-4.7)	5 280	46 980	43	2.1 (1.5-2.8)	20 892	2.8 (1.5-5.1)	2.1 (1.0-4.1)
Arthropathy	24 256	37	3.1 (2.3-4.3)	11 873	44 650	59	2.8 (2.2-3.6)	21 215	1.3 (0.9-2.0)	1.3 (0.9-2.0)
Psoriasis	5 023	<5	-	2 284	20 999	20	2.1 (1.4-3.3)	9 479	0.5 (0.1-2.3)	0.6 (0.1-2.5)
Combined cohorts	39 756	<57	-	19 437	112 629	122	-	51 594	1.6	1.4

									(1.1-2.2)	(1.0-2.0)
**Death***										
IBD	10 477	<5	-	5 281	46 980	6	0.3 (0.1-0.6)	20 897	1.1 (0.1-9.7)	0.4 (0-5.6)
Arthropathy	24 256	6	0.51 (0.2-1.1)	11 875	44 650	13	0.6 (0.4-1.1)	21 222	1.0 (0.4-2.7)	1.0 (0.4-2.6)
Psoriasis	5 023	0	-	2 284	20 999	<5	-	9 480	-	-
Combined cohorts	39 756	<11	-	19 440	112 629	<24	-	51 599	1.0 (0.4-2.5)	0.9 (0.4-2.2)

Abbreviations: CI = confidence interval; HR = hazard ratio; IBD= inflammatory bowel disease; PY = person-years.

*COVID19- Infection = confirmed positive antigen or PCR test result for SARS-CoV2; COVID-19 Hospital Admission = Hospitalisation with SARS-CoV2 infection as primary diagnosis code; Death within 60 days of positive SARS-CoV2 test.

**Events/1000 PY (95% CI)

*** Sex and age adjusted crude and weighted hazard ratios

Supplementary Table 5. Risk of COVID-19 infection associated with immunosuppressive therapy in vaccinated patients with immune-mediated inflammatory diseases at 0-3 months, 3-6 months, and 6-11 months post-vaccination.

	**Exposed**				**Unexposed**				**Crude HR (95% CI)** **	**Weighted HR (95% CI)** **
	**Total**	**Events**	**Incidence rate (95% CI)** *	**PY**	**Total**	**Events**	**Incidence rate (95% CI)** *	**PY**		
**COVID-19 infection 0-3 months post-vaccination**										
IBD	10 477	57	22.2 (17.1-28.8)	2 570	46 974	196	17.0 (14.8-19.5)	11 546	1.6 (1.2-2.2)	1.5 (1.1-2.2)
Arthropathy	24 255	96	16.1 (13.2-19.6)	5 978	44 650	166	15.1 (13.0-17.6)	10 990	1.3 (1.0-1.7)	1.3 (1.0-1.7)
Psoriasis	5 023	24	19.4 (13.0-29.0)	1 236	20 995	83	16.1 (13.0-19.9)	5 163	1.3 (0.3-2.1)	1.3 (0.8-2.1)
Combined cohorts	39 755	177	-	4 403	112 619	445	-	27 699	1.4 (1.2-1.7)	1.4 (1.1-1.6)
**COVID-19 infection 3-6 months post-vaccination**										
IBD	10 010	123	45.8 (38.4-54.7)	2 683	45 202	526	56.8 (52.1-61.8)	9 266	0.9 (0.8-1.2)	1.0 (0.8-1.2)
Arthropathy	23 503	284	48.6 (43.2-54.5)	5 849	43 186	482	47.5 (43.4-51.9)	10 149	1.2 (1.1-1.4)	1.2 (1.1-1.4)
Psoriasis	4 860	63	60.7 (47.4-77.7)	1 038	20 203	233	54.5 (47.9-61.9)	4 279	1.1 (0.8-1.4)	1.0 (0.8-1.4)
Combined cohorts	38 373	470	-	19 437	108 591	1 241	-	23 694	1.1 (1.0-1.2)	1.1 (1.0-1.3)

**COVID-19 infection 6-11 months post-vaccination**										
IBD	6 058	107	160.6 (132.9-194.1)	666	15 742	163	107.1 (91.9-124.9)	1 522	1.3 (0.9-1.7)	1.4 (1.1-1.9)
Arthropathy	12 223	96	82.7 (67.7-101.0)	1 160	19 839	152	80.8 (68.9-94.7)	1 881	1.1 (0.8-1.4)	1.1 (0.8-1.4)
Psoriasis	1 719	16	93.8 (57.5-153.1)	170	7 444	76	109.8 (87.7-137.5)	691	0.9 (0.5-1.5)	0.9 (0.5-1.5)
Combined cohorts	20 000	219	-	1 996	43 025	391	-	4 094	1.1 (0.9-1.3)	1.11 (0.9-1.3)

Abbreviations: CI = confidence interval; HR = hazard ratio; IBD= inflammatory bowel disease; PY = person-years.

*Events/1000 PY

**Sex and age adjusted crude and weighted hazard ratios

Supplementary Table 6. Risk of COVID infection associated with immunosuppressive therapy in vaccinated patients with immune-mediated inflammatory diseases by calendar period (1st January-31st November 2021)

	Exposed		Unexposed		Hazard Ratio (HR)	
	**Total**	**Events**	**Total**	**Events**	**Crude HR (95% CI)**	**Weighted HR (95% CI)** **
***COVID-19 infection from 1st January to 30th April**						
IBD	5 745	n<5	10 306	7	0.67 (0.17-2.61)	0.39 (0.09-1.71)
Arthropathy	9 646	9	13 044	17	0.75 (0.33-1.69)	0.78 (0.35-1.71)
Psoriasis	1 298	n<5	4 512	6	2.00 (0.50-8.02)	1.98 (0.48-8.19)
Combined IMID	16 689	<19	27 862	30	0.87 (0.48-1.59)	0.83 (0.44-1.54)
**COVID-19 infection 1st May to 31st August**						
IBD	10 141	56	45 486	121	1.61 (1.17-2.22)	1.19 (0.82-1.73)
Arthropathy	23 885	74	43 457	116	1.08 (0.81-1.45)	1.07 (0.79-1.43)
Psoriasis	4 912	12	20 337	53	0.88 (0.47-1.65)	0.84 (0.44-1.58)
Combined IMID	38 938	142	109 280	290	1.24 (1.01-1.52)	1.08 (0.87-1.34)
**COVID-19 infection 1st September to 30st November**						
IBD	1 0349	228	46 590	757	1.69 (1.45-1.97)	1.74 (1.48-2.04)
Arthropathy	23 898	393	44 079	667	1.34 (1.18-1.51)	1.33 (1.17-1.51)
Psoriasis	4 974	88	20 803	333	1.17 (0.93-1.48)	1.13 (0.89-1.44)
Combined IMID	39 221	709	111 472	1757	1.42 (1.30-1.55)	1.42 (1.29-1.56)

Abbreviations: CI = confidence interval; HR = hazard ratio; IBD= inflammatory bowel disease.

*COVID19- Infection = confirmed positive antigen or PCR test result for SARS-CoV2

** Sex and age adjusted crude and weighted hazard ratios

Supplementary Table 7. Risk of infection, hospitalisation and death associated with immunosuppressive therapy in vaccinated patients with immune-mediated inflammatory diseases by immunosuppressive drug class.

	**Exposed**				**Unexposed**				**Crude HR (95% CI)** ***	**Weighted HR (95% CI)** ***
	**Total**	**Events**	**PY**	**Incidence rate (95% CI)** **	**Total**	**Events**	**PY**	**Incidence rate (95% CI)** **		
**COVID-19 Infection***										
Selective Immunosuppressants	3 384	68	1 846	39.4 (31.0-49.9)	151 936	2 079	51 334	40.5 (38.8-42.3)	1.3 (1.0-1.7)	1.3 (1.0-1.6)
Tumour Necrosis Factor Inhibitors	14 277	418	6 908	62.5 (56.7-68.7)	140 797	2 070	51 355	40.3 (38.6-42.1)	1.8 (1.6-2.0)	1.8 (1.6-2.0)
Interleukin Inhibitors	2 781	52	1 385	40.1 (30.6-52.7)	152 584	2 082	51 352	40.5 (38.8-42.3)	1.1 (0.8-1.4)	1.0 (0.8-1.4)
Calcineurin Inhibitors	154	n<5	129	-	155 309	2 073	51 298	40.4 (38.7-42.2)	1.3 (0.3-5.2)	0.5 (0.2-1.5)
Systemic Corticosteroids	5 504	97	2 991	34.6 (28.4-42.3)	149 667	2 079	51 503	40.4 (38.7-42.1)	1.3 (1.0-1.6)	1.2 (1.0-1.5)
Rituximab and Other Immunosuppressants	21 038	426	10 703	41.2 (37.5-45.3)	112 593	2 075	51 409	40.4 (38.7-42.1)	1.3 (1.2-1.4)	1.3 (1.1-1.4)
**COVID-19 Hospitalization***										
Selective Immunosuppressants	3 384	7	1 735	4.0 (1.9-8.5)	112 479	122	51 531	2.4 (2.0-2.8)	3.0 (1.4-6.5)	2.2 (1.0-5.2)
Tumour Necrosis Factor Inhibitors	14 277	16	6 740	2.4 (1.5-3.9)	112 506	116	51 551	2.3 (1.9-2.1)	2.0 (1.2-3.3)	1.8 (1.0-3.3)

Interleukin Inhibitors	2 781	0	1 302	-	112 445	125	51 549	2.4 (2.0-2.9)	-	-
Calcineurin Inhibitors	154	0	83	-	112 404	122	51 493	2.4 (2.0-2.8)	-	-
Systemic Corticosteroids	5 504	18	2 811	6.4 (4.0-10.2)	112 587	122	51 699	2.4 (2.0-2.8)	2.4 (1.4-3.9)	1.8 (1.0-3.0)
Rituximab and Other Immunosuppressants	21 038	25	10 387	2.4 (1.6-3.6)	112 593	121	51 604	2.3 (2.0-2.8)	1.2 (0.5-1.0)	1.1 (0.7-1.8)
**Death***										
Selective Immunosuppressants	3 384	0	1 736	-	112479	23	51 545	0.5 (0.3-0.7)	-	-
Tumour Necrosis Factor Inhibitors	14 277	0	6 741	-	112506	19	5 1564	0.4 (0.2-0.6)	-	-
Interleukin Inhibitors	2 781	0	1 302	-	112 445	24	51 564	0.5 (0.3-0.7)	-	-
Calcineurin Inhibitors	154	0	83	-	112 404	24	51 507	0.5 (0.3-0.7)	-	-
Systemic Corticosteroids	5 504	6	2 812	2.1 (2.0-4.8)	112 587	23	51 713	0.4 (0.3-0.7)	4.0 (1.6-9.9)	2.3 (0.9-6.2)
Rituximab and Other Immunosuppressants	21 038	n<5	10 388	-	112 593	22	51 619	0.4 (0.3-0.7)	0.5 (0.1-2.3)	0.5 (0.1-2.4)

Abbreviations: CI = confidence interval; HR = hazard ratio; IBD= inflammatory bowel disease; PY = person-years.

Immunosuppressive Drug Class: Selective Immunosuppressants, Tumour Necrosis Factor Inhibitors, Interleukin Inhibitors, Calcineurin Inhibitors, Systemic Corticosteroids, and Other Immunosuppressants including Rituximab (See Supplementary for complete ATC codes)

[*]COVID19- Infection = confirmed positive antigen or PCR test result for SARS-CoV2; COVID-19 Hospital Admission = Hospitalisation with SARS-CoV2 infection as primary diagnosis code; Death within 60 days of positive SARS-CoV2 test.

[**]Events/1000 PY

[***] Sex and age adjusted crude and weighted hazard ratios

SUPPLEMNETRAY FIGURES

Supplementary Figure 1. Kaplan-Meier plots for probability of infection with COVID-19 following second mRNA vaccination in immunosuppressive exposed compared to propensity score matched immunosuppressive unexposed a. IBD patients b. Arthropathy patients c. Psoriasis patients over the calendar year from January 1st to 31st November 2021. SMR weighted = standardized mortality ratio weighted.

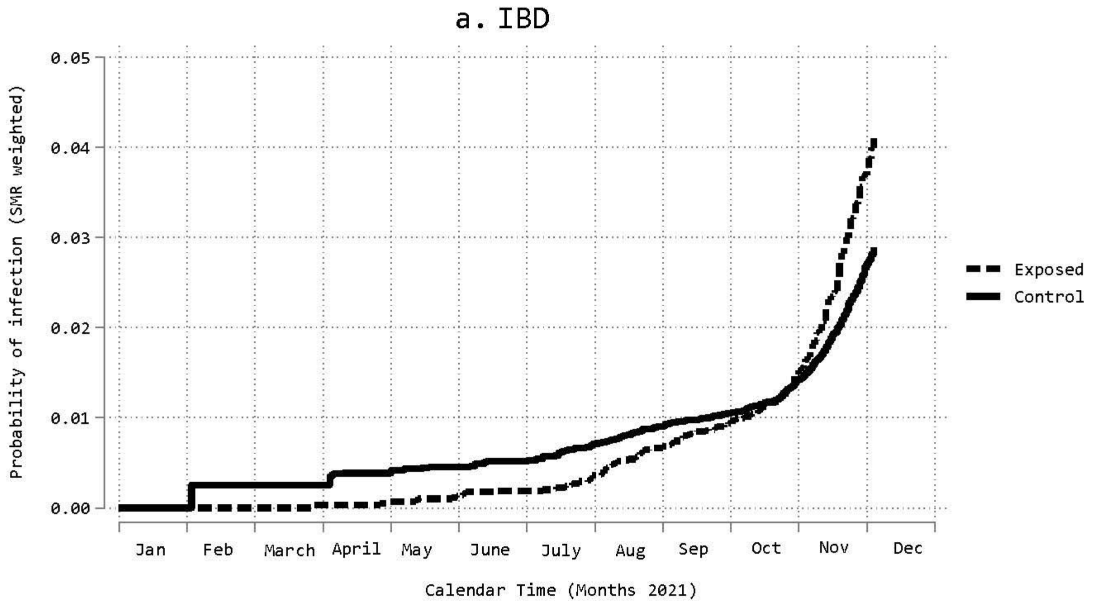

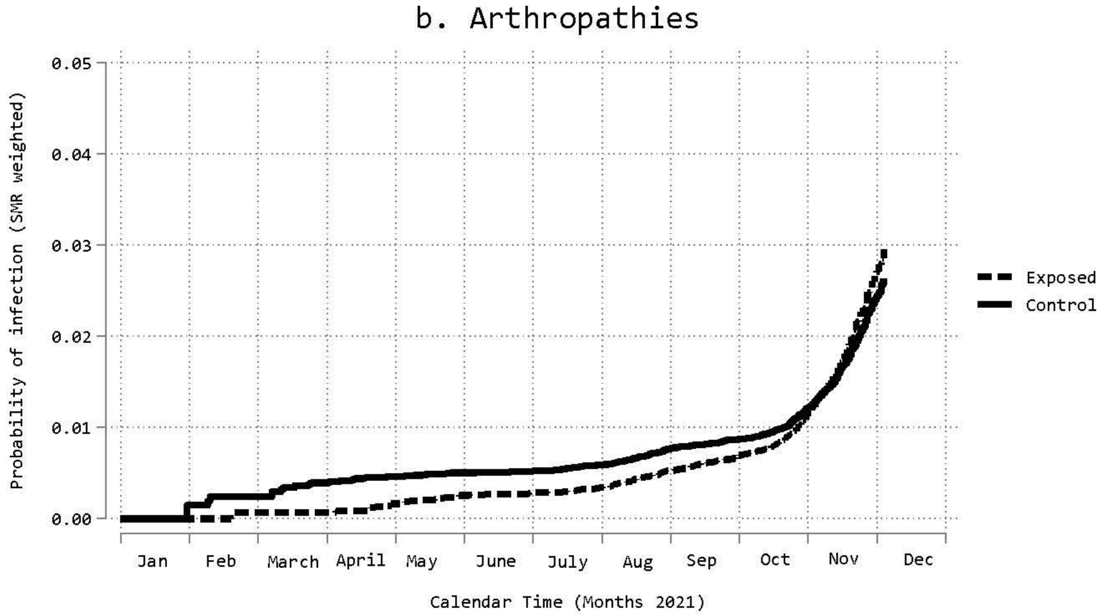

BMJ Publishing Group Limited (BMJ) disclaims all liability and responsibility arising from any reliance placed on this supplemental material which has been supplied by the author(s)

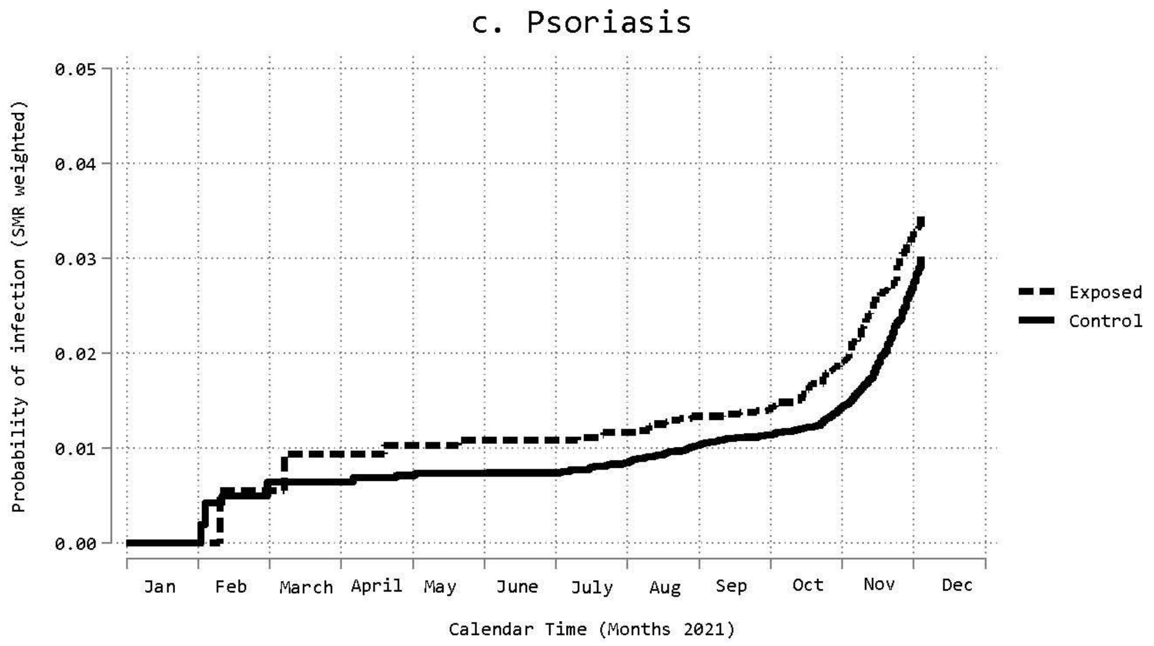

Supplement: Supplementary data [file bmjopen-2023-077408supp001.pdf]
