# Supplementary material for: Impact of immunosuppressive therapy on SARS-CoV-2 mRNA vaccine effectiveness in patients with immune-mediated inflammatory diseases: a Danish nationwide cohort study
Source: BMJ Open. 2024 Feb 21;14(2):e077408. doi: 10.1136/bmjopen-2023-077408 (PMC10882296; doi:10.1136/bmjopen-2023-077408)
Supplement: Author's manuscript [file bmjopen-2023-077408.draft_revisions.pdf]

BMJ Open is committed to open peer review. As part of this commitment we make the peer review history of every article we publish publicly available.

When an article is published we post the peer reviewers' comments and the authors' responses online. We also post the versions of the paper that were used during peer review. These are the versions that the peer review comments apply to.

The versions of the paper that follow are the versions that were submitted during the peer review process. They are not the versions of record or the final published versions. They should not be cited or distributed as the published version of this manuscript.

BMJ Open is an open access journal and the full, final, typeset and author-corrected version of record of the manuscript is available on our site with no access controls, subscription charges or pay-per-view fees (http://bmjopen.bmj.com).

If you have any questions on BMJ Open's open peer review process please email info.bmjopen@bmj.com

BMJ Open

The Impact of Immunosuppressive Therapy on SARS-CoV-2 mRNA Vaccine Effectiveness in Patients with Immune-mediated Inflammatory Diseases: A Danish Nationwide Cohort Study

Journal:	*BMJ Open*
Manuscript ID	bmjopen-2023-077408
Article Type:	Original research
Date Submitted by the Author:	04-Jul-2023

Complete List of Authors:	Elmahdi, Rahma; Statens Serum Institut, Epidemiology; Imperial College London, Equality, Diversity and Inclusion<br>Ward, Daniel; Center for Molecular Prediction of Inflammatory Bowel Disease,<br>Ernst, Martin; University of Southern Denmark, Clinical Pharmacology and Pharmacy, Department of Public Health<br>Poulsen, Gry; Aalborg University, Department of Clinical Medicine, Center for Molecular Prediction of Inflammatory Bowel Disease (PREDICT)<br>Hallas, Jesper; University of Southern Denmark, Clinical pharmacology and pharmacy<br>Pottegard, Anton; University of Southern Denmark, Clinical Pharmacology and Pharmacy, Department of Public Health<br>Jess, Tine; Aalborg Universitet, Center for Molecular Prediction of Inflammatory Bowel Disease, Department of Clinical Epidemiology; Statens Serum Institut, Department of Epidemiology Research

Keywords:	COVID-19, EPIDEMIOLOGY, Inflammatory bowel disease < GASTROENTEROLOGY, RHEUMATOLOGY, Psoriasis < DERMATOLOGY

SCHOLARONE™
Manuscripts

I, the Submitting Author has the right to grant and does grant on behalf of all authors of the Work (as defined in the below author licence), an exclusive licence and/or a non-exclusive licence for contributions from authors who are: i) UK Crown employees; ii) where BMJ has agreed a CC-BY licence shall apply, and/or iii) in accordance with the terms applicable for US Federal Government officers or employees acting as part of their official duties; on a worldwide, perpetual, irrevocable, royalty-free basis to BMJ Publishing Group Ltd ("BMJ") its licensees and where the relevant Journal is co-owned by BMJ to the co-owners of the Journal, to publish the Work in this journal and any other BMJ products and to exploit all rights, as set out in our licence.

The Submitting Author accepts and understands that any supply made under these terms is made by BMJ to the Submitting Author unless you are acting as an employee on behalf of your employer or a postgraduate student of an affiliated institution which is paying any applicable article publishing charge ("APC") for Open Access articles. Where the Submitting Author wishes to make the Work available on an Open Access basis (and intends to pay the relevant APC), the terms of reuse of such Open Access shall be governed by a Creative Commons licence – details of these licences and which Creative Commons licence will apply to this Work are set out in our licence referred to above.

Other than as permitted in any relevant BMJ Author's Self Archiving Policies, I confirm this Work has not been accepted for publication elsewhere, is not being considered for publication elsewhere and does not duplicate material already published. I confirm all authors consent to publication of this Work and authorise the granting of this licence.

For peer review only

The Impact of Immunosuppressive Therapy on SARS-CoV-2 mRNA Vaccine Effectiveness in Patients with Immune-mediated Inflammatory Diseases: A Danish Nationwide Cohort Study

Short Title: COVID-19 Vaccination in Immunosuppressed IMID Patients

Rahma Elmahdi[*1,2] (0000-0002-7013-9548), Daniel Ward[*1] (0000-0003-0405-7250), Martin Thomsen Ernst[3] (0000-0001-9003-3823), Gry Juul Poulsen[1] (0000-0003-2744-2469), Jesper Hallas[3] (0000-0002-8097-8708), Anton Pottegård[3] (0000-0001-9314-5679), Tine Jess[1,4] (0000-0002-4391-7332)

[*] Co-first author

Rahma Elmahdi, Clinical Research Associate, Center for the Molecular Prediction of Inflammatory Bowel Disease (PREDICT), Department of Clinical Medicine, Aalborg University, Denmark (1)

Doctor, Department of Lung and Infectious Disease Medicine, Nordsjællands Hospital, Hillerød, Denmark (2)

Daniel Ward, Doctoral Fellow, Center for the Molecular Prediction of Inflammatory Bowel Disease (PREDICT), Department of Clinical Medicine, Aalborg University, Denmark (1)

Martin Ernst Thomsen, Data Manager, Department of Public Health, Clinical Pharmacology, Pharmacy and Environmental Medicine, University of Southern Denmark, J.B. Winsløws Vej 19, 5000 Odense, Denmark (3)

Gry Juul Poulsen, Statistician, Center for the Molecular Prediction of Inflammatory Bowel Disease (PREDICT), Department of Clinical Medicine, Aalborg University, Denmark (1)

Jesper Hallas, Professor, Research Unit of Clinical Pharmacology, University of Southern Denmark, J.B. Winsløws Vej 9, 5000 Odense, Denmark (3)

Anton Pottegård, Professor, Department of Clinical Pharmacology and Pharmacy, University of Southern Denmark, J.B. Winsløws Vej 9, 5000 Odense, Denmark (3)

Tine Jess, Professor, Center for the Molecular Prediction of Inflammatory Bowel Disease (PREDICT), Department of Clinical Medicine, Aalborg University, Denmark (1)

Professor, Department of Gastroenterology and Hepatology, Aalborg University Hospital, Aalborg, Denmark (4)

For peer review only

For peer review only - http://bmjopen.bmj.com/site/about/guidelines.xhtml

Correspondence: Dr Daniel Ward, National Center of Excellence for the Molecular Prediction of Inflammatory Bowel Disease, PREDICT, Aalborg University, A. C. Meyers Vænge 15, DK-2450 Copenhagen, Denmark. E-mail: djwa@dcm.aau.dk

Key words: Immune-mediated inflammatory disease; Cohort study; SARS-CoV-2; Vaccination; Immunosuppressives

Word count: Abstract 250; manuscript 3020

For peer review only

For peer review only - http://bmjopen.bmj.com/site/about/guidelines.xhtml

ABSTRACT

Background: Investigation of SARS-CoV-2 vaccine efficacy in immune-mediated inflammatory disease (IMID) patients receiving immunosuppressives have been restricted to small sized serological studies. We therefore undertook an investigation of immunosuppressants' impact on real-world effectiveness of vaccines in these patient groups.

Methods: We performed a nationwide cohort study to assess the risk of COVID-19 infection in vaccinated IMID patients exposed to immunosuppressives compared to propensity score matched unexposed patients in the period from 1 January 2021 to 30 November 2021.Patients were followed from date of second vaccination, and weighted Cox models were used to estimate the risk of infection associated with immunosuppressives. Secondary outcomes included hospitalization and death associated with a positive SARS-CoV-2 test. Risk of infection by immunosuppressant drug class was also analysed.

Results: Immunosuppressants were associated with a significantly increased risk of infection overall (HR: 1.4, 95% CI: 1.2, 1.5), in inflammatory bowel disease (IBD) (HR: 1.6, 95% CI: 1.4, 1.9) and arthropathy (HR:1.3, 95% CI: 1.1, 1.4) but not psoriasis (HR: 1.1, 95% CI: 0.88, 1.4). Immunosuppressants were also associated with an increased risk of hospitalization overall (HR: 1.4, 95% CI: 1.0, 2.0), particularly in IBD (HR: 2.1, 95% CI: 1.0, 4.1). No significantly increased risk of death in immunosuppressants exposed patients was identified. Analyses by immunosuppressant drug class showed increased COVID-19 infection and hospitalization with anti-TNF, systemic corticosteroid, and rituximab exposure.

Conclusions: Immunosuppressive therapies reduced effectiveness of mRNA SARS-CoV-2 vaccination against infection and hospitalization in IMID patients. Anti-TNF, systemic corticosteroids, and rituximab were particularly associated with these risks.

For peer review only - http://bmjopen.bmj.com/site/about/guidelines.xhtml

INTRODUCTION

SARS-CoV-2 mRNA vaccines Comirnaty® (Pfizer-BioNTech) and Spikevax® (Moderna) were found to be efficacious in clinical trials prior to authorisation, and as of December 2022 over 758 million doses of Pfizer-BioNTech and 164 million doses of Moderna were administered in the EU/EAA (1). Pre-marketing trials excluded individuals considered at risk of immunocompromise, including those receiving immunosuppressive therapies (2,3), therefore there remains a paucity of data on the real-world effectiveness of SARS-CoV-2 vaccines in patients treated with immunosuppressive drugs.

Research published early in the SARS-CoV-2 epidemic suggested that, in the absence of vaccination, some types of immunosuppressives including rituximab, sulfasalazine, and corticosteroids are associated with an increased risk of severe outcomes in COVID-19 infection (4–8). Immune-mediated inflammatory diseases (IMID), including inflammatory bowel disease, inflammatory arthropathy, and psoriasis have themselves independently been associated with lower serological responses to SARS-CoV-2 vaccination than in healthy controls (9). Immunosuppressants are key therapies in IMID, so patients with IMID may be at increased risk of infection and severe outcomes of COVID-19 infection both due to the natural history of the diseases and the therapies used to treat them. Even in the context of second vaccination against SARS-CoV-2, exposure to immunosuppressives has been associated with a significantly poorer humoral response; lower than that which is required to confer immunity against infection and severe outcomes of COVID-19 infection in patients treated with immunosuppressive therapies (10–12).

It is therefore important to investigate the impact of immunosuppressants on SARS-CoV-2 vaccine effectiveness, while controlling for the underlying disease-indicating treatment, and other confounders that may impact vaccine effectiveness.

The real-world effectiveness of SARS-CoV-2 mRNA vaccinations against COVID-19 infection and associated outcomes such as hospitalization or death, in the context of immunosuppressive therapy exposure among IMID patients has not yet been investigated. The aim of this study was to use Danish nationwide population-based

For peer review only - http://bmjopen.bmj.com/site/about/guidelines.xhtml

data to assess the impact of immunosuppressive exposure on the risk of COVID-19 infection in three cohorts of vaccinated IMID patients.

METHODS

Data sources

We conducted a nationwide cohort study using the Danish COVID-19 cohort (13), based on data from the Danish Microbiology Registry (14), which includes individual-level information on vaccine type, dose, and date of administration; SARS-CoV-2 test type and date administered. This data was linked at the individual level to both the Danish National Patient Registry (15) and the Danish National Prescription Registry (16) using a unique Danish Civil Registration number (assigned to all individuals residing in Denmark). The Danish National Patient Registry, a register of hospital activities, includes medical diagnoses coded using International Classification of Disease (ICD-10), and medical procedures and prescriptions including treatment with intravenous medications. The Danish National Prescription Registry contains information on prescriptions dispensed at all community retail pharmacies, including date of dispensing, tablet strength, and pack sizes.

Population, follow-up, and outcomes

The IMID cohort comprised of three disease specific cohorts of all patients diagnosed with inflammatory bowel disease (IBD; ICD-10: K50, K51), inflammatory arthropathy (ICD-10: M45, M46, M05, M06, M07) or psoriasis (ICD-10: L40) in Denmark, who had received two doses of SARS-CoV-2 mRNA (Pfizer-BioNTech or Moderna) vaccine. Patients with more than one of these IMID diagnoses were included in only one cohort, with IBD taking precedence, then inflammatory arthropathy, finally psoriasis. Therefore, only patients with psoriasis and neither an IBD nor an inflammatory arthropathy diagnosis were included in the psoriasis cohort. This order was preferred as extent of organ-specific disease likely determines the dose for immunosuppressive therapy.

Patients were followed from the date of administration of second mRNA vaccine (the index date) after 1 January 2021. The primary outcome was a positive SARS-CoV-2 PCR test in the observation period.

For peer review only - http://bmjopen.bmj.com/site/about/guidelines.xhtml

Secondary outcomes were hospitalization between 7 days prior to and up to 28 days after a positive test or death within 60 days of a positive test (in Danish National Patient Register and Danish Civil Register). Follow-up was censored at administration of a third vaccination, emigration, death (in the absence of a positive SARS-CoV-2 test) or the end of the study period, 30 November 2021, as prevalence of the omicron variant became substantial after 28 November 2021(17).

Exposures

The exposures for this study include dispensed prescriptions or hospital administration of an immunosuppressive in the 120 days preceding the index date (date of administration of second vaccination). Immunosuppressants included selective immunosuppressants, anti-TNFs, interleukin inhibitors, calcineurin inhibitors, corticosteroids, rituximab and other immunosuppressants (see Supplementary Table 1 for complete list and ACT codes for immunosuppressants). The 120-day exposure window is chosen to cover the largest pack sizes of prescriptions which can contain medications for up to 120 days. A minimum daily dose of corticosteroids equivalent to 7.5 mg prednisolone per day was estimated as the entire dispensed quantity of corticosteroids during a sequence of prescriptions (within the 120-day exposure period) divided by the number of days from the first prescription to the index date. Unexposed IMID patients were defined as those with a diagnosis of one of the three IMID, who had not received an immunosuppressive in the 120 days preceding the index date, and those receiving <7.5 mg prednisolone-equivalent average per day.

Statistical models

Covariates included age (handled as a continuous covariate), sex, comorbidities (cardiovascular disease, pulmonary disease, liver disease, kidney disease, other gastrointestinal diseases, skin disease, and musculoskeletal disease), medications (cardiovascular drugs, antibiotics, oral anticoagulants, diabetes, and chronic airway disease medications). Testing frequency varied during the period studied due to changes in national and international guidelines and travel restrictions, along with the background prevalence of SARS-CoV-2, which could introduce bias in case detection. We therefore adjusted for individual testing frequency by including number of tests in the month preceding index date as a continuous covariate. Further, covariates

For peer review only - http://bmjopen.bmj.com/site/about/guidelines.xhtml

specific to each IMID were included separately for each cohort. For the IBD cohort this included any IBD-related hospital admissions in the previous year, Crohn's disease, ulcerative colitis, 5-ASA/sulfasalazine, budesonide, IBD-related procedures, and endoscopy of the gastrointestinal tract (see Supplementary Table 2 for complete list of IMID cohort specific covariates).

To balance the covariates in the exposed and unexposed groups, we fitted propensity score (PS) models for each IMID cohort separately. Propensity scores were calculated using logistic regression for the probability of exposure (treatment with immunosuppressives) conditional on the covariates defined above (18). We subsequently implemented the PS using standardized mortality ratio (SMR) weights (with trimming of subjects with extreme weights beyond 1st and 99th centiles). We assessed the distribution of covariates with standardized differences before and after PS weighting.

We used weighted Cox proportional-hazards regression models (19) to estimate risk of the COVID-19 infection in IMID patients exposed to immunosuppressive therapy compared to unexposed patients for each disease cohort separately. We used calendar time as the underlying time scale to account for period effects on the risk of the outcomes which may relate to varying infection prevalence and patient characteristics as patients vulnerable to severe outcomes were vaccinated earlier in the year. We then undertook Fixed Effects Model (FEM) meta-analysis to calculate the pooled HR of infection, hospitalization, and death for IBD, arthropathy and psoriasis cohorts as overall risk in immunosuppressive exposed IMID by COVID-19 outcome.

Finally, we undertook drug specific analysis for risk of COVID-19 infection by immunosuppressive drug class. In this analysis, patients receiving multiple immunosuppressive treatments were treated as independently exposed to each drug class. To account for the potential impact of immunosuppressants commonly prescribed in a weaning dose, which would not be captured using the definition of ≥7.5 mg dose equivalent per day, we undertook a sensitivity analysis to assess whether having any prescription for systemic corticosteroids over the 120-day period before the index date had an impact on the risk of infection, hospitalization, or death for those

For peer review only - http://bmjopen.bmj.com/site/about/guidelines.xhtml

exposed to this class of immunosuppressants. We used the statistical software Stata version 16.1 (StataCorp, College Station, TX, USA).

RESULTS

A total of 184,391 patients diagnosed with IBD, arthropathy or psoriasis were identified. Following exclusion of those not receiving two doses of SARS-CoV-2 mRNA vaccine, migration prior to receipt of second vaccination, and trimming of the population with extreme propensity scores, a total 152,440 patients were included, contributing a total 19,341 person-years of follow-up. During the 120-day exposure assessment period, 39,765 IMID patients received immunosuppressive treatment (10,480 IBD, 24,261 arthropathy, and 5,024 psoriasis), and 112,675 IMID patients (47,001 IBD, 44,669 arthropathy, and 21,005 psoriasis) patients did not. A total of 9 exposed and 51 unexposed IMID patients are censored from overall analysis due to migration or inclusion on the date of study end (therefore contributing no follow-up time). See Figure 1 of inclusion and exclusion flow chart. Following application of SMR weighting, the cohorts were balanced on the included covariates (see Table 1 for covariate prevalence and standardised differences [SD]).

A total of 866 (2.2%) COVID-19 infections were recorded among immunosuppressive exposed IMID patients during the follow-up period compared with 2,077 (1.8%) for unexposed IMID patients. A significantly increased weighted hazard for infection among exposed patients was seen for both IBD (HR: 1.6, 95% CI: 1.4, 1.9) and arthropathy (HR: 1.3, 95% CI: 1.1, 1.4) cohorts but not for the psoriasis cohort (HR: 1.1, 95 % CI: 0.88, 1.4). Meta-analysis of the three IMID cohorts showed a pooled HR for COVID-19 infection in exposed patients of 1.4 (95% CI: 1.2, 1.5; Figure 2). Fewer than 52 exposed and 122 unexposed IMID patients were hospitalized with COVID-19 infection during the follow-up period, which corresponded to a significantly increased risk of hospitalization overall for immunosuppressive exposed IMID patients (pooled HR: 1.4, 95% CI: 1.0, 2.0). Less than five immunosuppressive exposed IBD patients died in the 60 days following a COVID-19 diagnosis compared to six unexposed IBD patients. Six patients in the arthropathy cohort exposed to immunosuppressives compared with 13 unexposed arthropathy patients died in the 60 days following a COVID-19 diagnosis and fewer than five patients with a psoriasis diagnosis, either immunosuppressive

For peer review only - http://bmjopen.bmj.com/site/about/guidelines.xhtml

exposed or unexposed died with a COVID-19 diagnosis. These did not correspond to a significantly increased risk of death among exposed patients in any of the three cohorts or overall (pooled HR: 0.92, 95% CI: 0.38, 2.2; Figure 2).

Analysis for risk of infection among IMID cohorts by immunosuppressive drug class exposure showed a significantly increased risk of infection among users of anti-TNF (HR: 1.8, 95% CI: 1.6, 2.0), systemic corticosteroid (HR: 1.2, 95% CI: 1.0,1.5), and rituximab and other immunosuppressant (HR: 1.3, 95% CI: 1.1, 1.4). No other immunosuppressant was significantly associated with COVID-19 infection following second vaccination (Figure 3). Anti-TNF and systemic corticosteroid exposure were also associated with an increased risk of COVID-19 associated hospitalization (HR 1.8, 95% CI 1.0, 3.3 and HR 1.8, 95% CI 1.0,3.0, respectively; Figure 4). No immunosuppressive drug class was associated with death among IMID patients following receipt of second vaccination.

Sensitivity analysis assessing outcomes following any systemic corticosteroid exposure in the 120-day period prior to the index date showed no significant difference in risk of infection (crude HR: 1.2, 95% CI: 1.0, 1.4; adjusted HR: 1.1, 95% CI: 0.95, 1.3) or death (crude HR: 3.1, 95% CI: 1.4, 7.0; adjusted HR: 1.9, 95% CI: 0.77, 4.7) (Supplementary Table 6). However, risk of hospitalization following infection was significantly increased in those ever exposed to systemic corticosteroids in the 120-day period prior to receipt of second vaccination (crude HR: 2.8, 95% CI: 1.9, 4.1; adjusted HR: 2.1, 95% CI: 1.4, 3.2), showing similar results compared with analysis restricting to a ≥7.5 mg daily equivalent dose.

DISCUSSION

In this large nation-wide cohort study of three IMID patient cohorts, we identified a total of 39,755 immunosuppressive exposed patients matched to 112,619 immunosuppressive unexposed IMID patients to investigate the risk of COVID-19 infection, hospitalization, and death among IBD, arthropathy and psoriasis patients following second SARS-CoV-2 vaccination. Meta-analysis of the three cohorts showed an overall

For peer review only - http://bmjopen.bmj.com/site/about/guidelines.xhtml

35% increased risk of infection and, 42% increased risk of COVID-19 associated hospitalization in immunosuppressive exposed compared to immunosuppressive unexposed IMID patients.

Mortality was not significantly increased in immunosuppressive exposed patients as these events were rare. Drug class analysis showed anti-TNF, systemic corticosteroid, and rituximab and other immunosuppressant exposure was significantly associated with both increased risk of COVID-19 infection and hospitalization following second vaccination in immunosuppressive exposed compared to unexposed IMID patients.

We attempted to ascertain the effectiveness of SARS-CoV-2 mRNA vaccination among IMID patients exposed to immunosuppressive therapies, while controlling for the severity of the underlying disease indicating immunosuppressive treatment with a propensity score model. We found that immunosuppressants were associated with an increased risk of infection, likely due to the impact of immunosuppressive medication on vaccination against COVID-19 infection. This is particularly seen in IBD but is also present in arthropathy patients and, to a lesser, but not significant extent, in psoriasis. Similarly, when assessing risk of hospitalization following vaccination by immunosuppressive exposure, we find a significantly increased risk in IBD patients, which is not observed in arthropathy or psoriasis. The poorer outcomes observed in immunosuppressive exposed IBD patients have not been identified elsewhere. In a meta-analysis of serological response to SARS-CoV-2 vaccination among IMID treated patients, IBD patients were found to have a higher response to second mRNA vaccination dose than psoriasis or rheumatoid patients (event rate: 0.94, 95% CI: 0.86, 0.68; 0.9, 95% CI: 0.33, 0.99; 0.8, 95% CI: 0.68, 0.88 respectively) (20). This finding may be an indication that serological response is not a sufficient correlate of immunity for COVID-19 infection following vaccination, particularly in immunosuppressive treated patients. IBD patients typically present with more extensive disease, often necessitating higher doses of immunosuppressive therapy, over longer periods to achieve disease remission than that required for psoriasis or arthropathies (21–23). However, overall pooled IMID cohort meta-analysis showed a significantly increased risk for both these outcomes, indicating a general trend towards poorer outcomes in immunosuppressive exposed patients regardless of IMID cohort.

For peer review only - http://bmjopen.bmj.com/site/about/guidelines.xhtml

Reassuringly, immunosuppressant exposure was not associated with increased risk of death due to COVID-19 in any of the IMID patient cohorts, suggesting immunosuppressants do not reduce the effectiveness of vaccination in preventing this important outcome. However, caution should be exercised in the interpretation of this finding as deaths were recorded in either immunosuppressive exposed or unexposed patients and this may be due to the relatively short follow-up period of 11-months in this study.

Treatment with TNF-alpha inhibitors, systemic corticosteroid, and rituximab and other immunosuppressants was associated with a significantly increased risk of infection and hospitalization following receipt of second vaccination. This is consistent with previous studies, which suggests that treatment with cytokine inhibitors or B-cell depleting immunosuppressives is related to particularly poor COVID-19 outcomes (24–26), however the association with TNF-alpha inhibitors is novel. Our findings of increased risk of infection and hospitalization, but not death, in sensitivity analysis among IMID patients exposed to systemic corticosteroids is also in keeping with those other studies of unvaccinated IMID cohorts in Denmark (4) and internationally (6,27). These findings indicate that corticosteroid exposure weakens the protection conferred by vaccination. As glucocorticoids are known to inhibit the breadth of the immune response, including aspects of both the cellular and humoral immunity induced by mRNA vaccination, these findings appear to be intuitive (28). The interaction of IMID, the impact of treatments to control disease and response to vaccination, particularly considering the effects of dose and duration of administration is however complex (29,30) and the small number of hospitalization events, particularly in those receiving TNF-alpha inhibitors and systemic corticosteroid treatment means that these findings should be interpreted with caution. Further studies directly exploring the effects of vaccination whilst controlling for disease severity and exposure of immunosuppressive drugs by dose and duration would be required to disentangle the association of the different immunosuppressive drug classes with COVID-19 outcomes following vaccination. Such studies would also better inform guidance relating to timelines for SARS-CoV-2 vaccination in relation to the administration of immunosuppressive therapies in IMID patients.

For peer review only - http://bmjopen.bmj.com/site/about/guidelines.xhtml

One of the key strengths of this study is that it is large and population-representative, exploring the effectiveness of COVID-19 infection using real-world data from comprehensive, nationwide health registries. Vaccination does not directly correlate with protection from infection and the findings from this work provides important evidence on effectiveness of post-marketing mRNA vaccination in a vulnerable patient group. To our knowledge, this is the first study to assess the effectiveness of SARS-CoV-2 vaccination against COVID-19 infection, hospitalization, and mortality among IMID patients, based on immunosuppressive exposure. Additionally, our use of PS weighted regression models allows us to accurately control for the underlying treatment indicating disease, so we are better able to extrapolate the effects of the drug exposure from the disease itself. We restricted to a period of the pandemic where the delta variant was the dominant circulating strain of COVID-19 to ensure consistency in the assessment of our outcomes, although this limits extrapolation in the context of the omicron variant, or subsequent subvariants. Another potential limitation is the short calendar time that patients are followed up for infection, hospitalization, and mortality outcomes. We do however see a significantly increased risk of infection and hospitalization over the follow up time, which indicates that there was a sufficient follow-up period to detect a difference in poor COVID-19 outcomes. Although it is difficult to define a reliable threshold for which we consider a patient unexposed to immunosuppressive medication, it is reassuring that only approximately 12% of our unexposed group had filled a prescription in the 365 days prior to the 120-day exposure window assessed, and that removing the minimum threshold of 7.5 mg of systemic corticosteroids as an exposure requirement did not change our findings. A lack of individual-level data relating to confounders such as smoking behaviour, socio-economic status and dose of drug therapies could potentially limit our findings. There may also be a residual effect of confounding due to unmeasured disease severity not completely accounted for in our PS model. However, these are unlikely to systemically impact the direction of association or strength of significance identified in the risk of infection due to immunosuppressive exposure observed here.

In conclusion, our findings suggest a reduced effectiveness of mRNA SARS-CoV-2 vaccination against COVID-19 infection and hospitalization in IMID patients receiving immunosuppressive therapies. This risk is

For peer review only - http://bmjopen.bmj.com/site/about/guidelines.xhtml

particularly seen in IBD and arthropathy patients and is associated with anti-TNF, systemic corticosteroids, and rituximab and other immunosuppressant exposure.

Author Contributions: DW, RE, AP and TJ developed the study protocol. ME undertook primary data analysis with support from GP. DW and RE were responsible for first draft of the manuscript. All authors were responsible for interpretation of results and critical revisions to the final manuscript.

Acknowledgements: Funding Sources - This work was funded by grants from the Novo Nordisk Foundation (NNF21OC0068631) and the Danish National Research Foundation (DNRF-148).

Conflict of Interest Statement: All authors have none to declare.

For peer review only

REFERENCES

1. European Medicines Agency. *COVID-19 vaccines safety update: 8 December 2022 Rev. 1*. [Accessed 8th February 2023]. www.ema.europa.eu.

2. El Sahly HM, Baden LR, Essink B, Doblecki-Lewis S, Martin JM, Anderson EJ, et al. Efficacy of the mRNA-1273 SARS-CoV-2 Vaccine at Completion of Blinded Phase. *New England Journal of Medicine*. 2021;385(19): 1774–1785. https://doi.org/10.1056/nejmoa2113017.

3. Polack FP, Thomas SJ, Kitchin N, Absalon J, Gurtman A, Lockhart S, et al. Safety and Efficacy of the BNT162b2 mRNA Covid-19 Vaccine. *New England Journal of Medicine*. 2020;383(27): 2603–2615. https://doi.org/10.1056/NEJMOA2034577/SUPPL_FILE/NEJMOA2034577_PROTOCOL.PDF.

4. Ward D, Gørtz S, Ernst MT, Andersen NN, Kjær SK, Hallas J, et al. The effect of immunosuppressants on the prognosis of COVID-19 infection. *The European respiratory journal*. 2022;59(4). https://doi.org/10.1183/13993003.00769-2021.

5. Gianfrancesco M, Hyrich KL, Al-Adely S, Al-Adely S, Carmona L, Danila MI, et al. Characteristics associated with hospitalization for COVID-19 in people with rheumatic disease: data from the COVID-19 Global Rheumatology Alliance physician-reported registry. *Annals of the rheumatic diseases*. 2020;79(7): 859–866. https://doi.org/10.1136/ANNRHEUMDIS-2020-217871.

6. Brenner EJ, Ungaro RC, Gearry RB, Kaplan GG, Kissous-Hunt M, Lewis JD, et al. Corticosteroids, But Not TNF Antagonists, Are Associated With Adverse COVID-19 Outcomes in Patients With Inflammatory Bowel Diseases: Results From an International Registry. *Gastroenterology*. 2020;159(2): 481-491.e3. https://doi.org/10.1053/J.GASTRO.2020.05.032.

7. Salter A, Fox RJ, Newsome SD, Halper J, Li DKB, Kanellis P, et al. Outcomes and Risk Factors Associated With COVID-19 infection in a North American Registry of Patients With Multiple Sclerosis. *JAMA neurology*. 2021;78(6): 699–708. https://doi.org/10.1001/JAMANEUROL.2021.0688.

8. Strangfeld A, Schäfer M, Gianfrancesco MA, Lawson-Tovey S, Liew JW, Ljung L, et al. Factors associated with COVID-19-related death in people with rheumatic diseases: results from the COVID-19 Global Rheumatology Alliance physician-reported registry. *Annals of the Rheumatic Diseases*. 2021;80(7): 930–942. https://doi.org/10.1136/ANNRHEUMDIS-2020-219498.

9. Lee ARY bin, Wong SY, Chai LYA, Lee SC, Lee MX, Muthiah MD, et al. Efficacy of covid-19 vaccines in immunocompromised patients: systematic review and meta-analysis. *BMJ (Clinical research ed.)*. 2022;376: e068632. https://doi.org/10.1136/BMJ-2021-068632.

10. Boekel L, Steenhuis M, Hooijberg F, Besten YR, van Kempen ZLE, Kummer LY, et al. Antibody development after COVID-19 vaccination in patients with autoimmune diseases in the Netherlands: a substudy of data from two prospective cohort studies. *The Lancet. Rheumatology*. 2021;3(11): e778–e788. https://doi.org/10.1016/S2665-9913(21)00222-8.

11. Alexander JL, Kennedy NA, Ibraheim H, Anandabaskaran S, Saifuddin A, Castro Seoane R, et al. COVID-19 vaccine-induced antibody responses in immunosuppressed patients with inflammatory bowel disease (VIP): a multicentre, prospective, case-control study. *The lancet. Gastroenterology & hepatology*. 2022;7(4): 342–352. https://doi.org/10.1016/S2468-1253(22)00005-X.

For peer review only - http://bmjopen.bmj.com/site/about/guidelines.xhtml

12. de Boer SE, Berger SP, van Leer–Buter CC, Kroesen BJ, van Baarle D, Sanders JSF. Enhanced Humoral Immune Response After COVID-19 Vaccination in Elderly Kidney Transplant Recipients on Everolimus Versus Mycophenolate Mofetil-containing Immunosuppressive Regimens. *Transplantation*. 2022;106(8). https://doi.org/10.1097/TP.0000000000004177.

13. Pottegård A, Kristensen KB, Reilev M, Lund LC, Ernst MT, Hallas J, et al. Existing Data Sources in Clinical Epidemiology: The Danish COVID-19 Cohort. *Clinical epidemiology*. 2020;12: 875–881. https://doi.org/10.2147/CLEP.S257519.

14. Voldstedlund M, Haarh M, Mølbak K. The Danish Microbiology Database (MiBa) 2010 to 2013. *Euro surveillance : bulletin Europeen sur les maladies transmissibles = European communicable disease bulletin*. 2014;19(1). https://doi.org/10.2807/1560-7917.ES2014.19.1.20667.

15. Schmidt M, Schmidt SAJ, Sandegaard JL, Ehrenstein V, Pedersen L, Sørensen HT. The Danish National Patient Registry: a review of content, data quality, and research potential. *Clinical epidemiology*. 2015;7: 449–490. https://doi.org/10.2147/CLEP.S91125.

16. Pottegård A, Schmidt SAJ, Wallach-Kildemoes H, Sørensen HT, Hallas J, Schmidt M. Data Resource Profile: The Danish National Prescription Registry. *International journal of epidemiology*. 2017;46(3): 798. https://doi.org/10.1093/IJE/DYW213.

17. Espenhain L, Funk T, Overvad M, Edslev SM, Fonager J, Ingham AC, et al. Epidemiological characterisation of the first 785 SARS-CoV-2 Omicron variant cases in Denmark, December 2021. *Euro surveillance : bulletin Europeen sur les maladies transmissibles = European communicable disease bulletin*. 2021;26(50). https://doi.org/10.2807/1560-7917.ES.2021.26.50.2101146.

18. Rosenbaum PR, Rubin DB. The central role of the propensity score in observational studies for causal effects. *Biometrika*. 1983;70(1): 41–55. https://doi.org/10.1093/BIOMET/70.1.41.

19. Desai RJ, Franklin JM. Alternative approaches for confounding adjustment in observational studies using weighting based on the propensity score: a primer for practitioners. *BMJ*. 2019;367. https://doi.org/10.1136/BMJ.L5657.

20. Sakuraba A, Luna A, Micic D. Serologic Response to Coronavirus Disease 2019 (COVID-19) Vaccination in Patients With Immune-Mediated Inflammatory Diseases: A Systematic Review and Meta-analysis. *Gastroenterology*. 2022;162(1): 88-108.e9. https://doi.org/10.1053/J.GASTRO.2021.09.055.

21. Singh JA, Guyatt G, Ogdie A, Gladman DD, Deal C, Deodhar A, et al. 2018 American College of Rheumatology/National Psoriasis Foundation Guideline for the Treatment of Psoriatic Arthritis: *https://doi.org/10.1177/2475530318812244*. 2018;4(1): 31–58. https://doi.org/10.1177/2475530318812244.

22. Larsen L, Jensen MD, Larsen MD, Nielsen RG, Thorsgaard N, Vind I, et al. The Danish National Registry for Biological Therapy in Inflammatory Bowel Disease. *Clinical epidemiology*. 2016;8: 607–612. https://doi.org/10.2147/CLEP.S99478.

23. Cottone M, Sapienza C, Macaluso FS, Cannizzaro M. Psoriasis and Inflammatory Bowel Disease. *Digestive diseases (Basel, Switzerland)*. 2019;37(6). https://doi.org/10.1159/000500116.

24. Hernán MA. The hazards of hazard ratios. *Epidemiology*. 2010;21(1): 13–15. https://doi.org/10.1097/EDE.0B013E3181C1EA43.

For peer review only - http://bmjopen.bmj.com/site/about/guidelines.xhtml

25. Izadi Z, Brenner EJ, Mahil SK, Dand N, Yiu ZZN, Yates M, et al. Association Between Tumor Necrosis Factor Inhibitors and the Risk of Hospitalization or Death Among Patients With Immune-Mediated Inflammatory Disease and COVID-19. *JAMA network open*. 2021;4(10). https://doi.org/10.1001/JAMANETWORKOPEN.2021.29639.

26. Simon D, Tascilar K, Kleyer A, Fagni F, Krönke G, Meder C, et al. Impact of Cytokine Inhibitor Therapy on the Prevalence, Seroconversion Rate, and Longevity of the Humoral Immune Response Against SARS-CoV-2 in an Unvaccinated Cohort. *Arthritis & rheumatology (Hoboken, N.J.)*. 2022;74(5): 783–790. https://doi.org/10.1002/ART.42035.

27. Simon D, Tascilar K, Fagni F, Krönke G, Kleyer A, Meder C, et al. SARS-CoV-2 vaccination responses in untreated, conventionally treated and anticytokine-treated patients with immune-mediated inflammatory diseases. *Annals of the rheumatic diseases*. 2021;80(10): 1312–1316. https://doi.org/10.1136/ANNRHEUMDIS-2021-220461.

28. Favalli EG, Bugatti S, Klersy C, Biggioggero M, Rossi S, de Lucia O, et al. Impact of corticosteroids and immunosuppressive therapies on symptomatic COVID-19 infection in a large cohort of patients with chronic inflammatory arthritis. *Arthritis research & therapy*. 2020;22(1). https://doi.org/10.1186/S13075-020-02395-6.

29. McKay LI, Cidlowski JA. Physiologic and Pharmacologic Effects of Corticosteroids. 2003; https://www.ncbi.nlm.nih.gov/books/NBK13780/

30. Rahier JF, Moutschen M, van Gompel A, van Ranst M, Louis E, Segaert S, et al. Vaccinations in patients with immune-mediated inflammatory diseases. *Rheumatology (Oxford, England)*. 2010;49(10): 1815–1827. https://doi.org/10.1093/RHEUMATOLOGY/KEQ183.

31. Croce E, Hatz C, Jonker EF, Visser LG, Jaeger VK, Bühler S. Safety of live vaccinations on immunosuppressive therapy in patients with immune-mediated inflammatory diseases, solid organ transplantation or after bone-marrow transplantation - A systematic review of randomized trials, observational studies and case reports. *Vaccine*. 2017;35(9): 1216–1226. https://doi.org/10.1016/J.VACCINE.2017.01.048.

For peer review only

For peer review only - http://bmjopen.bmj.com/site/about/guidelines.xhtml

Table 1. Characteristics of immune-mediated inflammatory disease patients at baseline and after propensity score weighting, by exposure to immunosuppressive therapy.

	**Baseline IMID Cohort***			**Weighted IMID Cohort**		
	**Unexposed**	**Exposed**	**SD**	**Unexposed**	**Exposed**	**SD**
**Total, n (%)**	112 675 (100)	39 765 (100)	NA	39 524 (100)	39 765 (100)	NA
**Inflammatory bowel disease, n (%)**	47 001 (41.7)	10 480 (26.4)	NA	10 284 (26.0)	10 480 (26.4)	NA
**Arthropathy, n (%)**	44 669 (39.6)	24 261 (61.0)	NA	24 227 (61.3)	24 261 (61.0)	NA
**Psoriasis, n (%)**	21 005 (18.6)	5 024 (12.6)	NA	5 014 (12.7)	5 024 (12.6)	NA
Age, median (IQR)	59 (46-71)	58 (45-71)	0.06	58 (44-71)	58 (45-71)	0.01
Male, n (%)	48 941 (43.4)	17 080 (43.0)	0.01	16 934 (42.8)	17 080 (43.0)	0.00
SARS-CoV-2 test in the previous month, median (IQR)	0 (0-1)	0 (0-1)	0.04	0 (0-1)	0 (0-1)	0.00
**Comorbidities, n (%)**						
Cardiovascular disease	41 056 (36.4)	14 010 (35.2)	0.03	14 050 (35.5)	14 010 (35.2)	0.01
Pulmonary disease	14 994 (13.3)	5 793 (14.6)	0.04	5796 (14.7)	5 793 (14.6)	0.00
Liver disease	3 630 (3.2)	1 528 (3.8)	0.03	1 525 (3.9)	1 528 (3.8)	0.00
Kidney disease	6 859 (6.1)	2 260 (5.7)	0.02	2 279 (5.8)	2 260 (5.7)	0.00
Other gastrointestinal diseases	21 505 (19.1)	7 086 (17.8)	0.03	6 984 (17.7)	7 086 (17.8)	0.00
Skin disease	9 889 (8.8)	3 748 (9.4)	0.02	3 703 (9.4)	3 748 (9.4)	0.00
Musculoskeletal disease	45 393 (40.3)	16 620 (41.8)	0.03	16 649 (42.1)	16 620 (41.8)	0.01
**Medications, n (%)**						
Cardiovascular drugs	80 529 (71.5)	28 179 (70.9)	0.01	28 054 (71.0)	28 179 (70.9)	0.00
Antibiotics	108 502 (96.3)	38 405 (96.6)	0.02	38 181 (96.6)	38 405 (96.6)	0.00
Oral anticoagulants	11 111 (9.9)	4 022 (10.1)	0.01	4 034 (10.2)	4 022 (10.1)	0.00
Drugs used in diabetes	12 935 (11.5)	4 034 (10.1)	0.04	4 030 (10.2)	4 034 (10.1)	0.00
Drugs for obstructive airway diseases	36 970 (32.8)	13 118 (33.0)	0.00	13 116 (33.2)	13 118 (33.0)	0.00
**IBD-specific treatments, n (%)**						
Any IBD-related hospital admissions in the previous year	564 (1.2)	714 (6.8)	0.12	584 (5.7)	714 (6.8)	0.05
5-ASA/sulfasalazine	12 596 (26.8)	2 845 (27.1)	0.00	2 857 (27.8)	2 845 (27.1)	0.01
Budesonide	646 (1.4)	272 (2.6)	0.02	277 (2.7)	272 (2.6)	0.01
IBD related procedures	18 553 (39.5)	4 551 (43.4)	0.05	4 473 (43.5)	4 551 (43.4)	0.00

For peer review only - http://bmjopen.bmj.com/site/about/guidelines.xhtml

Endoscopy of the gastrointestinal tract	7 454 (15.9)	3 702 (35.3)	0.12	3 543 (34.5)	3 702 (35.3)	0.02
**Arthropathy-specific treatments, n (%)**						
Arthropathy-related procedures	13 932 (31.2)	8 415 (34.7)	0.09	8 399 (34.7)	8 415 (34.7)	0.00
Anti-inflammatory and anti-rheumatic drugs	5 678 (12.7)	3 462 (14.3)	0.07	3 480 (14.4)	3 462 (14.3)	0.00
Hydroxychloroquine	540 (1.2)	845 (3.5)	0.15	801 (3.3)	845 (3.5)	0.01
**Psoriasis-specific treatments, n (%)**						
5-ASA/sulfasalazine	2 149 (10.2)	807 (16.1)	0.03	805 (16.1)	807 (16.1)	0.00
Topical corticosteroids	73 (0.3)	42 (0.8)	0.00	39 (0.8)	42 (0.8)	0.01
Anti-psoriatic medication	7 441 (35.4)	1 894 (37.7)	0.01	1 900 (37.9)	1 894 (37.7)	0.00
Topical calcineurin inhibitors	3 731 (17.8)	1 106 (22.0)	0.00	1 110 (22.1)	1 106 (22.0)	0.00
Psoriasis related procedures	542 (2.6)	144 (2.9)	0.00	144 (2.9)	144 (2.9)	0.00

Abbreviations: IMID = immune-mediated inflammatory disease; SD = standardized differences; IQR = inter-quartile range; NA = not applicable. *Total cohort numbers prior to trimming.

FIGURE LEGENDS

Figure 1. Flow chart for inclusion into the IMID cohort.

Figure 2. Risk of infection, hospitalization and death associated with immunosuppressive exposure from Jan 2021-Nov 2021 in Inflammatory Bowel Disease, Arthropathy, Psoriasis and meta-analysis across all IMID cohorts.

Abbreviations: IMID=immune-mediated and inflammatory disease; IBD=inflammatory bowel disease; w=weighted; HR=hazard ratio; 95%-CI=95% confidence interval

Figure 3. Risk of infection associated with immunosuppressive drug class exposure (Selective Immunosuppressants, Tumour Necrosis Factor Inhibitors, Interleukin Inhibitors, Calcineurin Inhibitors, Systemic Corticosteroids, and Other Immunosuppressants including Rituximab).

Abbreviations: HR=hazard ratio; 95%-CI=95% confidence interval

Figure 4. Risk of hospitalization associated with immunosuppressive drug class exposure (Selective Immunosuppressants, Tumour Necrosis Factor Inhibitors, Interleukin Inhibitors, Calcineurin Inhibitors, Systemic Corticosteroids, and Other Immunosuppressants including Rituximab).

Abbreviations: HR=hazard ratio; 95%-CI=95% confidence interval

For peer review only

Figure 1.

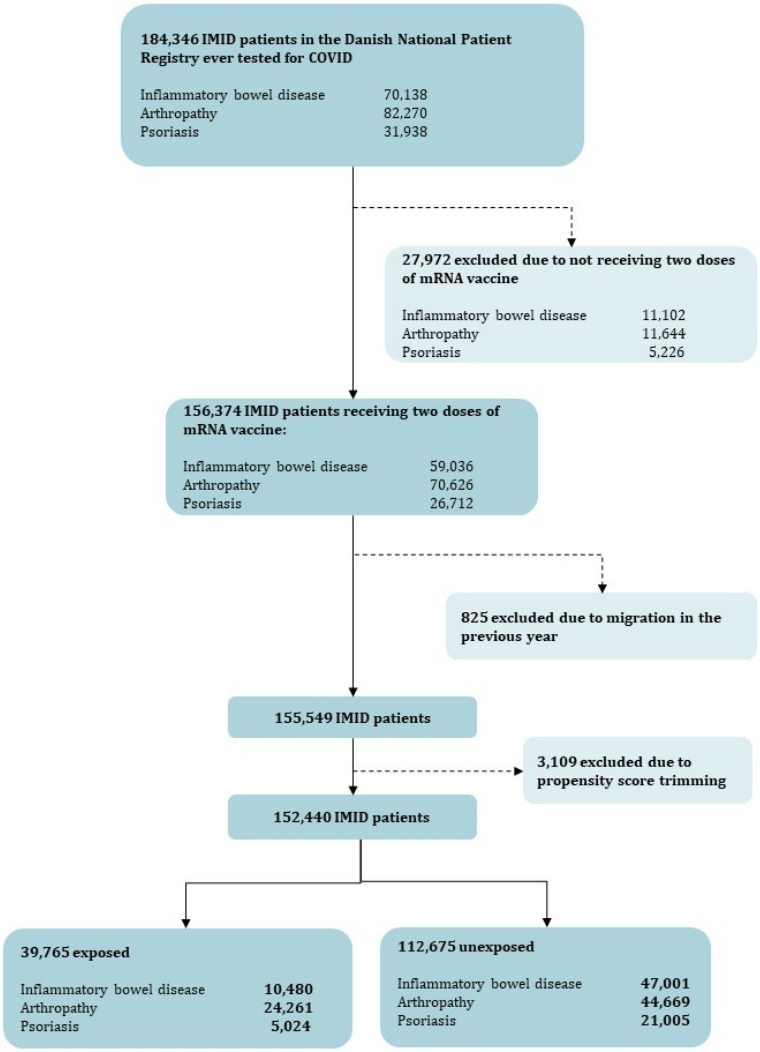

190x275mm (96 x 96 DPI)

Figure 2.

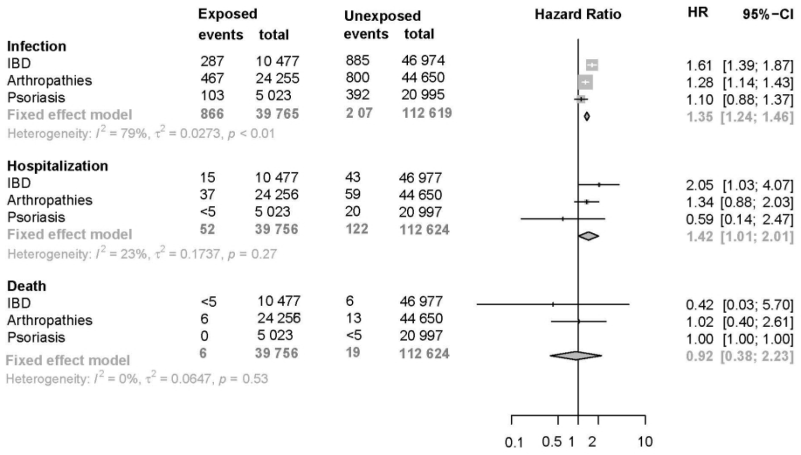

296x209mm (200 x 200 DPI)

Figure 3.

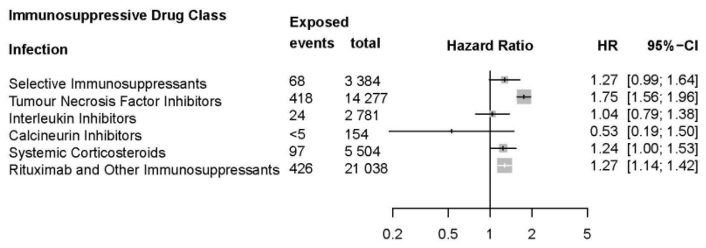

Figure 3. Risk of infection associated with immunosuppressive drug class exposure (Selective Immunosuppressants, Tumour Necrosis Factor Inhibitors, Interleukin Inhibitors, Calcineurin Inhibitors, Systemic Corticosteroids, and Other Immunosuppressants including Rituximab) in IMID patients.

296x209mm (200 x 200 DPI)

Figure 4.

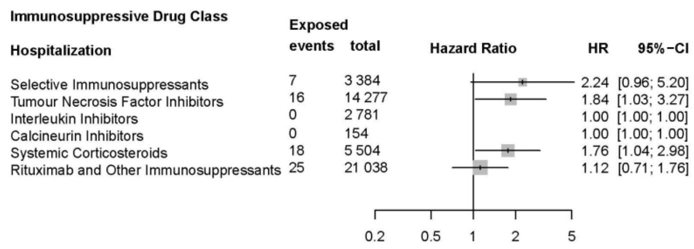

Figure 4. Risk of hospitalization associated with immunosuppressive drug class exposure (Selective Immunosuppressants, Tumour Necrosis Factor Inhibitors, Interleukin Inhibitors, Calcineurin Inhibitors, Systemic Corticosteroids, and Other Immunosuppressants including Rituximab) in IMID patients.

296x209mm (200 x 200 DPI)

The Impact of Immunosuppressive Therapy on SARS-CoV-2 mRNA Vaccine Effectiveness in Patients with Immune-mediated Inflammatory Diseases: A Danish Nationwide Cohort Study

Rahma Elmahdi*[1,2] (0000-0002-7013-9548), Daniel Ward*[1] (0000-0003-0405-7250), Martin Thomsen Ernst[3] (0000-0001-9003-3823), Gry Juul Poulsen[1] (0000-0003-2744-2469), Jesper Hallas[3] (0000-0002-8097-8708), Anton Pottegård[3] (0000-0001-9314-5679), Tine Jess[1,4] (0000-0002-4391-7332)

Supplementary Tables

Supplementary Table 1. Exposures and corresponding ATC and procedure codes for immunosuppressives

Medication	ATC code	Procedure code
**Selective immunosuppressants**		
Muromonab-CD3	L04AA02	
Antilymphocyte immunoglobulin (horse)	L04AA03	
	L04AA04	BOHJ12
Antithymocyte immunoglobulin (rabbit)	L04AA06	BOHJ22
Mycophenolic acid	L04AA10	BOHJ23
Sirolimus	L04AA13	
Leflunomide	L04AA18	BOHJ24
Everolimus	L04AA23	BOHJ26
Natalizumab	L04AA24	BOHJ18C1
Abatacept	L04AA25	BWHB84
Eculizumab	L04AA26	BOHJ19H6
Belimumab	L04AA27	BOHJ27
Fingolimod	L04AA28	
Belatacept	L04AA29	BOHJ28D
Tofacitinib	L04AA31	BOHJ28A
Teriflunomide	L04AA32	
Aprelimast	L04AA33	BOHJ19H4
Vedolizumab	L04AA34	BOHJ16A
Alemtuzumab	L04AA36	
Ocrelizumab	L04AA37	
Baricitinib	L04AA38	
Ozanimod	L04AA39	
Emapalumab	L04AA40	BWHA178
Cladribine	L04AA41	
Imlifidase	L04AA42	BWHB87
Siponimod	L04AA43	

For peer review only

Ravulizumab	L04AA44	
Upadacitinib		
**Tumour necrosis factor inhibitors**		
Etanercept	L04AB01	BOHJ18A2
Infliximab	L04AB02	BOHJ18A1
Adalimumab	L04AB04	BOHJ18A3
Certolizumab pegol	L04AB05	BOHJ18A5
Golimumab	L04AB06	BOHJ18A4
**Interleukin inhibitors**		
Basiliximab	L04AC02	
Anakinra	L04AC03	BOHJ18B1
Ustekinumab	L04AC05	BOHJ18B3
Tocilizumab	L04AC07	BOHJ18B2
Canakinumab	L04AC08	BOHJ18B4
Secukinumab	L04AC10	BOHJ18B5
Siltuximab	L04AC11	
Brodalumab	L04AC12	BOHJ18B6
Ixekizumab	L04AC13	
Sarilumab	L04AC14	BOHJ18B9
Guselkumab	L04AC16	
Tildrakizumab	L04AC17	BOHJ18B7
Risankizumab	L04AC18	BOHJ19N1
**Calcineurin inhibitors**		
Ciclosporin	L04AD01	BOHJ20
Tacrolimus	L04AD02	BOHJ21
**Other immunosuppressants**		
Azathioprine	L04AX01	BWHB83
Thalidomide	L04AX02	BWHB81
Methotrexate	L04AX03	BWHA115
Lenalidomide	L04AX04	BWHB82
Pirfenidone	L04AX05	BWHB85
Pomalidomide	L04AX06	BWHB86
Dimethyl fumarate	L04AX07	BOHJ28B
Darvadstrocel	L04AX08	
**Systemic corticosteroids**	H02AB	
**Rituximab**	L01XC02	

For peer review only

Supplementary Table 2. Covariates by disease cohort

IBD COHORT		
**Covariate**	**Categories/ ATC/ICD codes**	**Assessment window**
Age	-	At cohort entry
Sex	-	At cohort entry
SARS-CoV-2 tests in the previous month	-	At cohort entry
Ever previous SARS-CoV-2 positive test	-	From February 2020
IBD specific covariates		
Any IBD–related hospital admissions in the previous year	K50/K51	1 year before cohort entry
Crohn's disease	K50	At cohort entry
Ulcerative colitis	K51	At cohort entry
5-ASA/sulfasalazine	MA07EC0	1 year before matching date
Budesonide	MA07EA06	3 months before matching date
IBD related procedures		Any time before cohort entry
Operations of the small bowel and colon	KJF	
Operations of the rectum	KJG	
Operations of the anus and perianal tissue	KJH	
Operational of the abdominal wall, peritoneum, mesentery and omentum	KJA	
Lysis of adhesion in the abdominal cavity	KJAP	
Closure of intestinovaginal fistula	KLEE30	
Closure of vesiculointestinal fisutal	KKCH30	
Endoscopy of the gastrointestinal tract	KUJ	1 year before the cohort entry
Comorbidities		
Cardiovascular disease	DI1-I7	Any time before cohort entry
Pulmonary disease		Any time before cohort entry
Chronic disease of the lower airways	DJ4	
Other interstitial lung disease	DJ84	
Diseases with pus and necrosis in the lower airway	DJ85	
Interstitial lung emphysema	DJ982	
compensatory emphysema	DJ983	
Liver disease	DK70-K77	Any time before cohort entry
Kidney disease		Any time before cohort entry
Glomerular disease	DN0	
Tubulointerstitial kidney disease and kidney insufficiency	DN1	
Other gastrointestinal diseases		Any time before cohort entry
Gingivitis and periodontal disease	DK05	
Inflammation of the oral mucosa and related	DK12	
Stomach and duodenal ulcers	DK25-27	
Fissure and rifts in and around the anus	DK60	
Abscess in and around the anus	DK61	
Other diseases of the rectum and anus	DK62	
Other bowel disease	DK63	

Skin disease<br>Bullous skin disease<br>Dermatitis and eczema<br>Alopecia areata<br>Vitiligo<br>Granulomatous disease in the skin and subcutaneous tissue<br>Lupus<br>Other localised connective tissue disease<br>Vasculitis limited to the skin	 DL10-14<br>DL20-30<br>DL63<br>DL80<br>DL92<br>DL93<br>DL94<br>DL95	Any time before cohort entry
Musculoskeletal disease<br>Generalised connective tissue diseases<br>Diseases of the muscles<br>Soft-tissue rheumatism	 DM30-<br>DM36<br>DM60-63<br>DM70-79	Any time before cohort entry
Medications		
Cardiovascular drugs	MC01-MC10	Any time before cohort entry
Antibiotics	MJ01	Any time before cohort entry
Oral anticoagulants	MB01AA<br>MB01AF	Any time before cohort entry
Drugs used in diabetes	MA10	Any time before cohort entry
Drugs for obstructive airway diseases	MR03	Any time before cohort entry
INFLAMMATORY ARTHROPATHIES COHORT		
Age	-	At cohort entry
Sex	-	At cohort entry
SARS-CoV-2 tests in the previous month	-	At cohort entry
Arthropathy-specific covariates		
Arthropathy-related procedures<br>Shoulder and upper arm<br>Primary insertion of joint prosthesis<br>Secondary insertion of joint prosthesis<br>Operations on the joint capsule and ligaments<br>Operations of the synovia and joint surface<br>Joint resections, arthroplasties, and arthrodesis<br>Excision of bursa<br>Elbow and lower arm<br>Primary insertion of joint prosthesis<br>Secondary insertion of joint prosthesis<br>Operations on the joint capsule and ligaments<br>Operations of the synovia and joint surface<br>Joint resections, arthroplasties, and arthrodesis<br>Excision of bursa<br>Wrist and hand<br>Primary insertion of joint prosthesis<br>Secondary insertion of joint prosthesis<br>Operations on the joint capsule and ligaments<br>Operations of the synovia and joint surface<br>Joint resections, arthroplasties, and arthrodesis<br>Operations on the fascia, tendon sheaths, ganglia and bursae	 <br>KNBB<br>KNBC<br>KNBE<br>KNBF<br>KNBG<br>KBM79<br><br>KNCB<br>KNCC<br>KNCE<br>KNCF<br>KNCG<br>KNCM79<br><br>KNDB<br>KNDC<br>KNDE<br>KNDF<br>KNDG<br>KNDM	Any time before cohort entry

For peer review only

Hip and thigh		
Primary insertion of joint prosthesis	KNFB	
Secondary insertion of joint prosthesis	KNFC	
Operations on the joint capsule and ligaments	KNFE	
Operations of the synovia and joint surface	KNFF	
Joint resections, arthroplasties, and arthrodesis	KNFG	
Excision of bursa in the	KNFM79	
Knee and lower leg		
Primary insertion of joint prosthesis	KNGB	
Secondary insertion of joint prosthesis	KNGC	
Operations on the joint capsule and ligaments	KNGE	
Operations of the synovia and joint surface	KNGF	
Joint resections, arthroplasties, and arthrodesis	KNGG	
Excision of bursae	KNGM79	
Ankle and foot		
Primary insertion of joint prosthesis	KNHB	
Secondary insertion of joint prosthesis	KNHC	
Operations on the joint capsule and ligaments	KNHE	
Operations of the synovia and joint surface	KNHF	
Joint resections, arthroplasties, and arthrodesis	KNHG	
Operations on the fascia, tendon sheaths, ganglia and bursae	KNHM	
Anti-inflammatory and anti-rheumatic drugs inc. specific anti-rheumatic therapies, non-steroidals, and combination medications	M01	3 months before before cohort entry
Hydroxychloroquine	P01BA02	
Comorbidities		
Cardiovascular disease	DI1-I7	Any time before cohort entry
Pulmonary disease		Any time before cohort entry
Chronic disease of the lower airways	DJ4	
Other interstitial lung disease	DJ84	
Diseases with pus and necrosis in the lower airway	DJ85	
Interstitial lung emphysema	DJ982	
compensatory emphysema	DJ983	
Liver disease	DK70-K77	Any time before cohort entry
Kidney disease		Any time before cohort entry
Glomerular disease	DN0	
Tubulointerstitial kidney disease and kidney insufficiency	DN1	
Other gastrointestinal diseases		Any time before cohort entry
Gingivitis and periodontal disease	DK05	
Inflammation of the oral mucosa and related	DK12	
Stomach and duodenal ulcers	DK25-27	
Fissure and rifts in and around the anus	DK60	
Abscess in and around the anus	DK61	
Other diseases of the rectum and anus	DK62	
Other bowel disease	DK63	
Skin disease		Any time before cohort entry
Bullous skin disease	DL10-14	
Dermatitis and eczema	DL20-30	
Alopecia areata	DL63	

For peer review only

Vitiligo<br>Granulomatous disease in the skin and subcutaneous tissue<br>Lupus<br>Other localised connective tissue disease<br>Vasculitis limited to the skin	DL80<br>DL92<br>DL93<br>DL94<br>DL95	
Musculoskeletal disease<br>Generalised connective tissue diseases<br>Diseases of the muscles<br>Soft-tissue rheumatism	 DM30-<br>DM36<br>DM60-63<br>DM70-79	Any time before cohort entry
Medications		
Cardiovascular drugs	MC01-MC10	Any time before cohort entry
Antibiotics	MJ01	Any time before cohort entry
Oral anticoagulants	MB01AA<br>MB01AF	Any time before cohort entry
Drugs used in diabetes	MA10	Any time before cohort entry
Drugs for obstructive airway diseases	MR03	Any time before cohort entry
PSORIASIS COHORT		
Age	-	At cohort entry
Sex	-	At cohort entry
SARS-CoV-2 tests in the previous month	-	At cohort entry
Psoriasis-specific covariates		
Procedures<br>Ultraviolet therapy: UV-A with psoralen/broad-spectrum UVB / narrow-spectrum UVB	 BNGA1/<br>BNGA2/<br>BNGA3	Any time before cohort entry
Therapeutic steroid injection in the joints and soft tissues	BLHN0	Any time before cohort entry
5-ASA/sulfasalazine	A07EC0	1 year before cohort entry
Topical corticosteroids	D07	1 year before cohort entry
Antipsoriatic medication	D05	1 year before cohort entry
Topical calcineurin inhibitors	D11AH01-02,	1 year before cohort entry
Comorbidities		
Cardiovascular disease	DI1-I7	Any time before cohort entry
Pulmonary disease<br>Chronic disease of the lower airways<br>Other interstitial lung disease<br>Diseases with pus and necrosis in the lower airway<br>Interstitial lung emphysema<br>compensatory emphysema	 DJ4<br>DJ84<br>DJ85<br>DJ982<br>DJ983	Any time before cohort entry

For peer review only

Liver disease	DK70-K77	Any time before cohort entry
Kidney disease<br>Glomerular disease<br>Tubulointerstitial kidney disease and kidney insufficiency	 DN0<br>DN1	Any time before cohort entry
Other gastrointestinal diseases<br>Gingivitis and periodontal disease<br>Inflammation of the oral mucosa and related<br>Stomach and duodenal ulcers<br>Fissure and rifts in and around the anus<br>Abscess in and around the anus<br>Other diseases of the rectum and anus<br>Other bowel disease	 DK05<br>DK12<br>DK25-27<br>DK60<br>DK61<br>DK62<br>DK63	Any time before cohort entry
Skin disease<br>Bullous skin disease<br>Dermatitis and eczema<br>Alopecia areata<br>Vitiligo<br>Granulomatous disease in the skin and subcutaneous tissue<br>Lupus<br>Other localised connective tissue disease<br>Vasculitis limited to the skin	 DL10-14<br>DL20-30<br>DL63<br>DL80<br>DL92<br>DL93<br>DL94<br>DL95	Any time before cohort entry
Musculoskeletal disease<br>Generalised connective tissue diseases<br>Diseases of the muscles<br>Soft-tissue rheumatism	 DM30-<br>DM36<br>DM60-63<br>DM70-79	Any time before cohort entry
Medications		
Cardiovascular drugs	MC01-MC10	Any time before cohort entry
Antibiotics	MJ01	Any time before cohort entry
Oral anticoagulants	MB01AA<br>MB01AF	Any time before cohort entry
Drugs used in diabetes	MA10	Any time before cohort entry
Drugs for obstructive airway diseases	MR03	Any time before cohort entry

For peer review only

Supplementary Table 3. Minimum daily dose of corticosteroid in exposed persons

Compound	Equivalent dose
Cortisone	38mg
Hydrocortisone	30mg
Prednisone	7.5mg
Prednisolone	7.5mg
Triamcinolone	6mg
Methylprednisolone	6mg
Betamethasone	0.9mg
Dexamethasone	1.2mg

For peer review only

Supplementary Table 4. Risk of infection, hospitalisation and death associated with immunosuppressive therapy in vaccinated patients with immune-mediated inflammatory diseases

	Exposed				Unexposed				Crude HR (95% CI) ***	Weighted HR (95% CI) ***
	Total	Events	Incidence rate (95% CI) **	PY	Total	Events	Incidence rate (95% CI) **	PY		
**COVID-19 Infection***										
IBD	10 477	287	54.7 (48.7-61.4)	5 246	46 974	885	42.5 (39.8-45.4)	20 809	1.7 (1.5-1.9)	1.6 (1.4-1.9)
Arthropathy	24 255	476	40.3 (36.8-44.1)	11 822	44 650	800	37.8 (35.3-40.6)	21 140	1.3 (1.1-1.4)	1.3 (1.1-1.4)
Psoriasis	5 023	103	45.3 (37.4-55.00)	2 273	20 995	392	41.5 (37.6-45.8)	9 441	1.1 (0.9-1.4)	1.1 (0.9-1.4)
Combined cohorts	39 755	866	-	19 341	112 619	2 077	-	51 390	1.4 (1.3-1.5)	1.4 (1.2-1.5)
**COVID-19 Hospitalization***										
IBD	10 477	15	2.8 (1.7-4.7)	5 280	46 977	43	2.1 (1.5-2.8)	20 892	2.8 (1.5-5.1)	2.1 (1.0-4.1)
Arthropathy	24 255	37	3.1 (2.3-4.3)	11 873	44 650	59	2.8 (2.2-3.6)	21 215	1.3 (0.9-2.0)	1.3 (0.9-2.0)
Psoriasis	5 023	n<5	-	2 284	20 997	20	2.1 (1.4-3.3)	9 479	0.5 (0.1-2.3)	0.6 (0.1-2.5)
Combined cohorts	39 756	52	-	19 437	112 624	122	-	51 594	1.6	1.4

For peer review only

	39 755				112 619				(1.1-2.2)	(1.0-2.0)
**Death***										
IBD	10 477	n<5	-	5 281	46 977	6	0.3 (0.1-0.6)	20 897	1.1 (0.1-9.7)	0.4 (0-5.6)
Arthropathy	24 255	6	0.51 (0.2-1.1)	11 875	44 650	13	0.6 (0.4-1.1)	21 222	1.0 (0.4-2.7)	1.0 (0.4-2.6)
Psoriasis	5 023	0	-	2 284	20 997	n<5	-	9 480	-	-
Combined cohorts	39 756	6	-	19 440	112 624	19	-	51 599	1.0 (0.4-2.5)	0.9 (0.4-2.2)

Abbreviations: CI = confidence interval; HR = hazard ratio; IBD= inflammatory bowel disease; PY = person-years.

*COVID19- Infection = confirmed positive antigen or PCR test result for SARS-CoV2; COVID-19 Hospital Admission = Hospitalisation with SARS-CoV2 infection as primary diagnosis code; Death within 60 days of positive SARS-CoV2 test.

**Events/1000 PY (95% CI)

*** Sex and age adjusted crude and weighted hazard ratios

For peer review only

Supplementary Table 5. Risk of COVID-19 infection associated with immunosuppressive therapy in vaccinated patients with immune-mediated inflammatory diseases at 0-3 months, 3-6 months, and 6-11 months post-vaccination

	**Exposed**				**Unexposed**				**Crude HR (95% CI)** **	**Weighted HR (95% CI)** **
	**Total**	**Events**	**Incidence rate (95% CI)** *	**PY**	**Total**	**Events**	**Incidence rate (95% CI)** *	**PY**		
**COVID-19 infection 0-3 months post-vaccination**										
IBD	10 477	57	22.2 (17.1-28.8)	2 570	46 974	196	17.0 (14.8-19.5)	11 546	1.6 (1.2-2.2)	1.5 (1.1-2.2)
Arthropathy	24 255	96	16.1 (13.2-19.6)	5 978	44 650	166	15.1 (13.0-17.6)	10 990	1.3 (1.0-1.7)	1.3 (1.0-1.7)
Psoriasis	5 023	24	19.4 (13.0-29.0)	1 236	20 995	83	16.1 (13.0-19.9)	5 163	1.3 (0.3-2.1)	1.3 (0.8-2.1)
Combined cohorts	39 755	177	-	4 403	112 619	445	-	27 699	1.4 (1.2-1.7)	1.4 (1.1-1.6)
**COVID-19 infection 3-6 months post-vaccination**										
IBD	10 010	123	45.8 (38.4-54.7)	2 683	45 202	526	56.8 (52.1-61.8)	9 266	0.9 (0.8-1.2)	1.0 (0.8-1.2)
Arthropathy	23 503	284	48.6 (43.2-54.5)	5 849	43 186	482	47.5 (43.4-51.9)	10 149	1.2 (1.1-1.4)	1.2 (1.1-1.4)
Psoriasis	4 860	63	60.7 (47.4-77.7)	1 038	20 203	233	54.5 (47.9-61.9)	4 279	1.1 (0.8-1.4)	1.0 (0.8-1.4)
Combined cohorts	38 373	470	-	19 437	108 591	1 241	-	23 694	1.1 (1.0-1.2)	1.1 (1.0-1.3)

For peer review only

**COVID-19 infection 6-11 months post-vaccination**										
IBD	6 058	107	160.6 (132.9-194.1)	666	15 742	163	107.1 (91.9-124.9)	1 522	1.3 (0.9-1.7)	1.4 (1.1-1.9)
Arthropathy	12 223	96	82.7 (67.7-101.0)	1 160	19 839	152	80.8 (68.9-94.7)	1 881	1.1 (0.8-1.4)	1.1 (0.8-1.4)
Psoriasis	1 719	16	93.8 (57.5-153.1)	170	7 444	76	109.8 (87.7-137.5)	691	0.9 (0.5-1.5)	0.9 (0.5-1.5)
Combined cohorts	20 000	219	-	1 996	43 025	391	-	4 094	1.1 (0.9-1.3)	1.11 (0.9-1.3)

Abbreviations: CI = confidence interval; HR = hazard ratio; IBD= inflammatory bowel disease; PY = person-years.

*Events/1000 PY

**Sex and age adjusted crude and weighted hazard ratios

For peer review only

Supplementary Table 6. Risk of infection, hospitalisation and death associated with immunosuppressive therapy in vaccinated patients with immune-mediated inflammatory diseases by immunosuppressive drug class

	Exposed				Unexposed				Crude HR (95% CI) ***	Weighted HR (95% CI) ***
	Total	Events	PY	Incidence rate (95% CI) **	Total	Events	PY	Incidence rate (95% CI) **		
**COVID-19 Infection***										
Selective Immunosuppressants	3 384	68	1 846	39.4 (31.0-49.9)	151 936	2 079	51 334	40.5 (38.8-42.3)	1.3 (1.0-1.7)	1.3 (1.0-1.6)
Tumour Necrosis Factor Inhibitors	14 277	418	6 908	62.5 (56.7-68.7)	140 797	2 070	51 355	40.3 (38.6-42.1)	1.8 (1.6-2.0)	1.8 (1.6-2.0)
Interleukin Inhibitors	2 781	52	1 385	40.1 (30.6-52.7)	152 584	2 082	51 352	40.5 (38.8-42.3)	1.1 (0.8-1.4)	1.0 (0.8-1.4)
Calcineurin Inhibitors	154	n<5	129	-	155 309	2 073	51 298	40.4 (38.7-42.2)	1.3 (0.3-5.2)	0.5 (0.2-1.5)
Systemic Corticosteroids	5 504	97	2 991	34.6 (28.4-42.3)	149 667	2 079	51 503	40.4 (38.7-42.1)	1.3 (1.0-1.6)	1.2 (1.0-1.5)
Rituximab and Other Immunosuppressants	21 038	426	10 703	41.2 (37.5-45.3)	112 593	2 075	51 409	40.4 (38.7-42.1)	1.3 (1.2-1.4)	1.3 (1.1-1.4)
**COVID-19 Hospitalization***										
Selective Immunosuppressants	3 384	7	1 735	4.0 (1.9-8.5)	112 479	122	51 531	2.4 (2.0-2.8)	3.0 (1.4-6.5)	2.2 (1.0-5.2)
Tumour Necrosis Factor Inhibitors	14 277	16	6 740	2.4 (1.5-3.9)	112 506	116	51 551	2.3 (1.9-2.1)	2.0 (1.2-3.3)	1.8 (1.0-3.3)

For peer review only

Interleukin Inhibitors	2 781	0	1 302	-	112 445	125	51 549	2.4 (2.0-2.9)	-	-
Calcineurin Inhibitors	154	0	83	-	112 404	122	51 493	2.4 (2.0-2.8)	-	-
Systemic Corticosteroids	5 504	18	2 811	6.4 (4.0-10.2)	112 587	122	51 699	2.4 (2.0-2.8)	2.4 (1.4-3.9)	1.8 (1.0-3.0)
Rituximab and Other Immunosuppressants	21 038	25	10 387	2.4 (1.6-3.6)	112 593	121	51 604	2.3 (2.0-2.8)	1.2 (0.5-1.0)	1.1 (0.7-1.8)
**Death***										
Selective Immunosuppressants	3 384	0	1 736	-	112479	23	51 545	0.5 (0.3-0.7)	-	-
Tumour Necrosis Factor Inhibitors	14 277	0	6 741	-	112506	19	5 1564	0.4 (0.2-0.6)	-	-
Interleukin Inhibitors	2 781	0	1 302	-	112 445	24	51 564	0.5 (0.3-0.7)	-	-
Calcineurin Inhibitors	154	0	83	-	112 404	24	51 507	0.5 (0.3-0.7)	-	-
Systemic Corticosteroids	5 504	6	2 812	2.1 (2.0-4.8)	112 587	23	51 713	0.4 (0.3-0.7)	4.0 (1.6-9.9)	2.3 (0.9-6.2)
Rituximab and Other Immunosuppressants	21 038	n<5	10 388	-	112 593	22	51 619	0.4 (0.3-0.7)	0.5 (0.1-2.3)	0.5 (0.1-2.4)

For peer review only

Abbreviations: CI = confidence interval; HR = hazard ratio; IBD= inflammatory bowel disease; PY = person-years.

Immunosuppressive Drug Class: Selective Immunosuppressants, Tumour Necrosis Factor Inhibitors, Interleukin Inhibitors, Calcineurin Inhibitors, Systemic Corticosteroids, and Other Immunosuppressants including Rituximab (See Supplementary for complete ATC codes)

*COVID19- Infection = confirmed positive antigen or PCR test result for SARS-CoV2; COVID-19 Hospital Admission = Hospitalisation with SARS-CoV2 infection as primary diagnosis code; Death within 60 days of positive SARS-CoV2 test.

**Events/1000 PY

*** Sex and age adjusted crude and weighted hazard ratios

For peer review only

STROBE Statement—Checklist of items that should be included in reports of ***cohort studies***

The Impact of Immunosuppressive Therapy on SARS-CoV-2 mRNA Vaccine Effectiveness in Patients with Immune-mediated Inflammatory Diseases: A Nationwide Danish Cohort Study

	Item No	Recommendation	Page No
**Title and abstract**	1	(*a*) Indicate the study's design with a commonly used term in the title or the abstract	3
		(*b*) Provide in the abstract an informative and balanced summary of what was done and what was found	3
**Introduction**			
Background/rationale	2	Explain the scientific background and rationale for the investigation being reported	5
Objectives	3	State specific objectives, including any prespecified hypotheses	5, 6
**Methods**			
Study design	4	Present key elements of study design early in the paper	6-8
Setting	5	Describe the setting, locations, and relevant dates, including periods of recruitment, exposure, follow-up, and data collection	6, 7 & Fig.1
6-10Participants	6	(*a*) Give the eligibility criteria, and the sources and methods of selection of participants. Describe methods of follow-up	6-8 &
		(*b*) For matched studies, give matching criteria and number of exposed and unexposed	Fig.1
Variables	7	Clearly define all outcomes, exposures, predictors, potential confounders, and effect modifiers. Give diagnostic criteria, if applicable	7,8
Data sources/ measurement	8*	For each variable of interest, give sources of data and details of methods of assessment (measurement). Describe comparability of assessment methods if there is more than one group	7,8
Bias	9	Describe any efforts to address potential sources of bias	6-9
Study size	10	Explain how the study size was arrived at	Fig. 1
Quantitative variables	11	Explain how quantitative variables were handled in the analyses. If applicable, describe which groupings were chosen and why	7-9
Statistical methods	12	(*a*) Describe all statistical methods, including those used to control for confounding<br>(*b*) Describe any methods used to examine subgroups and interactions<br>(*c*) Explain how missing data were addressed<br>(*d*) If applicable, explain how loss to follow-up was addressed<br>(*e*) Describe any sensitivity analyses	7-9
**Results**			
Participants	13*	(a) Report numbers of individuals at each stage of study—eg numbers potentially eligible, examined for eligibility, confirmed eligible, included in the study, completing follow-up, and analysed<br>(b) Give reasons for non-participation at each stage<br>(c) Consider use of a flow diagram	9,10 & Table 1
Descriptive data	14*	(a) Give characteristics of study participants (eg demographic, clinical, social) and information on exposures and potential confounders	9,10

For peer review only - http://bmjopen.bmj.com/site/about/guidelines.xhtml

		(b) Indicate number of participants with missing data for each variable of interest<br>(c) Summarise follow-up time (eg, average and total amount)	
Outcome data	15*	Report numbers of outcome events or summary measures over time	9 Fig.2-4
Main results	16	(*a*) Give unadjusted estimates and, if applicable, confounder-adjusted estimates and their precision (eg, 95% confidence interval). Make clear which confounders were adjusted for and why they were included<br>(*b*) Report category boundaries when continuous variables were categorized<br>(*c*) If relevant, consider translating estimates of relative risk into absolute risk for a meaningful period	9,10 & Fig. 2-4
Other analyses	17	Report other analyses done—e.g. analyses of subgroups and interactions, and sensitivity analyses	10 Fig.4
**Discussion**			
Key results	18	Summarise key results with reference to study objectives	10
Limitations	19	Discuss limitations of the study, taking into account sources of potential bias or imprecision. Discuss both direction and magnitude of any potential bias	13
Interpretation	20	Give a cautious overall interpretation of results considering objectives, limitations, multiplicity of analyses, results from similar studies, and other relevant evidence	13
Generalisability	21	Discuss the generalisability (external validity) of the study results	11,12
**Other information**			
Funding	22	Give the source of funding and the role of the funders for the present study and, if applicable, for the original study on which the present article is based	Abstract & 14

*Give information separately for exposed and unexposed groups.

Note: An Explanation and Elaboration article discusses each checklist item and gives methodological background and published examples of transparent reporting. The STROBE checklist is best used in conjunction with this article (freely available on the Web sites of PLoS Medicine at http://www.plosmedicine.org/, Annals of Internal Medicine at http://www.annals.org/, and Epidemiology at http://www.epidem.com/). Information on the STROBE Initiative is available at http://www.strobe-statement.org.

For peer review only

BMJ Open

The Impact of Immunosuppressive Therapy on SARS-CoV-2 mRNA Vaccine Effectiveness in Patients with Immune-mediated Inflammatory Diseases: A Danish Nationwide Cohort Study

Journal:	*BMJ Open*
Manuscript ID	bmjopen-2023-077408.R1
Article Type:	Original research
Date Submitted by the Author:	21-Dec-2023
Complete List of Authors:	Elmahdi, Rahma; Aalborg Universitet, Department of Clinical Medicine ; Aalborg University Hospital, Department of Gastroenterology and Hepatology<br>Ward, Daniel; Aalborg Universitet, Department of Clinical Medicine<br>Ernst, Martin; University of Southern Denmark, Clinical Pharmacology and Pharmacy, Department of Public Health<br>Poulsen, Gry; Aalborg University, Department of Clinical Medicine<br>Hallas, Jesper; University of Southern Denmark, Clinical pharmacology and pharmacy<br>Pottegard, Anton; University of Southern Denmark, Clinical Pharmacology and Pharmacy, Department of Public Health<br>Jess, Tine; Aalborg Universitet, Department of Clinical Medicine; Aalborg University Hospital, Department of Gastroenterology and Hepatology
**Primary Subject Heading**:	Epidemiology
Secondary Subject Heading:	Gastroenterology and hepatology, Infectious diseases
Keywords:	COVID-19, EPIDEMIOLOGY, Inflammatory bowel disease < GASTROENTEROLOGY, RHEUMATOLOGY, Psoriasis < DERMATOLOGY

SCHOLARONE™
Manuscripts

I, the Submitting Author has the right to grant and does grant on behalf of all authors of the Work (as defined in the below author licence), an exclusive licence and/or a non-exclusive licence for contributions from authors who are: i) UK Crown employees; ii) where BMJ has agreed a CC-BY licence shall apply, and/or iii) in accordance with the terms applicable for US Federal Government officers or employees acting as part of their official duties; on a worldwide, perpetual, irrevocable, royalty-free basis to BMJ Publishing Group Ltd ("BMJ") its licensees and where the relevant Journal is co-owned by BMJ to the co-owners of the Journal, to publish the Work in this journal and any other BMJ products and to exploit all rights, as set out in our licence.

The Submitting Author accepts and understands that any supply made under these terms is made by BMJ to the Submitting Author unless you are acting as an employee on behalf of your employer or a postgraduate student of an affiliated institution which is paying any applicable article publishing charge ("APC") for Open Access articles. Where the Submitting Author wishes to make the Work available on an Open Access basis (and intends to pay the relevant APC), the terms of reuse of such Open Access shall be governed by a Creative Commons licence – details of these licences and which Creative Commons licence will apply to this Work are set out in our licence referred to above.

Other than as permitted in any relevant BMJ Author's Self Archiving Policies, I confirm this Work has not been accepted for publication elsewhere, is not being considered for publication elsewhere and does not duplicate material already published. I confirm all authors consent to publication of this Work and authorise the granting of this licence.

For peer review only

The Impact of Immunosuppressive Therapy on SARS-CoV-2 mRNA Vaccine Effectiveness in Patients with Immune-mediated Inflammatory Diseases: A Danish Nationwide Cohort Study

Short Title: COVID-19 Vaccination in Immunosuppressed IMID Patients

Rahma Elmahdi*[1,2] (0000-0002-7013-9548), Daniel Ward*[1] (0000-0003-0405-7250), Martin Thomsen Ernst[3] (0000-0001-9003-3823), Gry Juul Poulsen[1] (0000-0003-2744-2469), Jesper Hallas[3] (0000-0002-8097-8708), Anton Pottegård[3] (0000-0001-9314-5679), Tine Jess[1,2] (0000-0002-4391-7332)

* Co-first author

Rahma Elmahdi, Clinical Research Associate, Center for the Molecular Prediction of Inflammatory Bowel Disease (PREDICT), Department of Clinical Medicine, Aalborg University, Denmark (1) Department of Gastroenterology and Hepatology, Aalborg University Hospital, Aalborg, Denmark (2)

Daniel Ward, Doctoral Fellow, Center for the Molecular Prediction of Inflammatory Bowel Disease (PREDICT), Department of Clinical Medicine, Aalborg University, Denmark (1)

Martin Ernst Thomsen, Data Manager, Department of Public Health, Clinical Pharmacology, Pharmacy and Environmental Medicine, University of Southern Denmark, J.B. Winsløws Vej 19, 5000 Odense, Denmark (3)

Gry Juul Poulsen, Statistician, Center for the Molecular Prediction of Inflammatory Bowel Disease (PREDICT), Department of Clinical Medicine, Aalborg University, Denmark (1)

Jesper Hallas, Professor, Research Unit of Clinical Pharmacology, University of Southern Denmark, J.B. Winsløws Vej 9, 5000 Odense, Denmark (3)

Anton Pottegård, Professor, Department of Clinical Pharmacology and Pharmacy, University of Southern Denmark, J.B. Winsløws Vej 9, 5000 Odense, Denmark (3)

For peer review only - http://bmjopen.bmj.com/site/about/guidelines.xhtml

Tine Jess, Professor, Center for the Molecular Prediction of Inflammatory Bowel Disease (PREDICT), Department of Clinical Medicine, Aalborg University, Denmark (1)

Professor, Department of Gastroenterology and Hepatology, Aalborg University Hospital, Aalborg, Denmark (2)

Correspondence: Dr Rahma Elmahdi, National Center of Excellence for the Molecular Prediction of Inflammatory Bowel Disease, PREDICT, Aalborg University, A. C. Meyers Vænge 15, DK-2450 Copenhagen, Denmark. E-mail: rahmae@dcm.aau.dk

Key words: Immune-mediated inflammatory disease; Cohort study; SARS-CoV-2; Vaccination; Immunosuppressives

Word count: Abstract (excluding Strengths and limitations of this study), 283; manuscript body (excluding tables, figure legends and references), 3840

For peer review only - http://bmjopen.bmj.com/site/about/guidelines.xhtml

ABSTRACT

Objective: Patients receiving immunosuppressives have been excluded from trials for SARS-CoV-2 vaccine efficacy. Investigation of immunosuppressants' impact on effectiveness of vaccines, particularly in patients with immune-mediated inflammatory diseases (IMID), are therefore required.

Design: We performed a nationwide cohort study to assess the risk of COVID-19 infection in vaccinated IMID patients exposed to immunosuppressives compared to IMID unexposed to immunosuppressives. Exposure to immunosuppressives in the 120 days before receiving the second SARS-CoV-2 mRNA vaccination was assessed. Patients were followed from date of second vaccination, and weighted Cox models were used to estimate the risk of infection associated with immunosuppressives. Secondary outcomes included hospitalization and death associated with a positive SARS-CoV-2 test. Risk of infection by immunosuppressant drug class was also analysed.

Setting: This study used population-representative data from Danish national health registries in the period from 1st January to 30th November 2021.

Results: Overall, 152,440 patients were followed over 19,341 person-years. Immunosuppressants were associated with a significantly increased risk of infection overall (HR: 1.4, 95% CI: 1.2, 1.5), in inflammatory bowel disease (IBD) (HR: 1.6, 95% CI: 1.4, 1.9) and arthropathy (HR:1.3, 95% CI: 1.1, 1.4) but not psoriasis (HR: 1.1, 95% CI: 0.88, 1.4). Immunosuppressants were also associated with an increased risk of hospitalization overall (HR: 1.4, 95% CI: 1.0, 2.0), particularly in IBD (HR: 2.1, 95% CI: 1.0, 4.1). No significantly increased risk of death in immunosuppressants exposed patients was identified. Analyses by immunosuppressant drug class showed increased COVID-19

For peer review only

infection and hospitalization with anti-TNF, systemic corticosteroid, and rituximab and other immunosuppressants.

Conclusion: Immunosuppressive therapies reduced effectiveness of mRNA SARS-CoV-2 vaccination against infection and hospitalization in IMID patients. Anti-TNF, systemic corticosteroids, and rituximab and other immunosuppressants were particularly associated with these risks.

Strengths and limitations of this study

- Use of a non-selected, population representative cohort to source inflammatory and immune mediated disease (IMID) patients.
- Inclusion of a total of 184,346 immunosuppressive exposed IMID patients and 152,440 propensity score unexposed matched controls.
- Complete vaccination, and immunosuppressive treatment exposure data along with complete infection, hospitalization, and death outcome data with no loss to follow-up.
- Lack of individual level data on level of exposure to infection, including shielding behaviour.

For peer review only - http://bmjopen.bmj.com/site/about/guidelines.xhtml

INTRODUCTION

SARS-CoV-2 mRNA vaccines Comirnaty® (Pfizer-BioNTech) and Spikevax® (Moderna) were found to be efficacious in clinical trials prior to authorisation, and by December 2022 over 758 million doses of Pfizer-BioNTech and 164 million doses of Moderna were administered in the European Union [1]. Pre-marketing trials excluded individuals considered at risk of immunocompromise, including those receiving immunosuppressive therapies [2,3], therefore there remains a paucity of data on the real-world effectiveness of SARS-CoV-2 vaccines in patients treated with immunosuppressive drugs.

Research published early in the SARS-CoV-2 epidemic suggested that, in the absence of vaccination, some types of immunosuppressives including rituximab, sulfasalazine, and corticosteroids are associated with an increased risk of severe outcomes in COVID-19 infection [4–8]. Immune-mediated inflammatory diseases (IMID), including inflammatory bowel disease, inflammatory arthropathy, and psoriasis have themselves independently been associated with lower serological responses to SARS-CoV-2 vaccination than in healthy controls [9]. Immunosuppressants are key therapies in IMID, so patients with IMID may be at increased risk of infection and severe outcomes of COVID-19 infection both due to the natural history of the diseases and the therapies used to treat them. Even in the context of second vaccination against SARS-CoV-2, exposure to immunosuppressives has been associated with a significantly poorer humoral response; lower than that which is required to confer immunity against infection and severe outcomes of COVID-19 infection in patients treated with immunosuppressive therapies [10–12].

For peer review only - http://bmjopen.bmj.com/site/about/guidelines.xhtml

It is therefore important to investigate the impact of immunosuppressants on SARS-CoV-2 vaccine effectiveness, while controlling for the underlying disease-indicating treatment, and other confounders that may impact vaccine effectiveness.

The real-world effectiveness of SARS-CoV-2 mRNA vaccinations against COVID-19 infection and associated outcomes such as hospitalization or death, in the context of immunosuppressive therapy exposure among IMID patients has not yet been investigated. The aim of this study was to use Danish nationwide population-based data to assess the impact of immunosuppressive exposure on the risk of COVID-19 infection in three cohorts of vaccinated IMID patients.

MATERIALS AND METHODS

Data sources

We conducted a nationwide cohort study using the Danish COVID-19 cohort [13], based on data from the Danish Microbiology Registry [14], which includes individual-level information on vaccine type, dose, and date of administration; SARS-CoV-2 test type and date administered. This data was linked at the individual level to both the Danish National Patient Registry [15]and the Danish National Prescription Registry [16] using a unique Danish Civil Registration number (assigned to all individuals residing in Denmark). The Danish National Patient Registry, a register of hospital activities, includes medical diagnoses coded using International Classification of Disease (ICD-10), and medical procedures and prescriptions including treatment with intravenous medications. The Danish National Prescription Registry contains information on prescriptions dispensed at all community retail pharmacies, including date of dispensing, tablet strength, and pack sizes. Ethics board review is not required for epidemiological research using nationwide registers in Denmark as data is pseudonymised and does not involve patients.

For peer review only - http://bmjopen.bmj.com/site/about/guidelines.xhtml

Population, follow-up, and outcomes

The IMID cohort comprised of three disease specific cohorts of all patients diagnosed with inflammatory bowel disease, including Crohn's disease and ulcerative colitis (IBD; ICD-10: K50, K51), inflammatory arthropathy, including ankylosing spondylitis, other inflammatory spondylopathies, seropositive rheumatoid arthritis, other rheumatoid arthritis, and psoriatic and enteropathic arthropathies (ICD-10: M45, M46, M05, M06, M07) or psoriasis (ICD-10: L40) in Denmark, who had received two doses of SARS-CoV-2 mRNA (Pfizer-BioNTech or Moderna) vaccine. Exclusion criteria were not receiving two doses of SARS-CoV-2 mRNA vaccine and migration prior to receipt of second vaccination. Patients with more than one of these IMID diagnoses were included in only one cohort, with IBD taking precedence, then inflammatory arthropathy, finally psoriasis. Therefore, only patients with psoriasis and neither an IBD nor an inflammatory arthropathy diagnosis were included in the psoriasis cohort. This order was preferred as extent of organ-specific disease likely determines the dose for immunosuppressive therapy. Registration of IMIDs is based on clinical diagnoses, in line with national and international guidelines, such as ECCO-ESGAR guidelines for IBD diagnosis [17,18].

Patients were followed from the date of administration of second mRNA vaccine (the index date) after 1 January 2021. The primary outcome was a positive SARS-CoV-2 PCR test in the observation period. Secondary outcomes were hospitalization between 7 days prior to and up to 28 days after a positive test or death within 60 days of a positive test (both recorded in Danish National Patient Register). Follow-up was censored at administration of a third vaccination, emigration, death (in the absence of a positive SARS-CoV-2 test) or the end of the study period, 30 November 2021 (Figure 1), as prevalence of the omicron variant became substantial after 28 November 2021 [19]. As the

For peer review only - http://bmjopen.bmj.com/site/about/guidelines.xhtml

registers are complete for the presence of patients up to emigration or death, therefore all patients are retained until the event and there is no missing data.

Patient and Public Involvement

None.

Exposures

The exposures for this study include dispensed prescriptions or hospital administration of an immunosuppressive in the 120 days preceding the index date (date of administration of second vaccination). Immunosuppressants included selective immunosuppressants, anti-TNFs, interleukin inhibitors, calcineurin inhibitors, corticosteroids, rituximab and other immunosuppressants (see Supplementary Table 1 for complete list and ACT codes for immunosuppressants). The 120-day exposure window is chosen to cover the largest pack sizes of prescriptions which can contain medications for up to 120 days. A minimum daily dose of corticosteroids equivalent to 7.5 mg prednisolone per day was estimated as the entire dispensed quantity of corticosteroids during a sequence of prescriptions (within the 120-day exposure period) divided by the number of days from the first prescription to the index date (Supplementary Table 2). Unexposed IMID patients were defined as those with a diagnosis of one of the three IMID, who had not received an immunosuppressive in the 120 days preceding the index date, and those receiving <7.5 mg prednisolone-equivalent average per day.

Statistical models

Covariates included age (handled as a continuous covariate), sex, comorbidities (cardiovascular disease, pulmonary disease, liver disease, kidney disease, other gastrointestinal diseases, skin disease,

For peer review only - http://bmjopen.bmj.com/site/about/guidelines.xhtml

and musculoskeletal disease), medications (cardiovascular drugs, antibiotics, oral anticoagulants, diabetes, and chronic airway disease medications). Testing frequency varied during the period studied due to changes in national and international guidelines and travel restrictions, along with the background prevalence of SARS-CoV-2, which could introduce bias in case detection. We therefore adjusted for individual testing frequency by including number of tests in the month preceding index date as a continuous covariate. Further, covariates specific to each IMID were included separately for each cohort. For the IBD cohort this included any IBD-related hospital admissions in the previous year, Crohn's disease, ulcerative colitis, 5-ASA/sulfasalazine, budesonide, IBD-related procedures, and endoscopy of the gastrointestinal tract (see Supplementary Table 3 for complete list of IMID cohort specific covariates).

To balance the covariates in the exposed and unexposed groups, we fitted propensity score (PS) models for each IMID cohort separately. Propensity scores were calculated using logistic regression for the probability of exposure (treatment with immunosuppressives) conditional on the covariates defined above [20]. We subsequently implemented the PS using standardized mortality ratio (SMR) weights (with trimming of subjects with extreme weights beyond 1st and 99th centiles). We assessed the distribution of covariates with standardized differences before and after PS weighting.

We used weighted Cox proportional-hazards regression models [21] to estimate risk of the COVID-19 infection in IMID patients exposed to immunosuppressive therapy compared to unexposed patients for each disease cohort separately. We used calendar time as the underlying time scale to account for period effects on the risk of the outcomes which may relate to varying infection prevalence and patient characteristics as patients vulnerable to severe outcomes were vaccinated earlier in the year.

For peer review only

For peer review only - http://bmjopen.bmj.com/site/about/guidelines.xhtml

We performed secondary analyses to further delineate the impact of immunosuppressives on vaccine effectiveness over time by stratifying time since vaccination into the following intervals: 0-3 months, 3-6 months, 6-11 months. This not only allowed us to capture the period effects of COVID-19 infection risk earlier and later in the pandemic period but also allowed us to assess the impact of censoring at different time points in the follow-up period.

We then undertook Fixed Effects Model (FEM) meta-analysis to calculate the pooled HR of infection, hospitalization, and death for IBD, arthropathy and psoriasis cohorts as overall risk in immunosuppressive exposed IMID by COVID-19 outcome, and the HR of infection during 0-3, 3-6, and 6-11 months of follow-up period, as overall risk in immunosuppressive exposed IMID by period.

Finally, we also undertook drug specific analysis for risk of COVID-19 infection by immunosuppressive drug class. In this analysis, patients receiving multiple immunosuppressive treatments were treated as independently exposed to each drug class. To account for the potential impact of immunosuppressants commonly prescribed in a weaning dose, which would not be captured using the definition of $\geq$7.5 mg dose equivalent per day, we undertook a sensitivity analysis to assess whether having any prescription for systemic corticosteroids over the 120-day period before the index date had an impact on the risk of infection, hospitalization, or death for those exposed to this class of immunosuppressants.

RESULTS

A total of 184,346 patients diagnosed with IBD, arthropathy or psoriasis were identified. After exclusion of patients not receiving two doses of SARS-CoV-2 mRNA vaccine, migration prior to receipt of second vaccination, and trimming of those with extreme propensity scores a total 152,440

For peer review only - http://bmjopen.bmj.com/site/about/guidelines.xhtml

patients were included, contributing a total 19,341 person-years of follow-up. During the 120-day exposure assessment period, 39,765 IMID patients received immunosuppressive treatment (10,480 IBD, 24,261 arthropathy, and 5,024 psoriasis), and 112,675 IMID patients (47,001 IBD, 44,669 arthropathy, and 21,005 psoriasis) patients did not. A total of 11 exposed and 51 unexposed IMID patients are censored from overall analysis due to migration or inclusion on the date of study end (therefore contributing no follow-up time). One arthropathy patient in the exposed group, and 5 IBD or psoriasis patients in the unexposed group are excluded from the infection analysis due to positive test on the date of study entry, these are subsequently included in the analysis for risk of hospitalization or death following COVID-19 infection (Figure 1; Supplementary Table 4). Following application of SMR weighting, the cohorts were balanced on the included covariates (see Table 1 for covariate prevalence and standardised differences [SD]).

A total of 866 (2.2%) COVID-19 infections were recorded among immunosuppressive exposed IMID patients during the follow-up period compared with 2,077 (1.8%) for unexposed IMID patients. This gave an incidence rate of 55 (49-61) per 1,000 person-years in immunosuppressive exposed IBD patients compared with 43 (40-45) in unexposed IBD patients, 40 (37-44) per 1,000 person-years for immunosuppressive exposed arthropathy patients compared with 38 (35-41) in unexposed arthropathy patients, and 45 (37-55) per 1,000 person-years for immunosuppressive exposed psoriasis patients compared with 42 (38-46) per 1,000 person-years in unexposed psoriasis patients.

A significantly increased weighted hazard for infection among exposed patients was seen for both IBD (HR: 1.6, 95% CI: 1.4, 1.9) and arthropathy (HR: 1.3, 95% CI: 1.1, 1.4) cohorts but not for the psoriasis cohort (HR: 1.1, 95 % CI: 0.88, 1.4). Meta-analysis of the three IMID cohorts showed a pooled HR for COVID-19 infection in exposed patients of 1.4 (95% CI: 1.2, 1.5; Figure 2). Fewer than 52 exposed and 122 unexposed IMID patients were hospitalized with COVID-19 infection

For peer review only - http://bmjopen.bmj.com/site/about/guidelines.xhtml

during the follow-up period, which corresponded to a significantly increased risk of hospitalization overall for immunosuppressive exposed IMID patients (pooled HR: 1.4, 95% CI: 1.0, 2.0). This increased risk of overall hospitalization is largely due to the contribution of the risk of hospitalization in IBD patients 2.05 (95% CI: 1.03-4.07). Less than five immunosuppressive exposed IBD patients died in the 60 days following a COVID-19 diagnosis compared to six unexposed IBD patients. Six patients in the arthropathy cohort exposed to immunosuppressives compared with 13 unexposed arthropathy patients died in the 60 days following a COVID-19 diagnosis and fewer than five patients with a psoriasis diagnosis, either immunosuppressive exposed or unexposed died with a COVID-19 diagnosis. These did not correspond to a significantly increased risk of death among exposed patients in any of the three cohorts or overall (pooled HR: 0.92, 95% CI: 0.38, 2.2; Figure 2).

In the first 0-3 months following vaccination, both IBD and arthropathy immunosuppressive exposed patients had a significantly increased risk of COVID-19 infection (HR: 1.5, 95% CI: 1.1, 2.2 and HR: 1.3, 95% CI: 1.0, 1.7, respectively; see Supplementary Table 5). Most COVID-19 infections following second vaccination occurred in the 3–6-month period with a total of 470 infections in exposed IMID patients compared with 1,277 unexposed IMID patients. Only exposed arthropathy patients had a significantly increased risk of infection compared to their unexposed counterparts during this period however (HR: 1.2, 95% CI: 1.1, 1.4). The highest incidence rate of COVID-19 infection following second vaccination was seen in the 6–11-month period for both immunosuppressive exposed and unexposed IMID patients and risk of infection during this period was only increased among exposed IBD patients (HR: 1.4, 95% CI: 1.1, 1.9). There was however a high rate of censoring among both the immunosuppressive exposed (over 50%) and the unexposed (almost 49%) groups in the 6–11-month period due to receipt of the third SARS-CoV-2 vaccination so direct comparison of the risk of infection between time periods is challenging. Kaplan-Meier plots

For peer review only

and HR showing probability of infection over the calendar time of follow-up (January-November 2021) are presented in Supplementary Figure 1 and post-hoc analysis for HR for infection by calendar period (January-November 2021) is shown in Supplementary Table 6.

Analysis for risk of infection among IMID cohorts by immunosuppressive drug class exposure showed a significantly increased risk of infection among users of anti-TNF (HR: 1.8, 95% CI: 1.6, 2.0), systemic corticosteroid (HR: 1.2, 95% CI: 1.0,1.5), and rituximab and other immunosuppressant (HR: 1.3, 95% CI: 1.1, 1.4; Figure 3). No other immunosuppressant was significantly associated with COVID-19 infection following second vaccination. Anti-TNF and systemic corticosteroid exposure were also associated with an increased risk of COVID-19 associated hospitalization (HR 1.8, 95% CI 1.0, 3.3 and HR 1.8, 95% CI 1.0,3.0, respectively; Figure 4). No immunosuppressive drug class was associated with death among IMID patients following receipt of second vaccination (Supplementary Table 7). Sensitivity analysis assessing outcomes following any systemic corticosteroid exposure in the 120-day period prior to the index date showed no significant difference in risk of infection (crude HR: 1.2, 95% CI: 1.0, 1.4; adjusted HR: 1.1, 95% CI: 0.95, 1.3) or death (crude HR: 3.1, 95% CI: 1.4, 7.0; adjusted HR: 1.9, 95% CI: 0.77, 4.7). However, risk of hospitalization following infection was significantly increased in those ever exposed to systemic corticosteroids in the 120-day period prior to receipt of second vaccination (crude HR: 2.8, 95% CI: 1.9, 4.1; adjusted HR: 2.1, 95% CI: 1.4, 3.2), showing similar results compared with analysis restricting to a ≥7.5 mg daily equivalent dose.

DISCUSSION

In this large nation-wide cohort study of three IMID patient cohorts, we identified a total of 39,756 immunosuppressive exposed patients matched to 112,629 immunosuppressive unexposed IMID

For peer review only - http://bmjopen.bmj.com/site/about/guidelines.xhtml

patients to investigate the risk of COVID-19 infection, hospitalization, and death among IBD, arthropathy and psoriasis patients following second SARS-CoV-2 vaccination. Meta-analysis of the three cohorts showed an overall 35% increased risk of infection and, 42% increased risk of COVID-19 associated hospitalization in immunosuppressive exposed compared to immunosuppressive unexposed IMID patients.

Mortality was not significantly increased in immunosuppressive exposed patients as these events were rare. Drug class analysis showed anti-TNF, systemic corticosteroid, and rituximab and other immunosuppressant exposure was significantly associated with both increased risk of COVID-19 infection and hospitalization following second vaccination in immunosuppressive exposed compared to unexposed IMID patients.

We attempted to ascertain the effectiveness of SARS-CoV-2 mRNA vaccination among IMID patients exposed to immunosuppressive therapies, while controlling for the severity of the underlying disease indicating immunosuppressive treatment with a propensity score model. We found that immunosuppressants were associated with an increased risk of infection, likely due to the impact of immunosuppressive medication on vaccination against COVID-19 infection. This is particularly seen in IBD (HR:1.6, 95% CI: 1.4-1.9) but is also present in arthropathy (HR: 1.3, 95% CI:1.1, 1.4) patients and, to a lesser extent, in psoriasis (HR: 1.1, 95% CI: 0.88, 1.4). Immunosuppressive exposed psoriasis patients showed no increased infection risk compared to their unexposed counterparts. Similarly, when assessing risk of hospitalization following vaccination by immunosuppressive exposure, we find a significantly increased risk in IBD patients (HR: 2.1, 95% CI 1.0,4,1), which is not observed in arthropathy (HR: 1.3, 95% CI: 0.9, 2.0) or psoriasis (HR: 0.6, 95% CI: 0.1, 2.5). The poorer outcomes observed in immunosuppressive exposed IBD patients is in keeping with wider

For peer review only

findings. In a meta-analysis of serological response to SARS-CoV-2 vaccination among IMID treated patients, IBD patients were found to have a lower response to first mRNA vaccination dose than rheumatoid patients (response rate: 0.49, 95% CI: 0.32, 0.66 and 0.78, 95% CI: 0.67, 0.86, respectively) [22]. This may be due to the more extensive disease seen in typical IBD patients, which often necessitates higher doses of immunosuppressive therapies, over longer periods to achieve disease remission than that required for psoriasis or arthropathies [23–25]. However, the difference in increased risk of infection and hospitalization in immunosuppressive exposed IBD compared with unexposed IBD patients is like the other IMID cohorts in this study, with overall pooled IMID cohort meta-analysis showing a significantly increased risk for both these outcomes. These findings indicate a general trend towards poorer outcomes in immunosuppressive exposed patients regardless of IMID cohort.

Reassuringly, immunosuppressant exposure was not associated with increased risk of death due to COVID-19 in any of the IMID patient cohorts, suggesting immunosuppressants do not reduce the effectiveness of vaccination in preventing this important outcome. However, caution should be exercised in the interpretation of this finding as deaths were recorded in either immunosuppressive exposed or unexposed patients and this may be due to the relatively short follow-up period of 11-months in this study. Although Kaplan-Meier plots for risk of infection may appear in contradiction to the overall findings of the primary analysis (with apparent increased rate of COVID infection in the unexposed IMID population in the first 7 months of follow-up), the findings from post-hoc Cox regression analysis by calendar period shows that the difference between the groups, reflected in the overall HR, only becomes apparent in the final 3 months of follow-up as the majority of cases of COVID are seen in this period.

For peer review only

For peer review only - http://bmjopen.bmj.com/site/about/guidelines.xhtml

Meta-analysis showed an overall increased risk of COVID-19 infection among exposed IMID patients during the 0-3 and 3-6-month period only. Only exposed arthropathy patients showed a significantly increased risk of infection in the 3–6-month period. Although this might be interpreted as waning immunity, it is important to note that half of the baseline IMID cohort were censored, largely due to receipt of third vaccination, therefore the remaining population likely differed substantially from the initial cohort. Hence this interpretation of period specific risk estimates should be made tentatively [26]. Although the three periods are not directly comparable, it is likely that our observation of an increased risk of infection in exposed patients in the 0-3- and 3-6-month period reflects a true risk, as is observed in the risk identified over the total follow-up period.

Treatment with TNF-alpha inhibitors, systemic corticosteroid, and rituximab other immunosuppressants was associated with a significantly increased risk of infection, and TNF-alpha inhibitors and systemic corticosteroids were associated with a significantly increased risk of hospitalization following receipt of second vaccination. This is consistent with previous studies, which suggests that treatment with cytokine inhibitors or B-cell depleting immunosuppressives is related to particularly poor COVID-19 outcomes [27–29], however the association with TNF-alpha inhibitors is novel. Our findings of increased risk of infection and hospitalization, but not death, in sensitivity analysis among IMID patients exposed to systemic corticosteroids is also in keeping with those other studies of unvaccinated IMID cohorts in Denmark [4] and internationally [6,30]. These findings indicate that corticosteroid exposure weakens the protection conferred by vaccination. As glucocorticoids are known to inhibit the breadth of the immune response, including aspects of both the cellular and humoral immunity induced by mRNA vaccination, these findings appear to be intuitive [31]. The interaction of IMID, the impact of treatments to control disease and response to vaccination, particularly considering the effects of dose and duration of administration is however

For peer review only

For peer review only - http://bmjopen.bmj.com/site/about/guidelines.xhtml

complex [32,33]. Further studies directly exploring the effects of vaccination whilst controlling for disease severity and exposure of immunosuppressive drugs by dose and duration would be required to disentangle the association of the different immunosuppressive drug classes with COVID-19 outcomes following vaccination. Such studies would also better inform guidance relating to timelines for SARS-CoV-2 vaccination in relation to the administration of immunosuppressive therapies in IMID patients.

One of the key strengths of this study is that it is large and population-representative, exploring the effectiveness of COVID-19 infection using real-world data from comprehensive, nationwide health registries. Vaccination does not directly correlate with protection from infection and the findings from this work provides important evidence on effectiveness of post-marketing mRNA vaccination in a vulnerable patient group. To our knowledge, this is the first study to assess the effectiveness of SARS-CoV-2 vaccination against COVID-19 infection, hospitalization, and mortality among IMID patients, based on immunosuppressive exposure. Additionally, our use of PS weighted regression models allows us to accurately control for the underlying treatment indicating disease, so we are better able to extrapolate the effects of the drug exposure from the disease itself.

Limitations include not being able to extrapolate in the context of the omicron variant, or subsequent subvariants as we restricted to a period of the pandemic where the delta variant was the dominant circulating strain of COVID-19 to ensure consistency in the assessment of our outcomes. Although it is difficult to define a reliable threshold for which we consider a patient unexposed to immunosuppressive medication, it is reassuring that only approximately 12% of our unexposed group had filled a prescription in the 365 days prior to the 120-day exposure window assessed, and that removing the minimum threshold of 7.5 mg of systemic corticosteroids as an exposure requirement

For peer review only - http://bmjopen.bmj.com/site/about/guidelines.xhtml

did not change our findings. A lack of individual-level data relating to confounders such as smoking behaviour, risk of occupational exposure to COVID-19, socio-economic status and dose of drug therapies could potentially limit our findings. Due to lack of availability to such data, we could not account of shielding behaviour in this analysis. There may also be a residual effect of confounding due to unmeasured disease severity not completely accounted for in our PS model. However, these are unlikely to systemically impact the direction of association or strength of significance identified in the risk of infection due to immunosuppressive exposure observed here. We limited our study to IBD, inflammatory arthropathy, and psoriasis although other IMIDs exist, because these are commonly treated with immunosuppressives such as anti-TNF.

In conclusion, our findings suggest a reduced effectiveness of mRNA SARS-CoV-2 vaccination against COVID-19 infection and hospitalization in IMID patients receiving immunosuppressive therapies. This risk is particularly seen in IBD and arthropathy patients, and COVID-19 infection is associated with anti-TNF, systemic corticosteroids, and rituximab and other immunosuppressant exposure, while TNF-alpha inhibitors and systemic corticosteroids were associated with a significantly increased risk of hospitalization.

Author Contributions: DW, RE, AP and TJ developed the study protocol. ME undertook primary data analysis with support from GP. DW and RE were responsible for first draft of the manuscript. All authors were responsible for interpretation of results and critical revisions to the final manuscript.

Funding Sources: This work was funded by grants from the Novo Nordisk Foundation (NNF21OC0068631) and the Danish National Research Foundation (DNRF-148).

For peer review only - http://bmjopen.bmj.com/site/about/guidelines.xhtml

Conflict of Interest Statement: All authors have none to declare.

Data Sharing Statement: The study was based on data from the Danish National Health registers (https://sundhedsdatastyrelsen.dk). The register data are protected by the Danish Act on Processing of Personal Data and are accessed through application to and approval from the Danish Data Protection Agency and the Danish Health Data Authority. The code is available promptly on request made to the corresponding author

For peer review only

For peer review only - http://bmjopen.bmj.com/site/about/guidelines.xhtml

REFERENCES

1 European Medicines Agency. COVID-19 vaccines safety update: 3 August 2022 Rev. 1. Amsterdam: www.ema.europa.eu (accessed 11 Aug 2022).

2 El Sahly HM, Baden LR, Essink B, *et al.* Efficacy of the mRNA-1273 SARS-CoV-2 Vaccine at Completion of Blinded Phase. *N Engl J Med* 2021;**385**:1774–85. doi:10.1056/nejmoa2113017

3 Polack FP, Thomas SJ, Kitchin N, *et al.* Safety and Efficacy of the BNT162b2 mRNA Covid-19 Vaccine. *N Engl J Med* 2020;**383**:2603–15. doi:10.1056/NEJMOA2034577/SUPPL_FILE/NEJMOA2034577_PROTOCOL.PDF

4 Ward D, Gørtz S, Ernst MT, *et al.* The effect of immunosuppressants on the prognosis of SARS-CoV-2 infection. *Eur Respir J* 2022;**59**. doi:10.1183/13993003.00769-2021

5 Gianfrancesco M, Hyrich KL, Al-Adely S, *et al.* Characteristics associated with hospitalisation for COVID-19 in people with rheumatic disease: data from the COVID-19 Global Rheumatology Alliance physician-reported registry. *Ann Rheum Dis* 2020;**79**:859–66. doi:10.1136/ANNRHEUMDIS-2020-217871

6 Brenner EJ, Ungaro RC, Gearry RB, *et al.* Corticosteroids, But Not TNF Antagonists, Are Associated With Adverse COVID-19 Outcomes in Patients With Inflammatory Bowel Diseases: Results From an International Registry. *Gastroenterology* 2020;**159**:481-491.e3. doi:10.1053/J.GASTRO.2020.05.032

7 Salter A, Fox RJ, Newsome SD, *et al.* Outcomes and Risk Factors Associated With SARS-CoV-2 Infection in a North American Registry of Patients With Multiple Sclerosis. *JAMA Neurol* 2021;**78**:699–708. doi:10.1001/JAMANEUROL.2021.0688

8 Strangfeld A, Schäfer M, Gianfrancesco MA, *et al.* Factors associated with COVID-19-related death in people with rheumatic diseases: results from the COVID-19 Global

For peer review only - http://bmjopen.bmj.com/site/about/guidelines.xhtml

Rheumatology Alliance physician-reported registry. *Ann Rheum Dis* 2021;**80**:930–42. doi:10.1136/ANNRHEUMDIS-2020-219498

9 Lee ARY Bin, Wong SY, Chai LYA, *et al.* Efficacy of covid-19 vaccines in immunocompromised patients: systematic review and meta-analysis. *BMJ* 2022;**376**:e068632. doi:10.1136/BMJ-2021-068632

10 Boekel L, Steenhuis M, Hooijberg F, *et al.* Antibody development after COVID-19 vaccination in patients with autoimmune diseases in the Netherlands: a substudy of data from two prospective cohort studies. *Lancet Rheumatol* 2021;**3**:e778–88. doi:10.1016/S2665-9913(21)00222-8

11 Alexander JL, Kennedy NA, Ibraheim H, *et al.* COVID-19 vaccine-induced antibody responses in immunosuppressed patients with inflammatory bowel disease (VIP): a multicentre, prospective, case-control study. *lancet Gastroenterol Hepatol* 2022;**7**:342–52. doi:10.1016/S2468-1253(22)00005-X

12 de Boer SE, Berger SP, van Leer–Buter CC, *et al.* Enhanced Humoral Immune Response After COVID-19 Vaccination in Elderly Kidney Transplant Recipients on Everolimus Versus Mycophenolate Mofetil-containing Immunosuppressive Regimens. *Transplantation* 2022;**106**. doi:10.1097/TP.0000000000004177

13 Pottegård A, Kristensen KB, Reilev M, *et al.* Existing Data Sources in Clinical Epidemiology: The Danish COVID-19 Cohort. *Clin Epidemiol* 2020;**12**:875–81. doi:10.2147/CLEP.S257519

14 Voldstedlund M, Haarh M, Mølbak K. The Danish Microbiology Database (MiBa) 2010 to 2013. *Euro Surveill* 2014;**19**. doi:10.2807/1560-7917.ES2014.19.1.20667

15 Jensen JS, Jensen DH, Grønhøj C, *et al.* Incidence and survival of oropharyngeal cancer in Denmark: a nation-wide, population-based study from 1980 to 2014. *Acta Oncol (Madr)*

For peer review only

2018;**57**:269–75. doi:10.1080/0284186X.2017.1390251

16 Pottegård A, Schmidt SAJ, Wallach-Kildemoes H, *et al.* Data Resource Profile: The Danish National Prescription Registry. *Int J Epidemiol* 2017;**46**:798. doi:10.1093/IJE/DYW213

17 Maaser C, Sturm A, Vavricka SR, *et al.* ECCO-ESGAR Guideline for Diagnostic Assessment in IBD Part 1: Initial diagnosis, monitoring of known IBD, detection of complications. *J Crohn's Colitis* 2019;**13**:144-164K. doi:10.1093/ECCO-JCC/JJY113

18 Kay J, Upchurch KS. ACR/EULAR 2010 rheumatoid arthritis classification criteria. *Rheumatology* 2012;**51**:vi5–9. doi:10.1093/RHEUMATOLOGY/KES279

19 Espenhain L, Funk T, Overvad M, *et al.* Epidemiological characterisation of the first 785 SARS-CoV-2 Omicron variant cases in Denmark, December 2021. *Euro Surveill* 2021;**26**. doi:10.2807/1560-7917.ES.2021.26.50.2101146

20 Rosenbaum PR, Rubin DB. The central role of the propensity score in observational studies for causal effects. *Biometrika* 1983;**70**:41–55. doi:10.1093/BIOMET/70.1.41

21 Desai RJ, Franklin JM. Alternative approaches for confounding adjustment in observational studies using weighting based on the propensity score: a primer for practitioners. *BMJ* 2019;**367**. doi:10.1136/BMJ.L5657

22 Sakuraba A, Luna A, Micic D. Serologic Response to Coronavirus Disease 2019 (COVID-19) Vaccination in Patients With Immune-Mediated Inflammatory Diseases: A Systematic Review and Meta-analysis. *Gastroenterology* 2022;**162**:88-108.e9. doi:10.1053/J.GASTRO.2021.09.055

23 Singh JA, Guyatt G, Ogdie A, *et al.* 2018 American College of Rheumatology/National Psoriasis Foundation Guideline for the Treatment of Psoriatic Arthritis: *https://doi.org/101177/2475530318812244* 2018;**4**:31–58. doi:10.1177/2475530318812244

24 Larsen L, Jensen MD, Larsen MD, *et al.* The Danish National Registry for Biological

For peer review only

Therapy in Inflammatory Bowel Disease. *Clin Epidemiol* 2016;**8**:607–12. doi:10.2147/CLEP.S99478

25 Cottone M, Sapienza C, Macaluso FS, *et al.* Psoriasis and Inflammatory Bowel Disease. *Dig Dis* 2019;**37**. doi:10.1159/000500116

26 Hernán MA. The hazards of hazard ratios. *Epidemiology* 2010;**21**:13–5. doi:10.1097/EDE.0B013E3181C1EA43

27 Izadi Z, Brenner EJ, Mahil SK, *et al.* Association Between Tumor Necrosis Factor Inhibitors and the Risk of Hospitalization or Death Among Patients With Immune-Mediated Inflammatory Disease and COVID-19. *JAMA Netw open* 2021;**4**. doi:10.1001/JAMANETWORKOPEN.2021.29639

28 Simon D, Tascilar K, Kleyer A, *et al.* Impact of Cytokine Inhibitor Therapy on the Prevalence, Seroconversion Rate, and Longevity of the Humoral Immune Response Against SARS-CoV-2 in an Unvaccinated Cohort. *Arthritis Rheumatol (Hoboken, NJ)* 2022;**74**:783–90. doi:10.1002/ART.42035

29 Simon D, Tascilar K, Fagni F, *et al.* SARS-CoV-2 vaccination responses in untreated, conventionally treated and anticytokine-treated patients with immune-mediated inflammatory diseases. *Ann Rheum Dis* 2021;**80**:1312–6. doi:10.1136/ANNRHEUMDIS-2021-220461

30 Favalli EG, Bugatti S, Klersy C, *et al.* Impact of corticosteroids and immunosuppressive therapies on symptomatic SARS-CoV-2 infection in a large cohort of patients with chronic inflammatory arthritis. *Arthritis Res Ther* 2020;**22**. doi:10.1186/S13075-020-02395-6

31 McKay LI, Cidlowski JA. Physiologic and Pharmacologic Effects of Corticosteroids. Published Online First: 2003.https://www.ncbi.nlm.nih.gov/books/NBK13780/ (accessed 1 Sep 2022).

32 Rahier JF, Moutschen M, van Gompel A, *et al.* Vaccinations in patients with immune-

For peer review only - http://bmjopen.bmj.com/site/about/guidelines.xhtml

mediated inflammatory diseases. *Rheumatology (Oxford)* 2010;**49**:1815–27. doi:10.1093/RHEUMATOLOGY/KEQ183

33 Croce E, Hatz C, Jonker EF, *et al.* Safety of live vaccinations on immunosuppressive therapy in patients with immune-mediated inflammatory diseases, solid organ transplantation or after bone-marrow transplantation - A systematic review of randomized trials, observational studies and case reports. *Vaccine* 2017;**35**:1216–26. doi:10.1016/J.VACCINE.2017.01.048

For peer review only

TABLES & FIGURES

Table 1. Characteristics of immune-mediated inflammatory disease patients at baseline and after propensity score weighting, by exposure to immunosuppressive therapy.

	**Baseline IMID Cohort***			**Weighted IMID Cohort**		
	**Unexposed**	**Exposed**	**SD**	**Unexposed**	**Exposed**	**SD**
**Total, n (%)**	112 675 (100)	39 765 (100)	NA	39 524 (100)	39 765 (100)	NA
**Inflammatory bowel disease, n (%)**	47 001 (41.7)	10 480 (26.4)	NA	10 284 (26.0)	10 480 (26.4)	NA
**Arthropathy, n (%)**	44 669 (39.6)	24 261 (61.0)	NA	24 227 (61.3)	24 261 (61.0)	NA
**Psoriasis, n (%)**	21 005 (18.6)	5 024 (12.6)	NA	5 014 (12.7)	5 024 (12.6)	NA
Age, median (IQR)	59 (46-71)	58 (45-71)	0.06	58 (44-71)	58 (45-71)	0.01
Male, n (%)	48 941 (43.4)	17 080 (43.0)	0.01	16 934 (42.8)	17 080 (43.0)	0.00
SARS-CoV-2 test in the previous month, median (IQR)	0 (0-1)	0 (0-1)	0.04	0 (0-1)	0 (0-1)	0.00
**Calendar date of entry 2021, n (%)**						
January to April	27,865 (24.7)	16,689 (42.0)	0.37	10,124 (25.6)	16,689 (42.0)	0.35
May to August	81,682 (72.5)	22,361 (56.2)	0.34	28,211 (71.4)	22,361 (56.2)	0.32
September to November	3,128 (2.8)	715 (1.8)	0.07	1,189 (3.0)	715 (1.8)	0.08
**Comorbidities, n (%)**						
Cardiovascular disease	41 056 (36.4)	14 010 (35.2)	0.03	14 050 (35.5)	14 010 (35.2)	0.01
Pulmonary disease	14 994 (13.3)	5 793 (14.6)	0.04	5796 (14.7)	5 793 (14.6)	0.00
Liver disease	3 630 (3.2)	1 528 (3.8)	0.03	1 525 (3.9)	1 528 (3.8)	0.00
Kidney disease	6 859 (6.1)	2 260 (5.7)	0.02	2 279 (5.8)	2 260 (5.7)	0.00
Other gastrointestinal diseases	21 505 (19.1)	7 086 (17.8)	0.03	6 984 (17.7)	7 086 (17.8)	0.00
Skin disease	9 889 (8.8)	3 748 (9.4)	0.02	3 703 (9.4)	3 748 (9.4)	0.00
Musculoskeletal disease	45 393 (40.3)	16 620 (41.8)	0.03	16 649 (42.1)	16 620 (41.8)	0.01
**Medications, n (%)**						
Cardiovascular drugs	80 529 (71.5)	28 179 (70.9)	0.01	28 054 (71.0)	28 179 (70.9)	0.00
Antibiotics	108 502 (96.3)	38 405 (96.6)	0.02	38 181 (96.6)	38 405 (96.6)	0.00
Oral anticoagulants	11 111 (9.9)	4 022 (10.1)	0.01	4 034 (10.2)	4 022 (10.1)	0.00
Drugs used in diabetes	12 935 (11.5)	4 034 (10.1)	0.04	4 030 (10.2)	4 034 (10.1)	0.00

For peer review only

For peer review only - http://bmjopen.bmj.com/site/about/guidelines.xhtml

Drugs for obstructive airway diseases	36 970 (32.8)	13 118 (33.0)	0.00	13 116 (33.2)	13 118 (33.0)	0.00
**IBD-specific treatments, n (%)**						
Any IBD-related hospital admissions in the previous year	564 (1.2)	714 (6.8)	0.12	584 (5.7)	714 (6.8)	0.05
5-ASA/sulfasalazine	12 596 (26.8)	2 845 (27.1)	0.00	2 857 (27.8)	2 845 (27.1)	0.01
Budesonide	646 (1.4)	272 (2.6)	0.02	277 (2.7)	272 (2.6)	0.01
IBD related procedures	18 553 (39.5)	4 551 (43.4)	0.05	4 473 (43.5)	4 551 (43.4)	0.00
Endoscopy of the gastrointestinal tract	7 454 (15.9)	3 702 (35.3)	0.12	3 543 (34.5)	3 702 (35.3)	0.02
**Arthropathy-specific treatments, n (%)**						
Arthropathy-related procedures	13 932 (31.2)	8 415 (34.7)	0.09	8 399 (34.7)	8 415 (34.7)	0.00
Anti-inflammatory and anti-rheumatic drugs	5 678 (12.7)	3 462 (14.3)	0.07	3 480 (14.4)	3 462 (14.3)	0.00
Hydroxychloroquine	540 (1.2)	845 (3.5)	0.15	801 (3.3)	845 (3.5)	0.01
**Psoriasis-specific treatments, n (%)**						
5-ASA/sulfasalazine	2 149 (10.2)	807 (16.1)	0.03	805 (16.1)	807 (16.1)	0.00
Topical corticosteroids	73 (0.3)	42 (0.8)	0.00	39 (0.8)	42 (0.8)	0.01
Anti-psoriatic medication	7 441 (35.4)	1 894 (37.7)	0.01	1 900 (37.9)	1 894 (37.7)	0.00
Topical calcineurin inhibitors	3 731 (17.8)	1 106 (22.0)	0.00	1 110 (22.1)	1 106 (22.0)	0.00
Psoriasis related procedures	542 (2.6)	144 (2.9)	0.00	144 (2.9)	144 (2.9)	0.00
5-ASA/sulfasalazine	2 149 (10.2)	807 (16.1)	0.03	805 (16.1)	807 (16.1)	0.00

Abbreviations: IMID = immune-mediated inflammatory disease; SD = standardized differences; IQR = inter-quartile range; NA = not applicable. *Total cohort numbers prior to trimming.

For peer review only

FIGURE LEGENDS

Figure 1. Flow chart for inclusion into the exposed and unexposed IMID cohort.

Figure 2. Risk of infection, hospitalization and death associated with immunosuppressive exposure from Jan 2021-Nov 2021 in Inflammatory Bowel Disease, Arthropathy, Psoriasis and across all IMID cohorts.

Figure 3. Risk of infection associated with immunosuppressive drug class exposure (Selective Immunosuppressants, Tumour Necrosis Factor Inhibitors, Interleukin Inhibitors, Calcineurin Inhibitors, Systemic Corticosteroids, and Other Immunosuppressants including Rituximab) in IMID patients.

Figure 4. Risk of hospitalization associated with immunosuppressive drug class exposure (Selective Immunosuppressants, Tumour Necrosis Factor Inhibitors, Interleukin Inhibitors, Calcineurin Inhibitors, Systemic Corticosteroids, and Other Immunosuppressants including Rituximab) in IMID patients.

For peer review only - http://bmjopen.bmj.com/site/about/guidelines.xhtml

Figure 1.

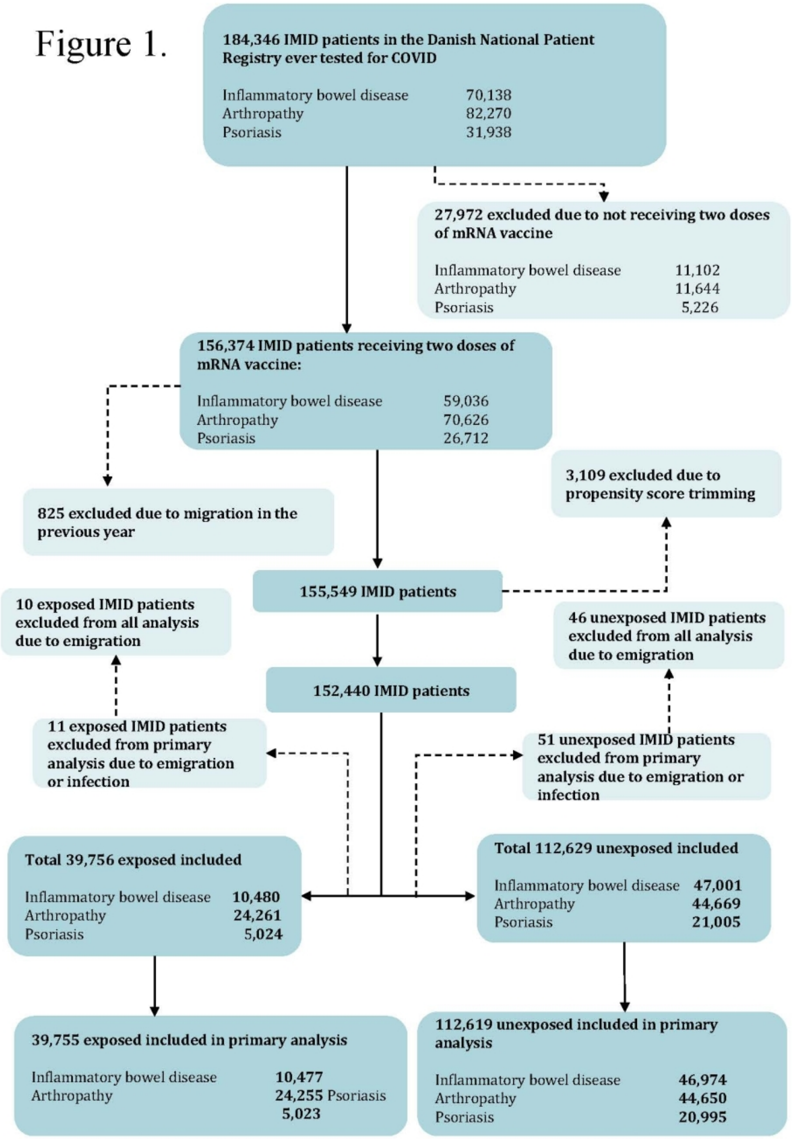

Figure 1. Flow chart for inclusion into the exposed and unexposed IMID cohort.

190x275mm (200 x 200 DPI)

Figure 2.

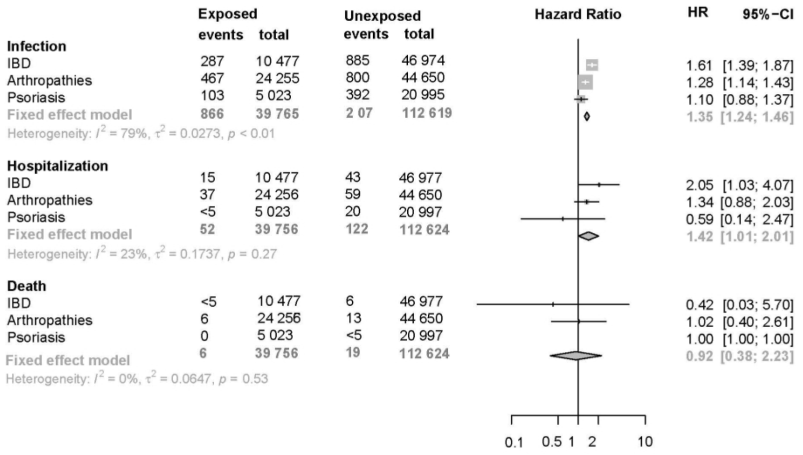

Figure 2. Risk of infection, hospitalization and death associated with immunosuppressive exposure from Jan 2021-Nov 2021 in Inflammatory Bowel Disease, Arthropathy, Psoriasis and across all IMID cohorts.

296x209mm (200 x 200 DPI)

Figure 3.

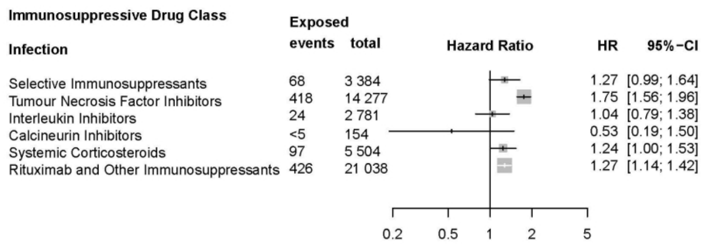

Figure 3. Risk of infection associated with immunosuppressive drug class exposure (Selective Immunosuppressants, Tumour Necrosis Factor Inhibitors, Interleukin Inhibitors, Calcineurin Inhibitors, Systemic Corticosteroids, and Other Immunosuppressants including Rituximab) in IMID patients.

296x209mm (200 x 200 DPI)

Figure 4.

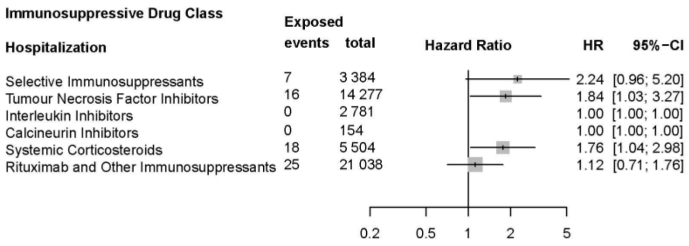

Figure 4. Risk of hospitalization associated with immunosuppressive drug class exposure (Selective Immunosuppressants, Tumour Necrosis Factor Inhibitors, Interleukin Inhibitors, Calcineurin Inhibitors, Systemic Corticosteroids, and Other Immunosuppressants including Rituximab) in IMID patients.

296x209mm (200 x 200 DPI)

The Impact of Immunosuppressive Therapy on SARS-CoV-2 mRNA Vaccine Effectiveness in Patients with Immune-mediated Inflammatory Diseases: A Danish Nationwide Cohort Study

Rahma Elmahdi[*1,2] (0000-0002-7013-9548), Daniel Ward[*1] (0000-0003-0405-7250), Martin Thomsen Ernst[3] (0000-0001-9003-3823), Gry Juul Poulsen[1] (0000-0003-2744-2469), Jesper Hallas[3] (0000-0002-8097-8708), Anton Pottegård[3] (0000-0001-9314-5679), Tine Jess[1,4] (0000-0002-4391-7332)

For peer review only

SUPPLEMNETRAY TABLES

Supplementary Table 1. Exposures and corresponding ATC and procedure codes for immunosuppressives

Medication	ATC code	Procedure code
**Selective immunosuppressants**		
Muromonab-CD3	L04AA02	
Antilymphocyte immunoglobulin	L04AA03	
(horse)	L04AA04	BOHJ12
Antithymocyte immunoglobulin (rabbit)	L04AA06	BOHJ22
Mycophenolic acid	L04AA10	BOHJ23
Sirolimus	L04AA13	
Leflunomide	L04AA18	BOHJ24
Everolimus	L04AA23	BOHJ26
Natalizumab	L04AA24	BOHJ18C1
Abatacept	L04AA25	BWHB84
Eculizumab	L04AA26	BOHJ19H6
Belimumab	L04AA27	BOHJ27
Fingolimod	L04AA28	
Belatacept	L04AA29	BOHJ28D
Tofacitinib	L04AA31	BOHJ28A
Teriflunomide	L04AA32	
Aprelimast	L04AA33	BOHJ19H4
Vedolizumab	L04AA34	BOHJ16A
Alemtuzumab	L04AA36	
Ocrelizumab	L04AA37	
Baricitinib	L04AA38	
Ozanimod	L04AA39	
Emapalumab	L04AA40	BWHA178
Cladribine	L04AA41	
Imlifidase	L04AA42	BWHB87
Siponimod	L04AA43	
Ravulizumab	L04AA44	
Upadacitinib		

**Tumour necrosis factor inhibitors**		
Etanercept	L04AB01	BOHJ18A2
Infliximab	L04AB02	BOHJ18A1
Adalimumab	L04AB04	BOHJ18A3
Certolizumab pegol	L04AB05	BOHJ18A5
Golimumab	L04AB06	BOHJ18A4
**Interleukin inhibitors**		
Basiliximab	L04AC02	
Anakinra	L04AC03	BOHJ18B1
Ustekinumab	L04AC05	BOHJ18B3
Tocilizumab	L04AC07	BOHJ18B2
Canakinumab	L04AC08	BOHJ18B4
Secukinumab	L04AC10	BOHJ18B5
Siltuximab	L04AC11	
Brodalumab	L04AC12	BOHJ18B6
Ixekizumab	L04AC13	
Sarilumab	L04AC14	BOHJ18B9
Guselkumab	L04AC16	
Tildrakizumab	L04AC17	BOHJ18B7
Risankizumab	L04AC18	BOHJ19N1
**Calcineurin inhibitors**		
Ciclosporin	L04AD01	BOHJ20
Tacrolimus	L04AD02	BOHJ21
**Other immunosuppressants**		
Azathioprine	L04AX01	BWHB83
Thalidomide	L04AX02	BWHB81
Methotrexate	L04AX03	BWHA115
Lenalidomide	L04AX04	BWHB82
Pirfenidone	L04AX05	BWHB85
Pomalidomide	L04AX06	BWHB86
Dimethyl fumarate	L04AX07	BOHJ28B
Darvadstrocel	L04AX08	
**Systemic corticosteroids**	H02AB	
**Rituximab**	L01XC02	

For peer review only

Supplementary Table 2. Minimum daily dose of corticosteroid in exposed persons

Compound	Equivalent dose
Cortisone	38mg
Hydrocortisone	30mg
Prednisone	7.5mg
Prednisolone	7.5mg
Triamcinolone	6mg
Methylprednisolone	6mg
Betamethasone	0.9mg
Dexamethasone	1.2mg

For peer review only

Supplementary Table 3. Covariates by disease cohort

IBD COHORT		
**Covariate**	**Categories/ ATC/ICD codes**	**Assessment window**
Age	-	At cohort entry
Sex	-	At cohort entry
SARS-CoV-2 tests in the previous month	-	At cohort entry
Ever previous SARS-CoV-2 positive test	-	From February 2020
IBD specific covariates		
Any IBD–related hospital admissions in the previous year	K50/K51	1 year before cohort entry
Crohn's disease	K50	At cohort entry
Ulcerative colitis	K51	At cohort entry
5-ASA/sulfasalazine	MA07EC0	1 year before matching date
Budesonide	MA07EA06	3 months before matching date
IBD related procedures		Any time before cohort entry
Operations of the small bowel and colon	KJF	
Operations of the rectum	KJG	
Operations of the anus and perianal tissue	KJH	
Operational of the abdominal wall, peritoneum, mesentery and omentum	KJA	
Lysis of adhesion in the abdominal cavity	KJAP	
Closure of intestinovaginal fistula	KLEE30	
Closure of vesiculointestinal fisutal	KKCH30	
Endoscopy of the gastrointestinal tract	KUJ	1 year before the cohort entry
Comorbidities		
Cardiovascular disease	DI1-I7	Any time before cohort entry
Pulmonary disease		Any time before cohort entry
Chronic disease of the lower airways	DJ4	
Other interstitial lung disease	DJ84	
Diseases with pus and necrosis in the lower airway	DJ85	
Interstitial lung emphysema	DJ982	
compensatory emphysema	DJ983	
Liver disease	DK70-K77	Any time before cohort entry
Kidney disease		Any time before cohort entry
Glomerular disease	DN0	
Tubulointerstitial kidney disease and kidney insufficiency	DN1	
Other gastrointestinal diseases		Any time before cohort entry
Gingivitis and periodontal disease	DK05	
Inflammation of the oral mucosa and related	DK12	
Stomach and duodenal ulcers	DK25-27	
Fissure and rifts in and around the anus	DK60	
Abscess in and around the anus	DK61	
Other diseases of the rectum and anus	DK62	
Other bowel disease	DK63	

For peer review only

Skin disease		Any time before cohort entry
Bullous skin disease	DL10-14	
Dermatitis and eczema	DL20-30	
Alopecia areata	DL63	
Vitiligo	DL80	
Granulomatous disease in the skin and subcutaneous tissue	DL92	
Lupus	DL93	
Other localised connective tissue disease	DL94	
Vasculitis limited to the skin	DL95	
Musculoskeletal disease		Any time before cohort entry
Generalised connective tissue diseases	DM30-	
Diseases of the muscles	DM36	
Soft-tissue rheumatism	DM60-63	
	DM70-79	
Medications		
Cardiovascular drugs	MC01-MC10	Any time before cohort entry
Antibiotics	MJ01	Any time before cohort entry
Oral anticoagulants	MB01AA MB01AF	Any time before cohort entry
Drugs used in diabetes	MA10	Any time before cohort entry
Drugs for obstructive airway diseases	MR03	Any time before cohort entry
INFLAMMATORY ARTHROPATHIES COHORT		
Age	-	At cohort entry
Sex	-	At cohort entry
SARS-CoV-2 tests in the previous month	-	At cohort entry
Arthropathy-specific covariates		
Arthropathy-related procedures		Any time before cohort entry
Shoulder and upper arm		
Primary insertion of joint prosthesis	KNBB	
Secondary insertion of joint prosthesis	KNBC	
Operations on the joint capsule and ligaments	KNBE	
Operations of the synovia and joint surface	KNBF	
Joint resections, arthroplasties, and arthrodesis	KNBG	
Excision of bursa	KBM79	
Elbow and lower arm		
Primary insertion of joint prosthesis	KNCB	
Secondary insertion of joint prosthesis	KNCC	
Operations on the joint capsule and ligaments	KNCE	
Operations of the synovia and joint surface	KNCF	
Joint resections, arthroplasties, and arthrodesis	KNCG	
Excision of bursa	KNCM79	
Wrist and hand		
Primary insertion of joint prosthesis	KNDB	
Secondary insertion of joint prosthesis	KNDC	
Operations on the joint capsule and ligaments	KNDE	
Operations of the synovia and joint surface	KNDF	
Joint resections, arthroplasties, and arthrodesis	KNDG	
Operations on the fascia, tendon sheaths, ganglia and bursae	KNDM	

For peer review only

Hip and thigh<br>Primary insertion of joint prosthesis<br>Secondary insertion of joint prosthesis<br>Operations on the joint capsule and ligaments<br>Operations of the synovia and joint surface<br>Joint resections, arthroplasties, and arthrodesis<br>Excision of bursa in the<br>Knee and lower leg<br>Primary insertion of joint prosthesis<br>Secondary insertion of joint prosthesis<br>Operations on the joint capsule and ligaments<br>Operations of the synovia and joint surface<br>Joint resections, arthroplasties, and arthrodesis<br>Excision of bursae<br>Ankle and foot<br>Primary insertion of joint prosthesis<br>Secondary insertion of joint prosthesis<br>Operations on the joint capsule and ligaments<br>Operations of the synovia and joint surface<br>Joint resections, arthroplasties, and arthrodesis<br>Operations on the fascia, tendon sheaths, ganglia and bursae	 KNFB<br>KNFC<br>KNFE<br>KNFF<br>KNFG<br>KNFM79<br><br>KNGB<br>KNGC<br>KNGE<br>KNGF<br>KNGG<br>KNGM79<br><br>KNHB<br>KNHC<br>KNHE<br>KNHF<br>KNHG<br>KNHM	
Anti-inflammatory and anti-rheumatic drugs<br>inc. specific anti-rheumatic therapies, non-steroidals, and combination medications<br>Hydroxychloroquine	 M01<br><br>P01BA02	3 months before before cohort entry
Comorbidities		
Cardiovascular disease	DI1-I7	Any time before cohort entry
Pulmonary disease<br>Chronic disease of the lower airways<br>Other interstitial lung disease<br>Diseases with pus and necrosis in the lower airway<br>Interstitial lung emphysema<br>compensatory emphysema	 DJ4<br>DJ84<br>DJ85<br>DJ982<br>DJ983	Any time before cohort entry
Liver disease	DK70-K77	Any time before cohort entry
Kidney disease<br>Glomerular disease<br>Tubulointerstitial kidney disease and kidney insufficiency	 DN0<br>DN1	Any time before cohort entry
Other gastrointestinal diseases<br>Gingivitis and periodontal disease<br>Inflammation of the oral mucosa and related<br>Stomach and duodenal ulcers<br>Fissure and rifts in and around the anus<br>Abscess in and around the anus<br>Other diseases of the rectum and anus<br>Other bowel disease	 DK05<br>DK12<br>DK25-27<br>DK60<br>DK61<br>DK62<br>DK63	Any time before cohort entry
Skin disease<br>Bullous skin disease<br>Dermatitis and eczema<br>Alopecia areata	 DL10-14<br>DL20-30<br>DL63	Any time before cohort entry

For peer review only

Vitiligo<br>Granulomatous disease in the skin and subcutaneous tissue<br>Lupus<br>Other localised connective tissue disease<br>Vasculitis limited to the skin	DL80<br>DL92<br>DL93<br>DL94<br>DL95	
Musculoskeletal disease<br>Generalised connective tissue diseases<br>Diseases of the muscles<br>Soft-tissue rheumatism	DM30-<br>DM36<br>DM60-63<br>DM70-79	Any time before cohort entry
Medications		
Cardiovascular drugs	MC01-MC10	Any time before cohort entry
Antibiotics	MJ01	Any time before cohort entry
Oral anticoagulants	MB01AA<br>MB01AF	Any time before cohort entry
Drugs used in diabetes	MA10	Any time before cohort entry
Drugs for obstructive airway diseases	MR03	Any time before cohort entry
PSORIASIS COHORT		
Age	-	At cohort entry
Sex	-	At cohort entry
SARS-CoV-2 tests in the previous month	-	At cohort entry
Psoriasis-specific covariates		
Procedures<br>Ultraviolet therapy: UV-A with psoralen/broad-spectrum UVB / narrow-spectrum UVB	BNGA1/<br>BNGA2/<br>BNGA3	Any time before cohort entry
Therapeutic steroid injection in the joints and soft tissues	BLHN0	Any time before cohort entry
5-ASA/sulfasalazine	A07EC0	1 year before cohort entry
Topical corticosteroids	D07	1 year before cohort entry
Antipsoriatic medication	D05	1 year before cohort entry
Topical calcineurin inhibitors	D11AH01-02,	1 year before cohort entry
Comorbidities		
Cardiovascular disease	DI1-I7	Any time before cohort entry
Pulmonary disease<br>Chronic disease of the lower airways<br>Other interstitial lung disease<br>Diseases with pus and necrosis in the lower airway<br>Interstitial lung emphysema<br>compensatory emphysema	DJ4<br>DJ84<br>DJ85<br>DJ982<br>DJ983	Any time before cohort entry

For peer review only

Liver disease	DK70-K77	Any time before cohort entry
Kidney disease		Any time before cohort entry
Glomerular disease	DN0	
Tubulointerstitial kidney disease and kidney insufficiency	DN1	
Other gastrointestinal diseases		Any time before cohort entry
Gingivitis and periodontal disease	DK05	
Inflammation of the oral mucosa and related	DK12	
Stomach and duodenal ulcers	DK25-27	
Fissure and rifts in and around the anus	DK60	
Abscess in and around the anus	DK61	
Other diseases of the rectum and anus	DK62	
Other bowel disease	DK63	
Skin disease		Any time before cohort entry
Bullous skin disease	DL10-14	
Dermatitis and eczema	DL20-30	
Alopecia areata	DL63	
Vitiligo	DL80	
Granulomatous disease in the skin and subcutaneous tissue	DL92	
Lupus	DL93	
Other localised connective tissue disease	DL94	
Vasculitis limited to the skin	DL95	
Musculoskeletal disease		Any time before cohort entry
Generalised connective tissue diseases	DM30-	
Diseases of the muscles	DM36	
Soft-tissue rheumatism	DM60-63	
	DM70-79	
Medications		
Cardiovascular drugs	MC01-MC10	Any time before cohort entry
Antibiotics	MJ01	Any time before cohort entry
Oral anticoagulants	MB01AA MB01AF	Any time before cohort entry
Drugs used in diabetes	MA10	Any time before cohort entry
Drugs for obstructive airway diseases	MR03	Any time before cohort entry

For peer review only

Supplementary Table 4. Risk of infection, hospitalisation and death associated with immunosuppressive therapy in vaccinated patients with immune-mediated inflammatory diseases.

	**Exposed**				**Unexposed**				**Crude HR (95% CI)** ***	**Weighted HR (95% CI)** ***
	**Total**	**Events**	**Incidence rate (95% CI)** **	**PY**	**Total**	**Events**	**Incidence rate (95% CI)** **	**PY**		
**COVID-19 Infection***										
IBD	10 477	287	54.7 (48.7-61.4)	5 246	46 974	885	42.5 (39.8-45.4)	20 809	1.7 (1.5-1.9)	1.6 (1.4-1.9)
Arthropathy	24 255	476	40.3 (36.8-44.1)	11 822	44 650	800	37.8 (35.3-40.6)	21 140	1.3 (1.1-1.4)	1.3 (1.1-1.4)
Psoriasis	5 023	103	45.3 (37.4-55.00)	2 273	20 995	392	41.5 (37.6-45.8)	9 441	1.1 (0.9-1.4)	1.1 (0.9-1.4)
Combined cohorts	39 755	866	-	19 341	112 619	2 077	-	51 390	1.4 (1.3-1.5)	1.4 (1.2-1.5)
**COVID-19 Hospitalization***										
IBD	10 477	15	2.8 (1.7-4.7)	5 280	46 980	43	2.1 (1.5-2.8)	20 892	2.8 (1.5-5.1)	2.1 (1.0-4.1)
Arthropathy	24 256	37	3.1 (2.3-4.3)	11 873	44 650	59	2.8 (2.2-3.6)	21 215	1.3 (0.9-2.0)	1.3 (0.9-2.0)
Psoriasis	5 023	n<5	-	2 284	20 999	20	2.1 (1.4-3.3)	9 479	0.5 (0.1-2.3)	0.6 (0.1-2.5)
Combined cohorts	39 756	52	-	19 437	112 629	122	-	51 594	1.6	1.4

For peer review only

									(1.1-2.2)	(1.0-2.0)
**Death***										
IBD	10 477	n<5	-	5 281	46 980	6	0.3 (0.1-0.6)	20 897	1.1 (0.1-9.7)	0.4 (0-5.6)
Arthropathy	24 256	6	0.51 (0.2-1.1)	11 875	44 650	13	0.6 (0.4-1.1)	21 222	1.0 (0.4-2.7)	1.0 (0.4-2.6)
Psoriasis	5 023	0	-	2 284	20 999	n<5	-	9 480	-	-
Combined cohorts	39 756	6	-	19 440	112 629	19	-	51 599	1.0 (0.4-2.5)	0.9 (0.4-2.2)

Abbreviations: CI = confidence interval; HR = hazard ratio; IBD= inflammatory bowel disease; PY = person-years.

[*]COVID19- Infection = confirmed positive antigen or PCR test result for SARS-CoV2; COVID-19 Hospital Admission = Hospitalisation with SARS-CoV2 infection as primary diagnosis code; Death within 60 days of positive SARS-CoV2 test.

[**]Events/1000 PY (95% CI)

[***] Sex and age adjusted crude and weighted hazard ratios

For peer review only

Supplementary Table 5. Risk of COVID-19 infection associated with immunosuppressive therapy in vaccinated patients with immune-mediated inflammatory diseases at 0-3 months, 3-6 months, and 6-11 months post-vaccination.

	**Exposed**				**Unexposed**				**Crude HR (95% CI) ****	**Weighted HR (95% CI) ****
	**Total**	**Events**	**Incidence rate (95% CI) ***	**PY**	**Total**	**Events**	**Incidence rate (95% CI) ***	**PY**		
**COVID-19 infection 0-3 months post-vaccination**										
IBD	10 477	57	22.2 (17.1-28.8)	2 570	46 974	196	17.0 (14.8-19.5)	11 546	1.6 (1.2-2.2)	1.5 (1.1-2.2)
Arthropathy	24 255	96	16.1 (13.2-19.6)	5 978	44 650	166	15.1 (13.0-17.6)	10 990	1.3 (1.0-1.7)	1.3 (1.0-1.7)
Psoriasis	5 023	24	19.4 (13.0-29.0)	1 236	20 995	83	16.1 (13.0-19.9)	5 163	1.3 (0.3-2.1)	1.3 (0.8-2.1)
Combined cohorts	39 755	177	-	4 403	112 619	445	-	27 699	1.4 (1.2-1.7)	1.4 (1.1-1.6)
**COVID-19 infection 3-6 months post-vaccination**										
IBD	10 010	123	45.8 (38.4-54.7)	2 683	45 202	526	56.8 (52.1-61.8)	9 266	0.9 (0.8-1.2)	1.0 (0.8-1.2)
Arthropathy	23 503	284	48.6 (43.2-54.5)	5 849	43 186	482	47.5 (43.4-51.9)	10 149	1.2 (1.1-1.4)	1.2 (1.1-1.4)
Psoriasis	4 860	63	60.7 (47.4-77.7)	1 038	20 203	233	54.5 (47.9-61.9)	4 279	1.1 (0.8-1.4)	1.0 (0.8-1.4)
Combined cohorts	38 373	470	-	19 437	108 591	1 241	-	23 694	1.1 (1.0-1.2)	1.1 (1.0-1.3)

**COVID-19 infection 6-11 months post-vaccination**										
IBD	6 058	107	160.6 (132.9-194.1)	666	15 742	163	107.1 (91.9-124.9)	1 522	1.3 (0.9-1.7)	1.4 (1.1-1.9)
Arthropathy	12 223	96	82.7 (67.7-101.0)	1 160	19 839	152	80.8 (68.9-94.7)	1 881	1.1 (0.8-1.4)	1.1 (0.8-1.4)
Psoriasis	1 719	16	93.8 (57.5-153.1)	170	7 444	76	109.8 (87.7-137.5)	691	0.9 (0.5-1.5)	0.9 (0.5-1.5)
Combined cohorts	20 000	219	-	1 996	43 025	391	-	4 094	1.1 (0.9-1.3)	1.11 (0.9-1.3)

Abbreviations: CI = confidence interval; HR = hazard ratio; IBD= inflammatory bowel disease; PY = person-years.

*Events/1000 PY

**Sex and age adjusted crude and weighted hazard ratios

For peer review only

Supplementary Table 6. Risk of COVID infection associated with immunosuppressive therapy in vaccinated patients with immune-mediated inflammatory diseases by calendar period (1st January-31st November 2021)

	Exposed		Unexposed		Hazard Ratio (HR)	
	**Total**	**Events**	**Total**	**Events**	**Crude HR (95% CI)**	**Weighted HR (95% CI)** **
***COVID-19 infection from 1st January to 30th April**						
IBD	5 745	n<5	10 306	7	0.67 (0.17-2.61)	0.39 (0.09-1.71)
Arthropathy	9 646	9	13 044	17	0.75 (0.33-1.69)	0.78 (0.35-1.71)
Psoriasis	1 298	n<5	4 512	6	2.00 (0.50-8.02)	1.98 (0.48-8.19)
Combined IMID	16 689	<19	27 862	30	0.87 (0.48-1.59)	0.83 (0.44-1.54)
**COVID-19 infection 1st May to 31st August**						
IBD	10 141	56	45 486	121	1.61 (1.17-2.22)	1.19 (0.82-1.73)
Arthropathy	23 885	74	43 457	116	1.08 (0.81-1.45)	1.07 (0.79-1.43)
Psoriasis	4 912	12	20 337	53	0.88 (0.47-1.65)	0.84 (0.44-1.58)
Combined IMID	38 938	142	109 280	290	1.24 (1.01-1.52)	1.08 (0.87-1.34)
**COVID-19 infection 1st September to 30st November**						
IBD	1 0349	228	46 590	757	1.69 (1.45-1.97)	1.74 (1.48-2.04)
Arthropathy	23 898	393	44 079	667	1.34 (1.18-1.51)	1.33 (1.17-1.51)
Psoriasis	4 974	88	20 803	333	1.17 (0.93-1.48)	1.13 (0.89-1.44)
Combined IMID	39 221	709	111 472	1757	1.42 (1.30-1.55)	1.42 (1.29-1.56)

Abbreviations: CI = confidence interval; HR = hazard ratio; IBD= inflammatory bowel disease.

*COVID19- Infection = confirmed positive antigen or PCR test result for SARS-CoV2

** Sex and age adjusted crude and weighted hazard ratios

For peer review only

Supplementary Table 7. Risk of infection, hospitalisation and death associated with immunosuppressive therapy in vaccinated patients with immune-mediated inflammatory diseases by immunosuppressive drug class.

	**Exposed**				**Unexposed**				**Crude HR (95% CI)** ***	**Weighted HR (95% CI)** ***
	**Total**	**Events**	**PY**	**Incidence rate (95% CI)** **	**Total**	**Events**	**PY**	**Incidence rate (95% CI)** **		
**COVID-19 Infection***										
Selective Immunosuppressants	3 384	68	1 846	39.4 (31.0-49.9)	151 936	2 079	51 334	40.5 (38.8-42.3)	1.3 (1.0-1.7)	1.3 (1.0-1.6)
Tumour Necrosis Factor Inhibitors	14 277	418	6 908	62.5 (56.7-68.7)	140 797	2 070	51 355	40.3 (38.6-42.1)	1.8 (1.6-2.0)	1.8 (1.6-2.0)
Interleukin Inhibitors	2 781	52	1 385	40.1 (30.6-52.7)	152 584	2 082	51 352	40.5 (38.8-42.3)	1.1 (0.8-1.4)	1.0 (0.8-1.4)
Calcineurin Inhibitors	154	n<5	129	-	155 309	2 073	51 298	40.4 (38.7-42.2)	1.3 (0.3-5.2)	0.5 (0.2-1.5)
Systemic Corticosteroids	5 504	97	2 991	34.6 (28.4-42.3)	149 667	2 079	51 503	40.4 (38.7-42.1)	1.3 (1.0-1.6)	1.2 (1.0-1.5)
Rituximab and Other Immunosuppressants	21 038	426	10 703	41.2 (37.5-45.3)	112 593	2 075	51 409	40.4 (38.7-42.1)	1.3 (1.2-1.4)	1.3 (1.1-1.4)
**COVID-19 Hospitalization***										
Selective Immunosuppressants	3 384	7	1 735	4.0 (1.9-8.5)	112 479	122	51 531	2.4 (2.0-2.8)	3.0 (1.4-6.5)	2.2 (1.0-5.2)
Tumour Necrosis Factor Inhibitors	14 277	16	6 740	2.4 (1.5-3.9)	112 506	116	51 551	2.3 (1.9-2.1)	2.0 (1.2-3.3)	1.8 (1.0-3.3)

Interleukin Inhibitors	2 781	0	1 302	-	112 445	125	51 549	2.4 (2.0-2.9)	-	-
Calcineurin Inhibitors	154	0	83	-	112 404	122	51 493	2.4 (2.0-2.8)	-	-
Systemic Corticosteroids	5 504	18	2 811	6.4 (4.0-10.2)	112 587	122	51 699	2.4 (2.0-2.8)	2.4 (1.4-3.9)	1.8 (1.0-3.0)
Rituximab and Other Immunosuppressants	21 038	25	10 387	2.4 (1.6-3.6)	112 593	121	51 604	2.3 (2.0-2.8)	1.2 (0.5-1.0)	1.1 (0.7-1.8)
**Death***										
Selective Immunosuppressants	3 384	0	1 736	-	112479	23	51 545	0.5 (0.3-0.7)	-	-
Tumour Necrosis Factor Inhibitors	14 277	0	6 741	-	112506	19	5 1564	0.4 (0.2-0.6)	-	-
Interleukin Inhibitors	2 781	0	1 302	-	112 445	24	51 564	0.5 (0.3-0.7)	-	-
Calcineurin Inhibitors	154	0	83	-	112 404	24	51 507	0.5 (0.3-0.7)	-	-
Systemic Corticosteroids	5 504	6	2 812	2.1 (2.0-4.8)	112 587	23	51 713	0.4 (0.3-0.7)	4.0 (1.6-9.9)	2.3 (0.9-6.2)
Rituximab and Other Immunosuppressants	21 038	n<5	10 388	-	112 593	22	51 619	0.4 (0.3-0.7)	0.5 (0.1-2.3)	0.5 (0.1-2.4)

For peer review only

Abbreviations: CI = confidence interval; HR = hazard ratio; IBD= inflammatory bowel disease; PY = person-years.

Immunosuppressive Drug Class: Selective Immunosuppressants, Tumour Necrosis Factor Inhibitors, Interleukin Inhibitors, Calcineurin Inhibitors, Systemic Corticosteroids, and Other Immunosuppressants including Rituximab (See Supplementary for complete ATC codes)

*COVID19- Infection = confirmed positive antigen or PCR test result for SARS-CoV2; COVID-19 Hospital Admission = Hospitalisation with SARS-CoV2 infection as primary diagnosis code; Death within 60 days of positive SARS-CoV2 test.

**Events/1000 PY

*** Sex and age adjusted crude and weighted hazard ratios

For peer review only

SUPPLEMNETRAY FIGURES

Supplementary Figure 1. Kaplan-Meier plots for probability of infection with COVID-19 following second mRNA vaccination in immunosuppressive exposed compared to propensity score matched immunosuppressive unexposed a. IBD patients b. Arthropathy patients c. Psoriasis patients over the calendar year from January 1st to 31st November 2021. SMR weighted = standardized mortality ratio weighted.

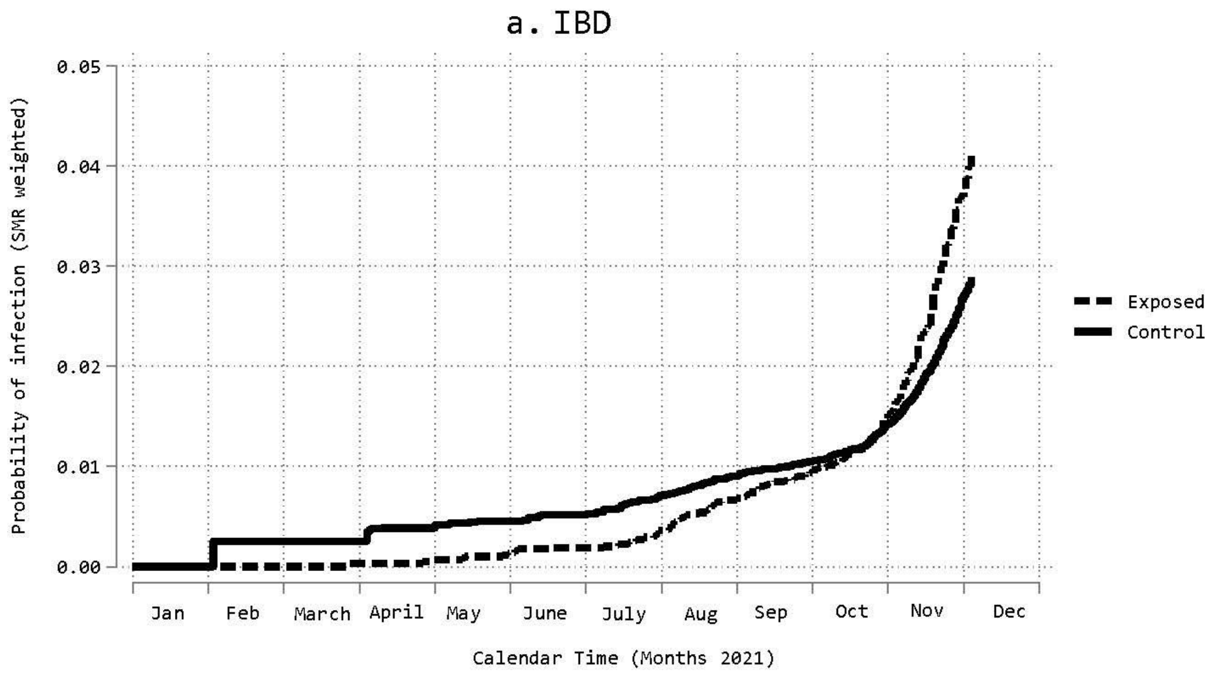

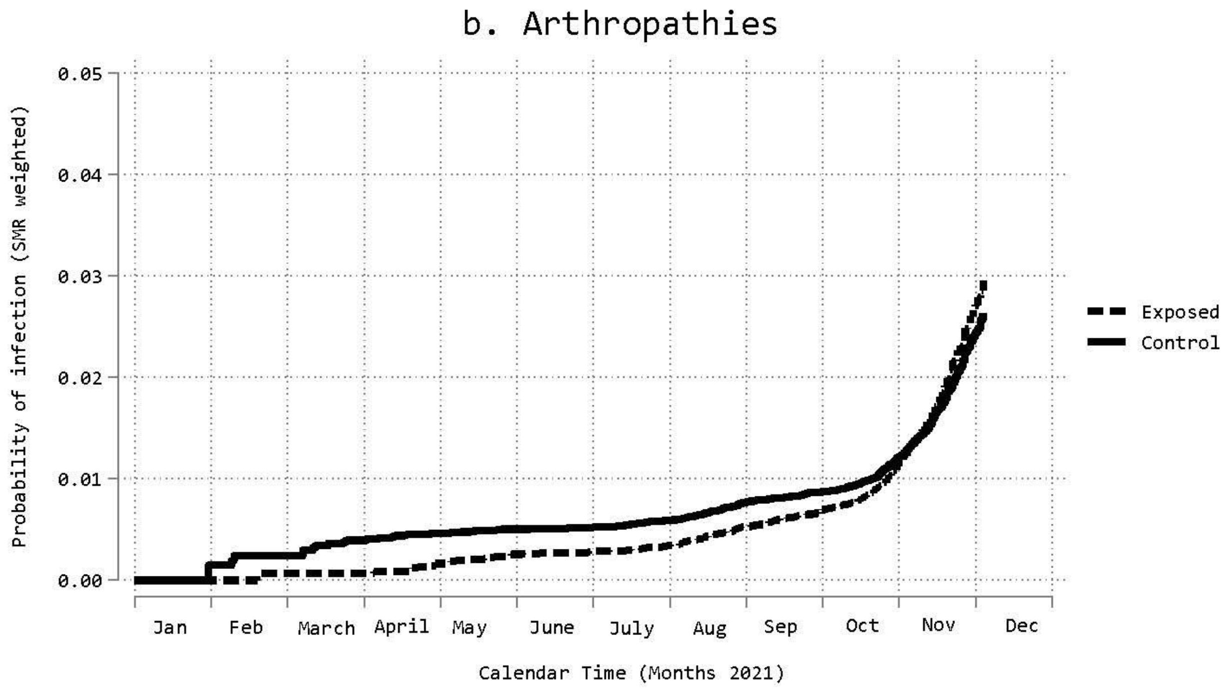

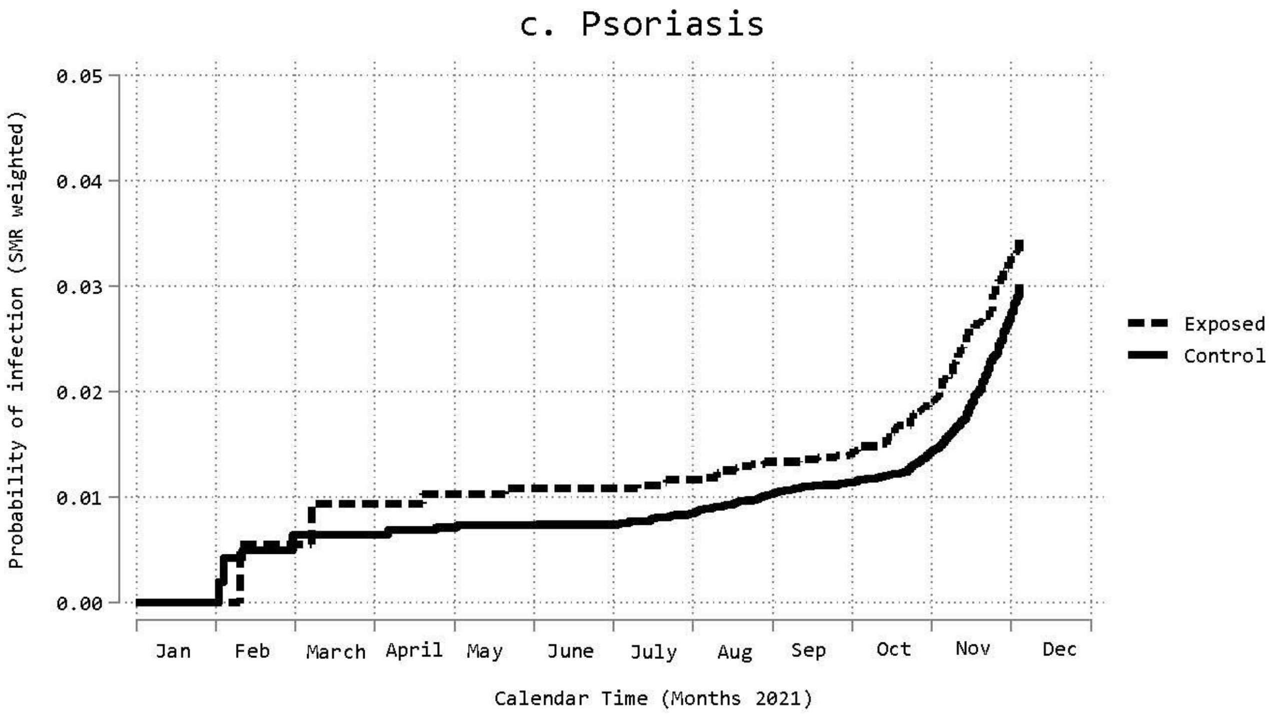

For peer review only

STROBE Statement—Checklist of items that should be included in reports of ***cohort studies***

The Impact of Immunosuppressive Therapy on SARS-CoV-2 mRNA Vaccine Effectiveness in Patients with Immune-mediated Inflammatory Diseases: A Nationwide Danish Cohort Study

	Item No	Recommendation	Page No
**Title and abstract**	1	(*a*) Indicate the study's design with a commonly used term in the title or the abstract	3
		(*b*) Provide in the abstract an informative and balanced summary of what was done and what was found	3
**Introduction**			
Background/rationale	2	Explain the scientific background and rationale for the investigation being reported	5
Objectives	3	State specific objectives, including any prespecified hypotheses	5, 6
**Methods**			
Study design	4	Present key elements of study design early in the paper	6-8
Setting	5	Describe the setting, locations, and relevant dates, including periods of recruitment, exposure, follow-up, and data collection	6, 7 & Fig.1
6-10Participants	6	(*a*) Give the eligibility criteria, and the sources and methods of selection of participants. Describe methods of follow-up	6-8 &
		(*b*) For matched studies, give matching criteria and number of exposed and unexposed	Fig.1
Variables	7	Clearly define all outcomes, exposures, predictors, potential confounders, and effect modifiers. Give diagnostic criteria, if applicable	7,8
Data sources/ measurement	8*	For each variable of interest, give sources of data and details of methods of assessment (measurement). Describe comparability of assessment methods if there is more than one group	7,8
Bias	9	Describe any efforts to address potential sources of bias	6-9
Study size	10	Explain how the study size was arrived at	Fig. 1
Quantitative variables	11	Explain how quantitative variables were handled in the analyses. If applicable, describe which groupings were chosen and why	7-9
Statistical methods	12	(*a*) Describe all statistical methods, including those used to control for confounding	7-9
		(*b*) Describe any methods used to examine subgroups and interactions	
		(*c*) Explain how missing data were addressed	
		(*d*) If applicable, explain how loss to follow-up was addressed	
		(*e*) Describe any sensitivity analyses	
**Results**			
Participants	13*	(a) Report numbers of individuals at each stage of study—eg numbers potentially eligible, examined for eligibility, confirmed eligible, included in the study, completing follow-up, and analysed	9,10 & Table 1
		(b) Give reasons for non-participation at each stage	
		(c) Consider use of a flow diagram	
Descriptive data	14*	(a) Give characteristics of study participants (eg demographic, clinical, social) and information on exposures and potential confounders	9,10

For peer review only - http://bmjopen.bmj.com/site/about/guidelines.xhtml

		(b) Indicate number of participants with missing data for each variable of interest<br>(c) Summarise follow-up time (eg, average and total amount)	
Outcome data	15*	Report numbers of outcome events or summary measures over time	9 Fig.2-4
Main results	16	(*a*) Give unadjusted estimates and, if applicable, confounder-adjusted estimates and their precision (eg, 95% confidence interval). Make clear which confounders were adjusted for and why they were included<br>(*b*) Report category boundaries when continuous variables were categorized<br>(*c*) If relevant, consider translating estimates of relative risk into absolute risk for a meaningful period	9,10 & Fig. 2-4
Other analyses	17	Report other analyses done—e.g. analyses of subgroups and interactions, and sensitivity analyses	10 Fig.4
**Discussion**			
Key results	18	Summarise key results with reference to study objectives	10
Limitations	19	Discuss limitations of the study, taking into account sources of potential bias or imprecision. Discuss both direction and magnitude of any potential bias	13
Interpretation	20	Give a cautious overall interpretation of results considering objectives, limitations, multiplicity of analyses, results from similar studies, and other relevant evidence	13
Generalisability	21	Discuss the generalisability (external validity) of the study results	11,12
**Other information**			
Funding	22	Give the source of funding and the role of the funders for the present study and, if applicable, for the original study on which the present article is based	Abstract & 14

*Give information separately for exposed and unexposed groups.

Note: An Explanation and Elaboration article discusses each checklist item and gives methodological background and published examples of transparent reporting. The STROBE checklist is best used in conjunction with this article (freely available on the Web sites of PLoS Medicine at http://www.plosmedicine.org/, Annals of Internal Medicine at http://www.annals.org/, and Epidemiology at http://www.epidem.com/). Information on the STROBE Initiative is available at http://www.strobe-statement.org.

For peer review only

BMJ Open

The Impact of Immunosuppressive Therapy on SARS-CoV-2 mRNA Vaccine Effectiveness in Patients with Immune-mediated Inflammatory Diseases: A Danish Nationwide Cohort Study

Journal:	*BMJ Open*
Manuscript ID	bmjopen-2023-077408.R2
Article Type:	Original research
Date Submitted by the Author:	05-Feb-2024
Complete List of Authors:	Elmahdi, Rahma; Aalborg Universitet, Department of Clinical Medicine ; Aalborg University Hospital, Department of Gastroenterology and Hepatology<br>Ward, Daniel; Aalborg Universitet, Department of Clinical Medicine<br>Ernst, Martin; University of Southern Denmark, Clinical Pharmacology and Pharmacy, Department of Public Health<br>Poulsen, Gry; Aalborg University, Department of Clinical Medicine<br>Hallas, Jesper; University of Southern Denmark, Clinical pharmacology and pharmacy<br>Pottegard, Anton; University of Southern Denmark, Clinical Pharmacology and Pharmacy, Department of Public Health<br>Jess, Tine; Aalborg Universitet, Department of Clinical Medicine; Aalborg University Hospital, Department of Gastroenterology and Hepatology
**Primary Subject Heading**:	Epidemiology
Secondary Subject Heading:	Gastroenterology and hepatology, Infectious diseases
Keywords:	COVID-19, EPIDEMIOLOGY, Inflammatory bowel disease < GASTROENTEROLOGY, RHEUMATOLOGY, Psoriasis < DERMATOLOGY

SCHOLARONE™
Manuscripts

I, the Submitting Author has the right to grant and does grant on behalf of all authors of the Work (as defined in the below author licence), an exclusive licence and/or a non-exclusive licence for contributions from authors who are: i) UK Crown employees; ii) where BMJ has agreed a CC-BY licence shall apply, and/or iii) in accordance with the terms applicable for US Federal Government officers or employees acting as part of their official duties; on a worldwide, perpetual, irrevocable, royalty-free basis to BMJ Publishing Group Ltd ("BMJ") its licensees and where the relevant Journal is co-owned by BMJ to the co-owners of the Journal, to publish the Work in this journal and any other BMJ products and to exploit all rights, as set out in our licence.

The Submitting Author accepts and understands that any supply made under these terms is made by BMJ to the Submitting Author unless you are acting as an employee on behalf of your employer or a postgraduate student of an affiliated institution which is paying any applicable article publishing charge ("APC") for Open Access articles. Where the Submitting Author wishes to make the Work available on an Open Access basis (and intends to pay the relevant APC), the terms of reuse of such Open Access shall be governed by a Creative Commons licence – details of these licences and which Creative Commons licence will apply to this Work are set out in our licence referred to above.

Other than as permitted in any relevant BMJ Author's Self Archiving Policies, I confirm this Work has not been accepted for publication elsewhere, is not being considered for publication elsewhere and does not duplicate material already published. I confirm all authors consent to publication of this Work and authorise the granting of this licence.

For peer review only

The Impact of Immunosuppressive Therapy on SARS-CoV-2 mRNA Vaccine Effectiveness in Patients with Immune-mediated Inflammatory Diseases: A Danish Nationwide Cohort Study

Short Title: COVID-19 Vaccination in Immunosuppressed IMID Patients

Rahma Elmahdi[*1,2] (0000-0002-7013-9548), Daniel Ward[*1] (0000-0003-0405-7250), Martin Thomsen Ernst[3] (0000-0001-9003-3823), Gry Juul Poulsen[1] (0000-0003-2744-2469), Jesper Hallas[3] (0000-0002-8097-8708), Anton Pottegård[3] (0000-0001-9314-5679), Tine Jess[1,2] (0000-0002-4391-7332)

[*] Co-first author

Rahma Elmahdi, Associate Professor, Center for the Molecular Prediction of Inflammatory Bowel Disease (PREDICT), Department of Clinical Medicine, Aalborg University, Denmark (1)

Department of Gastroenterology and Hepatology, Aalborg University Hospital, Aalborg, Denmark (2)

Daniel Ward, Associate Research Fellow, Center for the Molecular Prediction of Inflammatory Bowel Disease (PREDICT), Department of Clinical Medicine, Aalborg University, Denmark (1)

Martin Ernst Thomsen, Data Manager, Department of Public Health, Clinical Pharmacology, Pharmacy and Environmental Medicine, University of Southern Denmark, J.B. Winsløws Vej 19, 5000 Odense, Denmark (3)

Gry Juul Poulsen, Chief Statistician, Center for the Molecular Prediction of Inflammatory Bowel Disease (PREDICT), Department of Clinical Medicine, Aalborg University, Denmark (1)

Jesper Hallas, Professor, Research Unit of Clinical Pharmacology, University of Southern Denmark, J.B. Winsløws Vej 9, 5000 Odense, Denmark (3)

Anton Pottegård, Professor, Department of Clinical Pharmacology and Pharmacy, University of Southern Denmark, J.B. Winsløws Vej 9, 5000 Odense, Denmark (3)

For peer review only

Tine Jess, Professor, Center for the Molecular Prediction of Inflammatory Bowel Disease (PREDICT), Department of Clinical Medicine, Aalborg University, Denmark (1)

Professor, Department of Gastroenterology and Hepatology, Aalborg University Hospital, Aalborg, Denmark (2)

Correspondence: Dr Rahma Elmahdi, National Center of Excellence for Molecular Prediction of Inflammatory Bowel Disease, PREDICT, Aalborg University, A. C. Meyers Vænge 15, DK-2450 Copenhagen, Denmark. E-mail: rahmae@dcm.aau.dk

Key words: Immune-mediated inflammatory disease; Cohort study; SARS-CoV-2; Vaccination; Immunosuppressives

Word count: Abstract (excluding Strengths and limitations of this study), 288; manuscript body (excluding tables, figure legends and references), 3840

For peer review only

ABSTRACT

Objective: Patients receiving immunosuppressives have been excluded from trials for SARS-CoV-2 vaccine efficacy. Investigation of immunosuppressants' impact on effectiveness of vaccines, particularly in patients with immune-mediated inflammatory diseases (IMID), are therefore required.

Design: We performed a nationwide cohort study to assess the risk of COVID-19 infection in vaccinated IMID patients exposed to immunosuppressives compared to IMID unexposed to immunosuppressives. Exposure to immunosuppressives in the 120 days before receiving the second SARS-CoV-2 mRNA vaccination was assessed. Patients were followed from date of second vaccination and weighted Cox models were used to estimate the risk of infection associated with immunosuppressives. Secondary outcomes included hospitalization and death associated with a positive SARS-CoV-2 test. Risk of infection by immunosuppressant drug class was also analysed.

Setting: This study used population-representative data from Danish national health registries in the period from 1st January to 30th November 2021.

Results: Overall, 152,440 patients were followed over 19,341 person-years. Immunosuppressants were associated with a significantly increased risk of infection across IMID (HR: 1.4, 95% CI: 1.2, 1.5), in inflammatory bowel disease (IBD) (HR: 1.6, 95% CI: 1.4, 1.9) and arthropathy (HR:1.3, 95% CI: 1.1, 1.4) but not psoriasis (HR: 1.1, 95% CI: 0.88, 1.4). Immunosuppressants were also associated with an increased risk of hospitalization across IMID (HR: 1.4, 95% CI: 1.0, 2.0), particularly in IBD (HR: 2.1, 95% CI: 1.0, 4.1). No significantly increased risk of death in immunosuppressant exposed patients was identified. Analyses by immunosuppressant drug class showed increased COVID-19

For peer review only - http://bmjopen.bmj.com/site/about/guidelines.xhtml

infection and hospitalization with anti-TNF, systemic corticosteroid, and rituximab and other immunosuppressants in vaccinated IMID patients.

Conclusion: Immunosuppressive therapies reduced effectiveness of mRNA SARS-CoV-2 vaccination against infection and hospitalization in IMID patients. Anti-TNF, systemic corticosteroids, and rituximab and other immunosuppressants were particularly associated with these risks.

Strengths and limitations of this study

- Use of a non-selected, population representative cohort to source inflammatory and immune mediated disease (IMID) patients.
- Inclusion of a total of 184,346 immunosuppressive exposed IMID patients and 152,440 propensity score matched, unexposed controls.
- Complete vaccination, and immunosuppressive treatment exposure data along with complete infection, hospitalization, and death outcome data with no loss to follow-up.
- Lack of individual level data on level of exposure to infection, such as shielding behaviour.

For peer review only - http://bmjopen.bmj.com/site/about/guidelines.xhtml

INTRODUCTION

SARS-CoV-2 mRNA vaccines Comirnaty® (Pfizer-BioNTech) and Spikevax® (Moderna) were found to be efficacious in clinical trials prior to authorisation, and by December 2022 over 758 million doses of Pfizer-BioNTech and 164 million doses of Moderna were administered in the European Union [1]. Pre-marketing trials excluded individuals considered at risk of immunocompromise, including those receiving immunosuppressive therapies [2,3], therefore there remains a paucity of data on the real-world effectiveness of SARS-CoV-2 vaccines in patients treated with immunosuppressive drugs.

Research published early in the SARS-CoV-2 epidemic suggested that, in the absence of vaccination, some types of immunosuppressives including rituximab, sulfasalazine, and corticosteroids are associated with an increased risk of severe outcomes in COVID-19 infection [4–8]. Immune-mediated inflammatory diseases (IMID), including inflammatory bowel disease, inflammatory arthropathy, and psoriasis have themselves independently been associated with lower serological responses to SARS-CoV-2 vaccination than in healthy controls [9]. Immunosuppressants are key therapies in IMID, so patients with IMID may be at increased risk of infection and severe outcomes of COVID-19 infection both due to the natural history of the diseases and the therapies used to treat them. Even in the context of second vaccination against SARS-CoV-2, exposure to immunosuppressives has been associated with a significantly poorer humoral response; lower than that which is required to confer immunity against infection and severe outcomes of COVID-19 infection in patients treated with immunosuppressive therapies [10–12].

For peer review only - http://bmjopen.bmj.com/site/about/guidelines.xhtml

It is therefore important to investigate the impact of immunosuppressants on SARS-CoV-2 vaccine effectiveness, while controlling for the underlying disease-indicating treatment, and other confounders that may impact vaccine effectiveness.

The real-world effectiveness of SARS-CoV-2 mRNA vaccinations against COVID-19 infection and associated outcomes such as hospitalization or death, in the context of immunosuppressive therapy exposure among IMID patients has not yet been investigated. The aim of this study was to use Danish nationwide population-based data to assess the impact of immunosuppressive exposure on the risk of COVID-19 infection in three cohorts of vaccinated IMID patients.

MATERIALS AND METHODS

Data sources

We conducted a nationwide cohort study using the Danish COVID-19 cohort [13], based on data from the Danish Microbiology Registry [14], which includes individual-level information on vaccine type, dose, and date of administration; SARS-CoV-2 test type and date administered. This data was linked at the individual level to both the Danish National Patient Registry [15] and the Danish National Prescription Registry [16] using a unique Danish Civil Registration number (assigned to all individuals residing in Denmark). The Danish National Patient Registry, a register of hospital activities, includes medical diagnoses coded using International Classification of Disease (ICD-10), and medical procedures and prescriptions including treatment with intravenous medications. The Danish National Prescription Registry contains information on prescriptions dispensed at all community retail pharmacies, including date of dispensing, tablet strength, and pack sizes. Ethics board review is not required for epidemiological research using nationwide registers in Denmark as data is pseudonymised and does not involve patients.

For peer review only - http://bmjopen.bmj.com/site/about/guidelines.xhtml

Population, follow-up, and outcomes

The IMID cohort comprised of three disease specific cohorts of all patients diagnosed with inflammatory bowel disease, including Crohn's disease and ulcerative colitis (IBD; ICD-10: K50, K51), inflammatory arthropathy, including ankylosing spondylitis, other inflammatory spondylopathies, seropositive rheumatoid arthritis, other rheumatoid arthritis, and psoriatic and enteropathic arthropathy (ICD-10: M45, M46, M05, M06, M07) or psoriasis (ICD-10: L40) in Denmark, who had received two doses of SARS-CoV-2 mRNA (Pfizer-BioNTech or Moderna) vaccine. Exclusion criteria were not receiving two doses of SARS-CoV-2 mRNA vaccine and migration prior to receipt of second vaccination. Patients with more than one of these IMID diagnoses were included in only one cohort, with IBD taking precedence, then inflammatory arthropathy, finally psoriasis. Therefore, only patients with psoriasis and neither an IBD nor an inflammatory arthropathy diagnosis were included in the psoriasis cohort. This order was preferred as extent of organ-specific disease likely determines the dose for immunosuppressive therapy. Registration of IMID is based on clinical diagnoses, in line with national and international guidelines, such as ECCO-ESGAR guidelines for IBD diagnosis [17,18].

Patients were followed from the date of administration of second mRNA vaccine (the index date) after 1 January 2021. The primary outcome was a positive SARS-CoV-2 PCR test in the observation period. Secondary outcomes were hospitalization between 7 days prior to and up to 28 days after a positive test or death within 60 days of a positive test (both recorded in Danish National Patient Register). Follow-up was censored at administration of a third vaccination, emigration, death (in the absence of a positive SARS-CoV-2 test) or the end of the study period, 30th November 2021 (Figure 1), as prevalence of the omicron variant became substantial after 28th November 2021 [19]. As the

For peer review only - http://bmjopen.bmj.com/site/about/guidelines.xhtml

registers are complete for the presence of patients up to emigration or death, therefore all patients are retained until the event and there is no missing data.

Patient and Public Involvement

None.

Exposures

The exposures for this study include dispensed prescriptions or hospital administration of an immunosuppressive in the 120 days preceding the index date (date of administration of second vaccination). Immunosuppressants included selective immunosuppressants, anti-TNFs, interleukin inhibitors, calcineurin inhibitors, corticosteroids, rituximab and other immunosuppressants (see Supplementary Table 1 for complete list and ACT codes for immunosuppressants). The 120-day exposure window is chosen to cover the largest pack sizes of prescriptions which can contain medications for up to 120 days. A minimum daily dose of corticosteroids equivalent to 7.5 mg prednisolone per day was estimated as the entire dispensed quantity of corticosteroids during a sequence of prescriptions (within the 120-day exposure period) divided by the number of days from the first prescription to the index date (Supplementary Table 2). Unexposed IMID patients were defined as those with a diagnosis of one of the three IMID, who had not received an immunosuppressive in the 120 days preceding the index date, and those receiving <7.5 mg prednisolone-equivalent average per day.

Statistical models

Covariates included age (handled as a continuous covariate), sex, comorbidities (cardiovascular disease, pulmonary disease, liver disease, kidney disease, other gastrointestinal diseases, skin disease,

For peer review only - http://bmjopen.bmj.com/site/about/guidelines.xhtml

and musculoskeletal disease), medications (cardiovascular drugs, antibiotics, oral anticoagulants, diabetes, and chronic airway disease medications). Testing frequency varied during the period studied due to changes in national and international guidelines and travel restrictions, along with the background prevalence of SARS-CoV-2, which could introduce bias in case detection. We therefore adjusted for individual testing frequency by including number of tests in the month preceding index date as a continuous covariate. Further, covariates specific to each IMID were included separately for each cohort. For the IBD cohort this included any IBD-related hospital admissions in the previous year, Crohn's disease, ulcerative colitis, 5-ASA/sulfasalazine, budesonide, IBD-related procedures, and endoscopy of the gastrointestinal tract (see Supplementary Table 3 for complete list of IMID cohort specific covariates).

To balance the covariates in the exposed and unexposed groups, we fitted propensity score (PS) models for each IMID cohort separately. Propensity scores were calculated using logistic regression for the probability of exposure (treatment with immunosuppressives) conditional on the covariates defined above [20]. We subsequently implemented the PS using standardized mortality ratio (SMR) weights (with trimming of subjects with extreme weights beyond 1st and 99th centiles). We assessed the distribution of covariates with standardized differences before and after PS weighting.

We used weighted Cox proportional-hazards regression models [21] to estimate risk of the COVID-19 infection in IMID patients exposed to immunosuppressive therapy compared to unexposed patients for each disease cohort separately. We used calendar time as the underlying time scale to account for period effects on the risk of the outcomes which may relate to varying infection prevalence and patient characteristics as patients vulnerable to severe outcomes were vaccinated earlier in the year.

For peer review only - http://bmjopen.bmj.com/site/about/guidelines.xhtml

We performed secondary analyses to further delineate the impact of immunosuppressives on vaccine effectiveness over time by stratifying time since vaccination into the following intervals: 0-3 months, 3-6 months, 6-11 months. This not only allowed us to capture the period effects of COVID-19 infection risk earlier and later in the pandemic period but also allowed us to assess the impact of censoring at different time points in the follow-up period.

We then undertook Fixed Effects Model meta-analysis to calculate the pooled HR of infection, hospitalization, and death for IBD, arthropathy and psoriasis cohorts as overall risk in immunosuppressive exposed IMID by COVID-19 outcome, and the HR of infection during 0-3, 3-6, and 6-11 months of follow-up period, as overall risk in immunosuppressive exposed IMID by period.

Finally, we also undertook drug specific analysis for risk of COVID-19 infection by immunosuppressive drug class. In this analysis, patients receiving multiple immunosuppressive treatments were treated as independently exposed to each drug class. To account for the potential impact of immunosuppressants commonly prescribed in a weaning dose, which would not be captured using the definition of ≥7.5 mg dose equivalent per day, we undertook a sensitivity analysis to assess whether having any prescription for systemic corticosteroids over the 120-day period before the index date had an impact on the risk of infection, hospitalization, or death for those exposed to this class of immunosuppressants.

RESULTS

A total of 184,346 patients diagnosed with IBD, arthropathy or psoriasis were identified. After exclusion of patients not receiving two doses of SARS-CoV-2 mRNA vaccine, migration prior to receipt of second vaccination, and trimming of those with extreme propensity scores a total 152,440

For peer review only - http://bmjopen.bmj.com/site/about/guidelines.xhtml

patients were included, contributing a total 19,341 person-years of follow-up. During the 120-day exposure assessment period, 39,765 IMID patients received immunosuppressive treatment (10,480 IBD, 24,261 arthropathy, and 5,023 psoriasis), and 112,629 IMID patients (46,980 IBD, 44,650 arthropathy, and 201,999 psoriasis) patients did not. A total of 11 exposed and 55 unexposed IMID patients are censored from overall analysis due to migration or inclusion on the date of study end (therefore contributing no follow-up time). One arthropathy patient in the exposed group, and 10 IBD or psoriasis patients in the unexposed group are excluded from the infection analysis due to positive test on the date of study entry, these are subsequently included in the analysis for risk of hospitalization or death following COVID-19 infection (Figure 1; Supplementary Table 4). Following application of SMR weighting, the cohorts were balanced on the included covariates (see Table 1 for covariate prevalence and standardised differences).

A total of 866 (2.2%) COVID-19 infections were recorded among immunosuppressive exposed IMID patients during the follow-up period compared with 2,077 (1.8%) for unexposed IMID patients. This gave an incidence rate of 55 (49-61) per 1,000 person-years in immunosuppressive exposed IBD patients compared with 43 (40-45) in unexposed IBD patients, 40 (37-44) per 1,000 person-years for immunosuppressive exposed arthropathy patients compared with 38 (35-41) in unexposed arthropathy patients, and 45 (37-55) per 1,000 person-years for immunosuppressive exposed psoriasis patients compared with 42 (38-46) per 1,000 person-years in unexposed psoriasis patients.

A significantly increased weighted hazard for infection among exposed patients was seen for both IBD (HR: 1.6, 95% CI: 1.4, 1.9) and arthropathy (HR: 1.3, 95% CI: 1.1, 1.4) cohorts but not for the psoriasis cohort (HR: 1.1, 95 % CI: 0.88, 1.4). Meta-analysis of the three IMID cohorts showed a pooled HR for COVID-19 infection in exposed patients of 1.4 (95% CI: 1.2, 1.5; Figure 2). Fewer

For peer review only - http://bmjopen.bmj.com/site/about/guidelines.xhtml

than 57 exposed and 122 unexposed IMID patients were hospitalized with COVID-19 infection during the follow-up period, which corresponded to a significantly increased risk of hospitalization overall for immunosuppressive exposed IMID patients (pooled HR: 1.4, 95% CI: 1.0, 2.0). This increased risk of overall hospitalization is largely due to the contribution of the risk of hospitalization in IBD patients 2.05 (95% CI: 1.03-4.07). Less than five immunosuppressive exposed IBD patients died in the 60 days following a COVID-19 diagnosis compared to six unexposed IBD patients. Six patients in the arthropathy cohort exposed to immunosuppressives compared with 13 unexposed arthropathy patients died in the 60 days following a COVID-19 diagnosis and fewer than five patients with a psoriasis diagnosis, either immunosuppressive exposed or unexposed died with a COVID-19 diagnosis. These did not correspond to a significantly increased risk of death among exposed patients in any of the three cohorts or overall (pooled HR: 0.92, 95% CI: 0.38, 2.2; Figure 2).

In the first 0-3 months following vaccination, both IBD and arthropathy immunosuppressive exposed patients had a significantly increased risk of COVID-19 infection (HR: 1.5, 95% CI: 1.1, 2.2 and HR: 1.3, 95% CI: 1.0, 1.7, respectively; see Supplementary Table 5). Most COVID-19 infections following second vaccination occurred in the 3–6-month period with a total of 470 infections in exposed IMID patients compared with 1,241 unexposed IMID patients. Only exposed arthropathy patients had a significantly increased risk of infection compared to their unexposed counterparts during this period however (HR: 1.2, 95% CI: 1.1, 1.4). The highest incidence rate of COVID-19 infection following second vaccination was seen in the 6–11-month period for both immunosuppressive exposed and unexposed IMID patients and risk of infection during this period was only increased among exposed IBD patients (HR: 1.4, 95% CI: 1.1, 1.9). There was however a high rate of censoring among both the immunosuppressive exposed (over 50%) and the unexposed (almost 49%) groups in the 6–11-month period due to receipt of the third SARS-CoV-2 vaccination

For peer review only - http://bmjopen.bmj.com/site/about/guidelines.xhtml

so direct comparison of the risk of infection between time periods is challenging. Kaplan-Meier plots and HR showing probability of infection over the calendar time of follow-up (January-November 2021) are presented in Supplementary Figure 1 and post-hoc analysis for HR for infection by calendar period (January-November 2021) is shown in Supplementary Table 6.

Analysis for risk of infection among IMID cohorts by immunosuppressive drug class exposure showed a significantly increased risk of infection among users of anti-TNF (HR: 1.8, 95% CI: 1.6, 2.0), systemic corticosteroid (HR: 1.2, 95% CI: 1.0,1.5), and rituximab and other immunosuppressant (HR: 1.3, 95% CI: 1.1, 1.4; Figure 3). No other immunosuppressant was significantly associated with COVID-19 infection following second vaccination. Anti-TNF and systemic corticosteroid exposure were also associated with an increased risk of COVID-19 associated hospitalization (HR 1.8, 95% CI 1.0, 3.3 and HR 1.8, 95% CI 1.0,3.0, respectively; Figure 4). No immunosuppressive drug class was associated with death among IMID patients following receipt of second vaccination (Supplementary Table 7). Sensitivity analysis assessing outcomes following any systemic corticosteroid exposure in the 120-day period prior to the index date showed no significant difference in risk of infection (crude HR: 1.2, 95% CI: 1.0, 1.4; adjusted HR: 1.1, 95% CI: 0.95, 1.3) or death (crude HR: 3.1, 95% CI: 1.4, 7.0; adjusted HR: 1.9, 95% CI: 0.77, 4.7). However, risk of hospitalization following infection was significantly increased in those ever exposed to systemic corticosteroids in the 120-day period prior to receipt of second vaccination (crude HR: 2.8, 95% CI: 1.9, 4.1; adjusted HR: 2.1, 95% CI: 1.4, 3.2), showing similar results compared with analysis restricting to a ≥7.5 mg daily equivalent dose.

DISCUSSION

For peer review only - http://bmjopen.bmj.com/site/about/guidelines.xhtml

In this large nationwide cohort study of three IMID patient cohorts, we identified a total of 39,756 immunosuppressive exposed patients matched to 112,629 immunosuppressive unexposed IMID patients to investigate the risk of COVID-19 infection, hospitalization, and death among IBD, arthropathy and psoriasis patients following second SARS-CoV-2 vaccination. Meta-analysis of the three cohorts showed an overall 35% increased risk of infection and, 42% increased risk of COVID-19 associated hospitalization in immunosuppressive exposed compared to immunosuppressive unexposed IMID patients.

Mortality was not significantly increased in immunosuppressive exposed patients and these events were rare in both groups. Drug class analysis showed anti-TNF, systemic corticosteroid, and rituximab and other immunosuppressant exposure was significantly associated with both increased risk of COVID-19 infection and hospitalization following second vaccination in immunosuppressive exposed compared to unexposed IMID patients.

We attempted to ascertain the effectiveness of SARS-CoV-2 mRNA vaccination among IMID patients exposed to immunosuppressive therapies, while controlling for the severity of the underlying disease indicating immunosuppressive treatment with a propensity score model. We found that immunosuppressants were associated with an increased risk of infection, likely due to the impact of immunosuppressive medication on vaccination against COVID-19 infection. This is particularly seen in IBD (HR:1.6, 95% CI: 1.4-1.9) but is also present in arthropathy (HR: 1.3, 95% CI:1.1, 1.4) patients and, to a lesser extent, in psoriasis (HR: 1.1, 95% CI: 0.88, 1.4). Immunosuppressive exposed psoriasis patients showed no increased infection risk compared to their unexposed counterparts. Similarly, when assessing risk of hospitalization following vaccination by immunosuppressive exposure, we find a significantly increased risk in IBD patients (HR: 2.1, 95% CI 1.0,4,1), which is

For peer review only - http://bmjopen.bmj.com/site/about/guidelines.xhtml

not observed in arthropathy (HR: 1.3, 95% CI: 0.9, 2.0) or psoriasis (HR: 0.6, 95% CI: 0.1, 2.5). The poorer outcomes observed in immunosuppressive exposed IBD patients is in keeping with wider findings. In a meta-analysis of serological response to SARS-CoV-2 vaccination among IMID treated patients, IBD patients were found to have a significantly lower response to first mRNA vaccination dose than rheumatoid patients (response rate: 0.49, 95% CI: 0.32-0.66 and 0.78, 95% CI: 0.67-0.86, respectively) [22]. This may be due to the more extensive disease seen in typical IBD patients, which often necessitates higher doses of immunosuppressive therapies, over longer periods to achieve disease remission than that required for psoriasis or arthropathy [23–25]. However, the difference in increased risk of infection and hospitalization in immunosuppressive exposed IBD compared with unexposed IBD patients is similar to the other IMID cohorts in this study, with overall pooled IMID cohort meta-analysis showing a significantly increased risk for both these outcomes. These findings indicate a general trend towards poorer outcomes in immunosuppressive exposed patients regardless of IMID cohort.

Reassuringly, immunosuppressant exposure was not associated with increased risk of death due to COVID-19 in any of the IMID patient cohorts, suggesting immunosuppressants do not reduce the effectiveness of vaccination in preventing this important outcome. However, caution should be exercised in the interpretation of this finding as deaths were recorded in either immunosuppressive exposed or unexposed patients and this may be due to the relatively short follow-up period of 11-months in this study. Although Kaplan-Meier plots for risk of infection may appear in contradiction to the overall findings of the primary analysis (with apparent increased rate of COVID infection in the unexposed IMID population in the first 7 months of follow-up), the findings from post-hoc Cox regression analysis by calendar period shows that the difference between the groups, reflected in the

For peer review only - http://bmjopen.bmj.com/site/about/guidelines.xhtml

overall HR, only becomes apparent in the final 3 months of follow-up as the majority of cases of COVID are seen in this period.

Meta-analysis showed an overall increased risk of COVID-19 infection among exposed IMID patients during the 0-3 and 3-6-month period only. Only exposed arthropathy patients showed a significantly increased risk of infection in the 3–6-month period. Although this might be interpreted as waning immunity, it is important to note that half of the baseline IMID cohort were censored, largely due to receipt of third vaccination, therefore the remaining population likely differed substantially from the initial cohort. Hence this interpretation of period specific risk estimates should be made tentatively [26]. Although the three periods are not directly comparable, it is likely that our observation of an increased risk of infection in exposed patients in the 0-3- and 3-6-month period reflects a true risk, as is observed in the risk identified over the total follow-up period.

Treatment with TNF-alpha inhibitors, systemic corticosteroid, and rituximab other immunosuppressants was associated with a significantly increased risk of infection, and TNF-alpha inhibitors and systemic corticosteroids were associated with a significantly increased risk of hospitalization following receipt of second vaccination. This is consistent with previous studies, which suggests that treatment with cytokine inhibitors or B-cell depleting immunosuppressives is related to particularly poor COVID-19 outcomes [27–29], however the association with TNF-alpha inhibitors is novel. Our findings of increased risk of infection and hospitalization, but not death, in sensitivity analysis among IMID patients exposed to systemic corticosteroids is also in keeping with those other studies of unvaccinated IMID cohorts in Denmark [4] and internationally [6,30]. These findings indicate that corticosteroid exposure weakens the protection conferred by vaccination. As glucocorticoids are known to inhibit the breadth of the immune response, including aspects of both

For peer review only

For peer review only - http://bmjopen.bmj.com/site/about/guidelines.xhtml

the cellular and humoral immunity induced by mRNA vaccination, these findings appear to be intuitive [31]. The interaction of IMID, the impact of treatments to control disease and response to vaccination, particularly considering the effects of dose and duration of administration is however complex [32,33]. Further studies directly exploring the effects of vaccination whilst controlling for disease severity and exposure of immunosuppressive drugs by dose and duration would be required to disentangle the association of the different immunosuppressive drug classes with COVID-19 outcomes following vaccination. Such studies would also better inform guidance relating to timelines for SARS-CoV-2 vaccination in relation to the administration of immunosuppressive therapies in IMID patients.

One of the key strengths of this study is that it is large and population-representative, exploring the effectiveness of COVID-19 infection using real-world data from comprehensive, nationwide health registries. Vaccination does not directly correlate with protection from infection and the findings from this work provides important evidence on effectiveness of post-marketing mRNA vaccination in a vulnerable patient group. To our knowledge, this is the first study to assess the effectiveness of SARS-CoV-2 vaccination against COVID-19 infection, hospitalization, and mortality among IMID patients, based on immunosuppressive exposure. Additionally, our use of PS weighted regression models allows us to accurately control for the underlying treatment indicating disease, so we are better able to extrapolate the effects of the drug exposure from the disease itself.

Limitations include not being able to extrapolate in the context of the omicron variant, or subsequent subvariants as we restricted to a period of the pandemic where the delta variant was the dominant circulating strain of COVID-19 to ensure consistency in the assessment of our outcomes. Although it is difficult to define a reliable threshold for which we consider a patient unexposed to

For peer review only - http://bmjopen.bmj.com/site/about/guidelines.xhtml

immunosuppressive medication, it is reassuring that only approximately 12% of our unexposed group had filled a prescription in the 365 days prior to the 120-day exposure window assessed, and that removing the minimum threshold of 7.5 mg of systemic corticosteroids as an exposure requirement did not change our findings. A lack of individual-level data relating to confounders such as smoking behaviour, risk of occupational exposure to COVID-19, socio-economic status and dose of drug therapies could potentially limit our findings. Due to lack of availability of such data, we could not account for shielding behaviour in this analysis. There may also be a residual effect of confounding due to unmeasured disease severity not completely accounted for in our PS model. However, these are unlikely to systemically impact the direction of association or strength of significance identified in the risk of infection due to immunosuppressive exposure observed here. We limited our study to IBD, inflammatory arthropathy, and psoriasis although other IMID exist, because these are commonly treated with immunosuppressives such as anti-TNF.

In conclusion, our findings suggest a reduced effectiveness of mRNA SARS-CoV-2 vaccination against COVID-19 infection and hospitalization in IMID patients receiving immunosuppressive therapies. This risk is particularly seen in IBD and arthropathy patients, and COVID-19 infection is associated with anti-TNF, systemic corticosteroids, and rituximab and other immunosuppressant exposure, while TNF-alpha inhibitors and systemic corticosteroids were associated with a significantly increased risk of hospitalization.

Author Contributions: DW, RE, AP and TJ developed the study protocol. ME undertook primary data analysis with support from GP. DW and RE were responsible for first draft of the manuscript. All authors were responsible for interpretation of results and critical revisions to the final manuscript.

For peer review only

Funding Sources: This work was funded by grants from the Novo Nordisk Foundation (NNF21OC0068631) and the Danish National Research Foundation (DNRF-148).

Conflict of Interest Statement: All authors have none to declare.

Data Sharing Statement: The study was based on data from the Danish National Health registers (https://sundhedsdatastyrelsen.dk). The register data are protected by the Danish Act on Processing of Personal Data and are accessed through application to and approval from the Danish Data Protection Agency and the Danish Health Data Authority. The code is available promptly on request made to the corresponding author

For peer review only

REFERENCES

1 European Medicines Agency. COVID-19 vaccines safety update: 3 August 2022 Rev. 1. Amsterdam: www.ema.europa.eu (accessed 11 Aug 2022).

2 El Sahly HM, Baden LR, Essink B, *et al.* Efficacy of the mRNA-1273 SARS-CoV-2 Vaccine at Completion of Blinded Phase. *N Engl J Med* 2021;**385**:1774–85. doi:10.1056/nejmoa2113017

3 Polack FP, Thomas SJ, Kitchin N, *et al.* Safety and Efficacy of the BNT162b2 mRNA Covid-19 Vaccine. *N Engl J Med* 2020;**383**:2603–15. doi:10.1056/NEJMOA2034577/SUPPL_FILE/NEJMOA2034577_PROTOCOL.PDF

4 Ward D, Gørtz S, Ernst MT, *et al.* The effect of immunosuppressants on the prognosis of SARS-CoV-2 infection. *Eur Respir J* 2022;**59**. doi:10.1183/13993003.00769-2021

5 Gianfrancesco M, Hyrich KL, Al-Adely S, *et al.* Characteristics associated with hospitalisation for COVID-19 in people with rheumatic disease: data from the COVID-19 Global Rheumatology Alliance physician-reported registry. *Ann Rheum Dis* 2020;**79**:859–66. doi:10.1136/ANNRHEUMDIS-2020-217871

6 Brenner EJ, Ungaro RC, Gearry RB, *et al.* Corticosteroids, But Not TNF Antagonists, Are Associated With Adverse COVID-19 Outcomes in Patients With Inflammatory Bowel Diseases: Results From an International Registry. *Gastroenterology* 2020;**159**:481-491.e3. doi:10.1053/J.GASTRO.2020.05.032

7 Salter A, Fox RJ, Newsome SD, *et al.* Outcomes and Risk Factors Associated With SARS-CoV-2 Infection in a North American Registry of Patients With Multiple Sclerosis. *JAMA Neurol* 2021;**78**:699–708. doi:10.1001/JAMANEUROL.2021.0688

8 Strangfeld A, Schäfer M, Gianfrancesco MA, *et al.* Factors associated with COVID-19-related death in people with rheumatic diseases: results from the COVID-19 Global

For peer review only

For peer review only - http://bmjopen.bmj.com/site/about/guidelines.xhtml

Rheumatology Alliance physician-reported registry. *Ann Rheum Dis* 2021;**80**:930–42. doi:10.1136/ANNRHEUMDIS-2020-219498

9 Lee ARY Bin, Wong SY, Chai LYA, *et al.* Efficacy of covid-19 vaccines in immunocompromised patients: systematic review and meta-analysis. *BMJ* 2022;**376**:e068632. doi:10.1136/BMJ-2021-068632

10 Boekel L, Steenhuis M, Hooijberg F, *et al.* Antibody development after COVID-19 vaccination in patients with autoimmune diseases in the Netherlands: a substudy of data from two prospective cohort studies. *Lancet Rheumatol* 2021;**3**:e778–88. doi:10.1016/S2665-9913(21)00222-8

11 Alexander JL, Kennedy NA, Ibraheim H, *et al.* COVID-19 vaccine-induced antibody responses in immunosuppressed patients with inflammatory bowel disease (VIP): a multicentre, prospective, case-control study. *lancet Gastroenterol Hepatol* 2022;**7**:342–52. doi:10.1016/S2468-1253(22)00005-X

12 de Boer SE, Berger SP, van Leer–Buter CC, *et al.* Enhanced Humoral Immune Response After COVID-19 Vaccination in Elderly Kidney Transplant Recipients on Everolimus Versus Mycophenolate Mofetil-containing Immunosuppressive Regimens. *Transplantation* 2022;**106**. doi:10.1097/TP.0000000000004177

13 Pottegård A, Kristensen KB, Reilev M, *et al.* Existing Data Sources in Clinical Epidemiology: The Danish COVID-19 Cohort. *Clin Epidemiol* 2020;**12**:875–81. doi:10.2147/CLEP.S257519

14 Voldstedlund M, Haarh M, Mølbak K. The Danish Microbiology Database (MiBa) 2010 to 2013. *Euro Surveill* 2014;**19**. doi:10.2807/1560-7917.ES2014.19.1.20667

15 Jensen JS, Jensen DH, Grønhøj C, *et al.* Incidence and survival of oropharyngeal cancer in Denmark: a nation-wide, population-based study from 1980 to 2014. *Acta Oncol (Madr)*

For peer review only

For peer review only - http://bmjopen.bmj.com/site/about/guidelines.xhtml

2018;**57**:269–75. doi:10.1080/0284186X.2017.1390251

16 Pottegård A, Schmidt SAJ, Wallach-Kildemoes H, *et al.* Data Resource Profile: The Danish National Prescription Registry. *Int J Epidemiol* 2017;**46**:798. doi:10.1093/IJE/DYW213

17 Maaser C, Sturm A, Vavricka SR, *et al.* ECCO-ESGAR Guideline for Diagnostic Assessment in IBD Part 1: Initial diagnosis, monitoring of known IBD, detection of complications. *J Crohn's Colitis* 2019;**13**:144-164K. doi:10.1093/ECCO-JCC/JJY113

18 Kay J, Upchurch KS. ACR/EULAR 2010 rheumatoid arthritis classification criteria. *Rheumatology* 2012;**51**:vi5–9. doi:10.1093/RHEUMATOLOGY/KES279

19 Espenhain L, Funk T, Overvad M, *et al.* Epidemiological characterisation of the first 785 SARS-CoV-2 Omicron variant cases in Denmark, December 2021. *Euro Surveill* 2021;**26**. doi:10.2807/1560-7917.ES.2021.26.50.2101146

20 Rosenbaum PR, Rubin DB. The central role of the propensity score in observational studies for causal effects. *Biometrika* 1983;**70**:41–55. doi:10.1093/BIOMET/70.1.41

21 Desai RJ, Franklin JM. Alternative approaches for confounding adjustment in observational studies using weighting based on the propensity score: a primer for practitioners. *BMJ* 2019;**367**. doi:10.1136/BMJ.L5657

22 Sakuraba A, Luna A, Micic D. Serologic Response to Coronavirus Disease 2019 (COVID-19) Vaccination in Patients With Immune-Mediated Inflammatory Diseases: A Systematic Review and Meta-analysis. *Gastroenterology* 2022;**162**:88-108.e9. doi:10.1053/J.GASTRO.2021.09.055

23 Singh JA, Guyatt G, Ogdie A, *et al.* 2018 American College of Rheumatology/National Psoriasis Foundation Guideline for the Treatment of Psoriatic Arthritis: *https://doi.org/101177/2475530318812244* 2018;**4**:31–58. doi:10.1177/2475530318812244

24 Larsen L, Jensen MD, Larsen MD, *et al.* The Danish National Registry for Biological

For peer review only

For peer review only - http://bmjopen.bmj.com/site/about/guidelines.xhtml

Therapy in Inflammatory Bowel Disease. *Clin Epidemiol* 2016;**8**:607–12. doi:10.2147/CLEP.S99478

25 Cottone M, Sapienza C, Macaluso FS, *et al.* Psoriasis and Inflammatory Bowel Disease. *Dig Dis* 2019;**37**. doi:10.1159/000500116

26 Hernán MA. The hazards of hazard ratios. *Epidemiology* 2010;**21**:13–5. doi:10.1097/EDE.0B013E3181C1EA43

27 Izadi Z, Brenner EJ, Mahil SK, *et al.* Association Between Tumor Necrosis Factor Inhibitors and the Risk of Hospitalization or Death Among Patients With Immune-Mediated Inflammatory Disease and COVID-19. *JAMA Netw open* 2021;**4**. doi:10.1001/JAMANETWORKOPEN.2021.29639

28 Simon D, Tascilar K, Kleyer A, *et al.* Impact of Cytokine Inhibitor Therapy on the Prevalence, Seroconversion Rate, and Longevity of the Humoral Immune Response Against SARS-CoV-2 in an Unvaccinated Cohort. *Arthritis Rheumatol (Hoboken, NJ)* 2022;**74**:783–90. doi:10.1002/ART.42035

29 Simon D, Tascilar K, Fagni F, *et al.* SARS-CoV-2 vaccination responses in untreated, conventionally treated and anticytokine-treated patients with immune-mediated inflammatory diseases. *Ann Rheum Dis* 2021;**80**:1312–6. doi:10.1136/ANNRHEUMDIS-2021-220461

30 Favalli EG, Bugatti S, Klersy C, *et al.* Impact of corticosteroids and immunosuppressive therapies on symptomatic SARS-CoV-2 infection in a large cohort of patients with chronic inflammatory arthritis. *Arthritis Res Ther* 2020;**22**. doi:10.1186/S13075-020-02395-6

31 McKay LI, Cidlowski JA. Physiologic and Pharmacologic Effects of Corticosteroids. Published Online First: 2003.https://www.ncbi.nlm.nih.gov/books/NBK13780/ (accessed 1 Sep 2022).

32 Rahier JF, Moutschen M, van Gompel A, *et al.* Vaccinations in patients with immune-

For peer review only - http://bmjopen.bmj.com/site/about/guidelines.xhtml

mediated inflammatory diseases. *Rheumatology (Oxford)* 2010;**49**:1815–27. doi:10.1093/RHEUMATOLOGY/KEQ183

33 Croce E, Hatz C, Jonker EF, *et al.* Safety of live vaccinations on immunosuppressive therapy in patients with immune-mediated inflammatory diseases, solid organ transplantation or after bone-marrow transplantation - A systematic review of randomized trials, observational studies and case reports. *Vaccine* 2017;**35**:1216–26. doi:10.1016/J.VACCINE.2017.01.048

For peer review only

For peer review only - http://bmjopen.bmj.com/site/about/guidelines.xhtml

TABLES & FIGURES

Table 1. Characteristics of immune-mediated inflammatory disease patients at baseline and after propensity score weighting, by exposure to immunosuppressive therapy.

	**Baseline IMID Cohort***			**Weighted IMID Cohort**		
	**Unexposed**	**Exposed**	**SD**	**Unexposed**	**Exposed**	**SD**
**Total, n (%)**	112 675 (100)	39 765 (100)	NA	39 524 (100)	39 765 (100)	NA
**Inflammatory bowel disease, n (%)**	47 001 (41.7)	10 480 (26.4)	NA	10 284 (26.0)	10 480 (26.4)	NA
**Arthropathy, n (%)**	44 669 (39.6)	24 261 (61.0)	NA	24 227 (61.3)	24 261 (61.0)	NA
**Psoriasis, n (%)**	21 005 (18.6)	5 024 (12.6)	NA	5 014 (12.7)	5 024 (12.6)	NA
Age, median (IQR)	59 (46-71)	58 (45-71)	0.06	58 (44-71)	58 (45-71)	0.01
Male, n (%)	48 941 (43.4)	17 080 (43.0)	0.01	16 934 (42.8)	17 080 (43.0)	0.00
SARS-CoV-2 test in the previous month, median (IQR)	0 (0-1)	0 (0-1)	0.04	0 (0-1)	0 (0-1)	0.00
**Calendar date of entry 2021, n (%)**						
January to April	27,865 (24.7)	16,689 (42.0)	0.37	10,124 (25.6)	16,689 (42.0)	0.35
May to August	81,682 (72.5)	22,361 (56.2)	0.34	28,211 (71.4)	22,361 (56.2)	0.32
September to November	3,128 (2.8)	715 (1.8)	0.07	1,189 (3.0)	715 (1.8)	0.08
**Comorbidities, n (%)**						
Cardiovascular disease	41 056 (36.4)	14 010 (35.2)	0.03	14 050 (35.5)	14 010 (35.2)	0.01
Pulmonary disease	14 994 (13.3)	5 793 (14.6)	0.04	5796 (14.7)	5 793 (14.6)	0.00
Liver disease	3 630 (3.2)	1 528 (3.8)	0.03	1 525 (3.9)	1 528 (3.8)	0.00
Kidney disease	6 859 (6.1)	2 260 (5.7)	0.02	2 279 (5.8)	2 260 (5.7)	0.00
Other gastrointestinal diseases	21 505 (19.1)	7 086 (17.8)	0.03	6 984 (17.7)	7 086 (17.8)	0.00
Skin disease	9 889 (8.8)	3 748 (9.4)	0.02	3 703 (9.4)	3 748 (9.4)	0.00
Musculoskeletal disease	45 393 (40.3)	16 620 (41.8)	0.03	16 649 (42.1)	16 620 (41.8)	0.01
**Medications, n (%)**						
Cardiovascular drugs	80 529 (71.5)	28 179 (70.9)	0.01	28 054 (71.0)	28 179 (70.9)	0.00
Antibiotics	108 502 (96.3)	38 405 (96.6)	0.02	38 181 (96.6)	38 405 (96.6)	0.00
Oral anticoagulants	11 111 (9.9)	4 022 (10.1)	0.01	4 034 (10.2)	4 022 (10.1)	0.00
Drugs used in diabetes	12 935 (11.5)	4 034 (10.1)	0.04	4 030 (10.2)	4 034 (10.1)	0.00

For peer review only

Drugs for obstructive airway diseases	36 970 (32.8)	13 118 (33.0)	0.00	13 116 (33.2)	13 118 (33.0)	0.00
**IBD-specific treatments, n (%)**						
Any IBD-related hospital admissions in the previous year	564 (1.2)	714 (6.8)	0.12	584 (5.7)	714 (6.8)	0.05
5-ASA/sulfasalazine	12 596 (26.8)	2 845 (27.1)	0.00	2 857 (27.8)	2 845 (27.1)	0.01
Budesonide	646 (1.4)	272 (2.6)	0.02	277 (2.7)	272 (2.6)	0.01
IBD related procedures	18 553 (39.5)	4 551 (43.4)	0.05	4 473 (43.5)	4 551 (43.4)	0.00
Endoscopy of the gastrointestinal tract	7 454 (15.9)	3 702 (35.3)	0.12	3 543 (34.5)	3 702 (35.3)	0.02
**Arthropathy-specific treatments, n (%)**						
Arthropathy-related procedures	13 932 (31.2)	8 415 (34.7)	0.09	8 399 (34.7)	8 415 (34.7)	0.00
Anti-inflammatory and anti-rheumatic drugs	5 678 (12.7)	3 462 (14.3)	0.07	3 480 (14.4)	3 462 (14.3)	0.00
Hydroxychloroquine	540 (1.2)	845 (3.5)	0.15	801 (3.3)	845 (3.5)	0.01
**Psoriasis-specific treatments, n (%)**						
5-ASA/sulfasalazine	2 149 (10.2)	807 (16.1)	0.03	805 (16.1)	807 (16.1)	0.00
Topical corticosteroids	73 (0.3)	42 (0.8)	0.00	39 (0.8)	42 (0.8)	0.01
Anti-psoriatic medication	7 441 (35.4)	1 894 (37.7)	0.01	1 900 (37.9)	1 894 (37.7)	0.00
Topical calcineurin inhibitors	3 731 (17.8)	1 106 (22.0)	0.00	1 110 (22.1)	1 106 (22.0)	0.00
Psoriasis related procedures	542 (2.6)	144 (2.9)	0.00	144 (2.9)	144 (2.9)	0.00
5-ASA/sulfasalazine	2 149 (10.2)	807 (16.1)	0.03	805 (16.1)	807 (16.1)	0.00

Abbreviations: IMID = immune-mediated inflammatory disease; SD = standardized differences; IQR = inter-quartile range; NA = not applicable. *Total cohort numbers prior to trimming.

FIGURE LEGENDS

Figure 1. Flow chart for inclusion into the exposed and unexposed IMID cohort.

Figure 2. Risk of infection, hospitalization and death associated with immunosuppressive exposure from Jan 2021-Nov 2021 in Inflammatory Bowel Disease, Arthropathy, Psoriasis and across all IMID cohorts.

Figure 3. Risk of infection associated with immunosuppressive drug class exposure (Selective Immunosuppressants, Tumour Necrosis Factor Inhibitors, Interleukin Inhibitors, Calcineurin Inhibitors, Systemic Corticosteroids, and Other Immunosuppressants including Rituximab) in IMID patients.

Figure 4. Risk of hospitalization associated with immunosuppressive drug class exposure (Selective Immunosuppressants, Tumour Necrosis Factor Inhibitors, Interleukin Inhibitors, Calcineurin Inhibitors, Systemic Corticosteroids, and Other Immunosuppressants including Rituximab) in IMID patients.

For peer review only - http://bmjopen.bmj.com/site/about/guidelines.xhtml

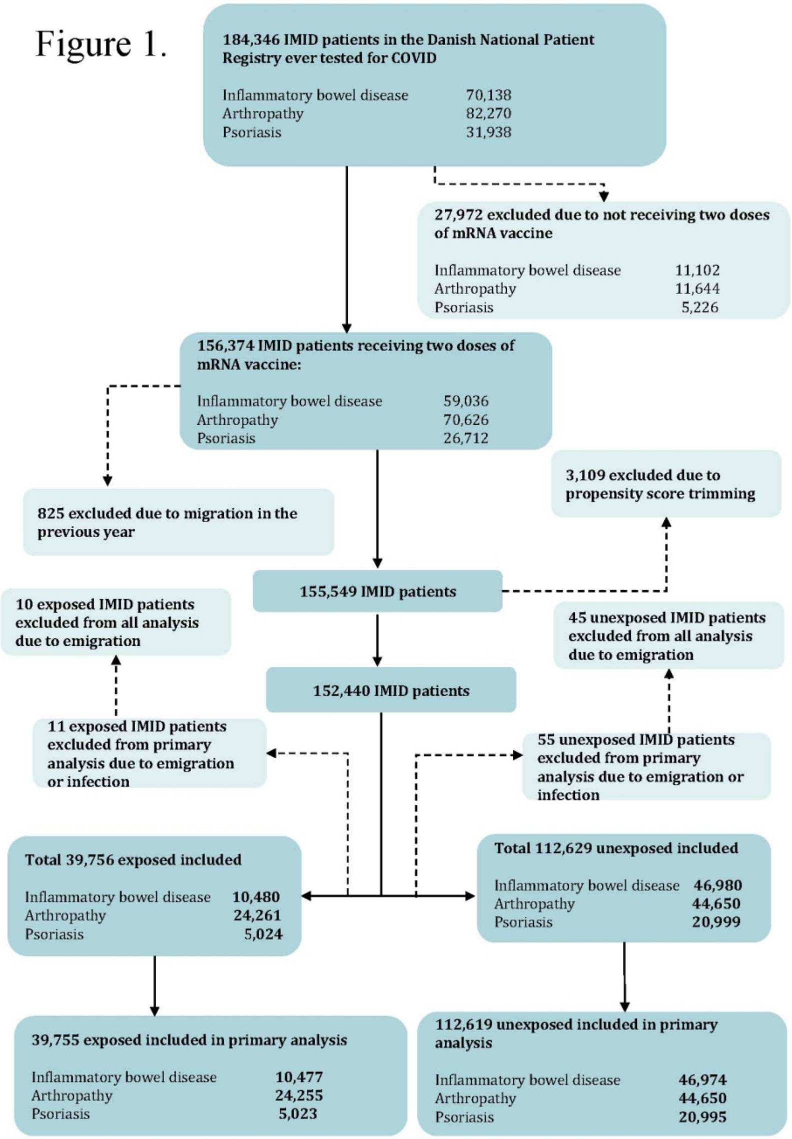

Figure 1. Flow chart for inclusion into the exposed and unexposed IMID cohort.

190x275mm (200 x 200 DPI)

Figure 2.

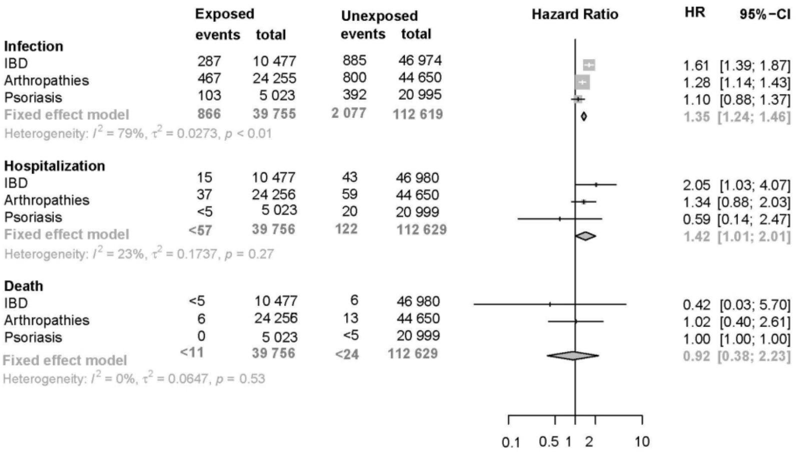

Figure 2. Risk of infection, hospitalization and death associated with immunosuppressive exposure from Jan 2021-Nov 2021 in Inflammatory Bowel Disease, Arthropathy, Psoriasis and across all IMID cohorts.

296x209mm (200 x 200 DPI)

Figure 3.

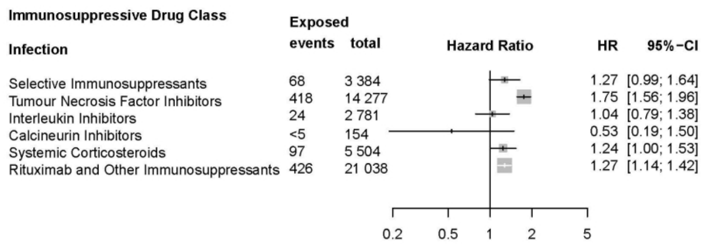

Figure 3. Risk of infection associated with immunosuppressive drug class exposure (Selective Immunosuppressants, Tumour Necrosis Factor Inhibitors, Interleukin Inhibitors, Calcineurin Inhibitors, Systemic Corticosteroids, and Other Immunosuppressants including Rituximab) in IMID patients.

296x209mm (200 x 200 DPI)

Figure 4.

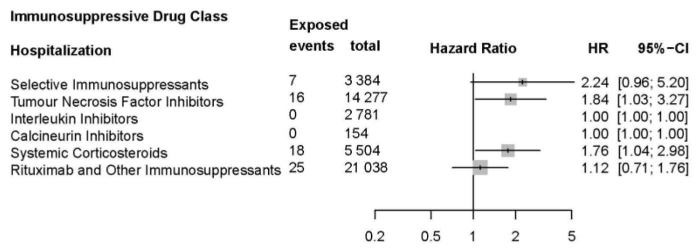

Figure 4. Risk of hospitalization associated with immunosuppressive drug class exposure (Selective Immunosuppressants, Tumour Necrosis Factor Inhibitors, Interleukin Inhibitors, Calcineurin Inhibitors, Systemic Corticosteroids, and Other Immunosuppressants including Rituximab) in IMID patients.

296x209mm (200 x 200 DPI)

The Impact of Immunosuppressive Therapy on SARS-CoV-2 mRNA Vaccine Effectiveness in Patients with Immune-mediated Inflammatory Diseases: A Danish Nationwide Cohort Study

Rahma Elmahdi[*1,2] (0000-0002-7013-9548), Daniel Ward[*1] (0000-0003-0405-7250), Martin Thomsen Ernst[3] (0000-0001-9003-3823), Gry Juul Poulsen[1] (0000-0003-2744-2469), Jesper Hallas[3] (0000-0002-8097-8708), Anton Pottegård[3] (0000-0001-9314-5679), Tine Jess[1,4] (0000-0002-4391-7332)

SUPPLEMENTARY TABLES

Supplementary Table 1. Exposures and corresponding ATC and procedure codes for immunosuppressives

Medication	ATC code	Procedure code
**Selective immunosuppressants**		
Muromonab-CD3	L04AA02	
Antilymphocyte immunoglobulin	L04AA03	
(horse)	L04AA04	BOHJ12
Antithymocyte immunoglobulin (rabbit)	L04AA06	BOHJ22
Mycophenolic acid	L04AA10	BOHJ23
Sirolimus	L04AA13	
Leflunomide	L04AA18	BOHJ24
Everolimus	L04AA23	BOHJ26
Natalizumab	L04AA24	BOHJ18C1
Abatacept	L04AA25	BWHB84
Eculizumab	L04AA26	BOHJ19H6
Belimumab	L04AA27	BOHJ27
Fingolimod	L04AA28	
Belatacept	L04AA29	BOHJ28D
Tofacitinib	L04AA31	BOHJ28A
Teriflunomide	L04AA32	
Aprelimast	L04AA33	BOHJ19H4
Vedolizumab	L04AA34	BOHJ16A
Alemtuzumab	L04AA36	
Ocrelizumab	L04AA37	
Baricitinib	L04AA38	
Ozanimod	L04AA39	
Emapalumab	L04AA40	BWHA178
Cladribine	L04AA41	
Imlifidase	L04AA42	BWHB87
Siponimod	L04AA43	
Ravulizumab	L04AA44	
Upadacitinib		

For peer review only

**Tumour necrosis factor inhibitors**		
Etanercept	L04AB01	BOHJ18A2
Infliximab	L04AB02	BOHJ18A1
Adalimumab	L04AB04	BOHJ18A3
Certolizumab pegol	L04AB05	BOHJ18A5
Golimumab	L04AB06	BOHJ18A4
**Interleukin inhibitors**		
Basiliximab	L04AC02	
Anakinra	L04AC03	BOHJ18B1
Ustekinumab	L04AC05	BOHJ18B3
Tocilizumab	L04AC07	BOHJ18B2
Canakinumab	L04AC08	BOHJ18B4
Secukinumab	L04AC10	BOHJ18B5
Siltuximab	L04AC11	
Brodalumab	L04AC12	BOHJ18B6
Ixekizumab	L04AC13	
Sarilumab	L04AC14	BOHJ18B9
Guselkumab	L04AC16	
Tildrakizumab	L04AC17	BOHJ18B7
Risankizumab	L04AC18	BOHJ19N1
**Calcineurin inhibitors**		
Ciclosporin	L04AD01	BOHJ20
Tacrolimus	L04AD02	BOHJ21
**Other immunosuppressants**		
Azathioprine	L04AX01	BWHB83
Thalidomide	L04AX02	BWHB81
Methotrexate	L04AX03	BWHA115
Lenalidomide	L04AX04	BWHB82
Pirfenidone	L04AX05	BWHB85
Pomalidomide	L04AX06	BWHB86
Dimethyl fumarate	L04AX07	BOHJ28B
Darvadstrocel	L04AX08	
**Systemic corticosteroids**	H02AB	
**Rituximab**	L01XC02	

For peer review only

Supplementary Table 2. Minimum daily dose of corticosteroid in exposed persons

Compound	Equivalent dose
Cortisone	38mg
Hydrocortisone	30mg
Prednisone	7.5mg
Prednisolone	7.5mg
Triamcinolone	6mg
Methylprednisolone	6mg
Betamethasone	0.9mg
Dexamethasone	1.2mg

For peer review only

Supplementary Table 3. Covariates by disease cohort

IBD COHORT		
**Covariate**	**Categories/ ATC/ICD codes**	**Assessment window**
Age	-	At cohort entry
Sex	-	At cohort entry
SARS-CoV-2 tests in the previous month	-	At cohort entry
Ever previous SARS-CoV-2 positive test	-	From February 2020
IBD specific covariates		
Any IBD–related hospital admissions in the previous year	K50/K51	1 year before cohort entry
Crohn's disease	K50	At cohort entry
Ulcerative colitis	K51	At cohort entry
5-ASA/sulfasalazine	MA07EC0	1 year before matching date
Budesonide	MA07EA06	3 months before matching date
IBD related procedures<br>Operations of the small bowel and colon<br>Operations of the rectum<br>Operations of the anus and perianal tissue<br>Operational of the abdominal wall, peritoneum, mesentery and omentum<br>Lysis of adhesion in the abdominal cavity<br>Closure of intestinovaginal fistula<br>Closure of vesiculointestinal fisutal	 KJF<br>KJG<br>KJH<br>KJA<br>KJAP<br>KLEE30<br>KKCH30	Any time before cohort entry
Endoscopy of the gastrointestinal tract	KUJ	1 year before the cohort entry
Comorbidities		
Cardiovascular disease	DI1-I7	Any time before cohort entry
Pulmonary disease<br>Chronic disease of the lower airways<br>Other interstitial lung disease<br>Diseases with pus and necrosis in the lower airway<br>Interstitial lung emphysema<br>compensatory emphysema	 DJ4<br>DJ84<br>DJ85<br>DJ982<br>DJ983	Any time before cohort entry
Liver disease	DK70-K77	Any time before cohort entry
Kidney disease<br>Glomerular disease<br>Tubulointerstitial kidney disease and kidney insufficiency	 DN0<br>DN1	Any time before cohort entry
Other gastrointestinal diseases<br>Gingivitis and periodontal disease<br>Inflammation of the oral mucosa and related<br>Stomach and duodenal ulcers<br>Fissure and rifts in and around the anus<br>Abscess in and around the anus<br>Other diseases of the rectum and anus<br>Other bowel disease	 DK05<br>DK12<br>DK25-27<br>DK60<br>DK61<br>DK62<br>DK63	Any time before cohort entry

For peer review only

Skin disease		Any time before cohort entry
Bullous skin disease	DL10-14	
Dermatitis and eczema	DL20-30	
Alopecia areata	DL63	
Vitiligo	DL80	
Granulomatous disease in the skin and subcutaneous tissue	DL92	
Lupus	DL93	
Other localised connective tissue disease	DL94	
Vasculitis limited to the skin	DL95	
Musculoskeletal disease		Any time before cohort entry
Generalised connective tissue diseases	DM30-	
Diseases of the muscles	DM36	
Soft-tissue rheumatism	DM60-63	
	DM70-79	
Medications		
Cardiovascular drugs	MC01-MC10	Any time before cohort entry
Antibiotics	MJ01	Any time before cohort entry
Oral anticoagulants	MB01AA MB01AF	Any time before cohort entry
Drugs used in diabetes	MA10	Any time before cohort entry
Drugs for obstructive airway diseases	MR03	Any time before cohort entry
INFLAMMATORY ARTHROPATHIES COHORT		
Age	-	At cohort entry
Sex	-	At cohort entry
SARS-CoV-2 tests in the previous month	-	At cohort entry
Arthropathy-specific covariates		
Arthropathy-related procedures		Any time before cohort entry
Shoulder and upper arm		
Primary insertion of joint prosthesis	KNBB	
Secondary insertion of joint prosthesis	KNBC	
Operations on the joint capsule and ligaments	KNBE	
Operations of the synovia and joint surface	KNBF	
Joint resections, arthroplasties, and arthrodesis	KNBG	
Excision of bursa	KBM79	
Elbow and lower arm		
Primary insertion of joint prosthesis	KNCB	
Secondary insertion of joint prosthesis	KNCC	
Operations on the joint capsule and ligaments	KNCE	
Operations of the synovia and joint surface	KNCF	
Joint resections, arthroplasties, and arthrodesis	KNCG	
Excision of bursa	KNCM79	
Wrist and hand		
Primary insertion of joint prosthesis	KNDB	
Secondary insertion of joint prosthesis	KNDC	
Operations on the joint capsule and ligaments	KNDE	
Operations of the synovia and joint surface	KNDF	
Joint resections, arthroplasties, and arthrodesis	KNDG	
Operations on the fascia, tendon sheaths, ganglia and bursae	KNDM	

For peer review only

Hip and thigh<br>Primary insertion of joint prosthesis<br>Secondary insertion of joint prosthesis<br>Operations on the joint capsule and ligaments<br>Operations of the synovia and joint surface<br>Joint resections, arthroplasties, and arthrodesis<br>Excision of bursa in the<br>Knee and lower leg<br>Primary insertion of joint prosthesis<br>Secondary insertion of joint prosthesis<br>Operations on the joint capsule and ligaments<br>Operations of the synovia and joint surface<br>Joint resections, arthroplasties, and arthrodesis<br>Excision of bursae<br>Ankle and foot<br>Primary insertion of joint prosthesis<br>Secondary insertion of joint prosthesis<br>Operations on the joint capsule and ligaments<br>Operations of the synovia and joint surface<br>Joint resections, arthroplasties, and arthrodesis<br>Operations on the fascia, tendon sheaths, ganglia and bursae	 KNFB<br>KNFC<br>KNFE<br>KNFF<br>KNFG<br>KNFM79<br><br>KNGB<br>KNGC<br>KNGE<br>KNGF<br>KNGG<br>KNGM79<br><br>KNHB<br>KNHC<br>KNHE<br>KNHF<br>KNHG<br>KNHM	
Anti-inflammatory and anti-rheumatic drugs<br>inc. specific anti-rheumatic therapies, non-steroidals, and combination medications<br>Hydroxychloroquine	 M01<br><br>P01BA02	3 months before before cohort entry
Comorbidities		
Cardiovascular disease	DI1-I7	Any time before cohort entry
Pulmonary disease<br>Chronic disease of the lower airways<br>Other interstitial lung disease<br>Diseases with pus and necrosis in the lower airway<br>Interstitial lung emphysema<br>compensatory emphysema	 DJ4<br>DJ84<br>DJ85<br>DJ982<br>DJ983	Any time before cohort entry
Liver disease	DK70-K77	Any time before cohort entry
Kidney disease<br>Glomerular disease<br>Tubulointerstitial kidney disease and kidney insufficiency	 DN0<br>DN1	Any time before cohort entry
Other gastrointestinal diseases<br>Gingivitis and periodontal disease<br>Inflammation of the oral mucosa and related<br>Stomach and duodenal ulcers<br>Fissure and rifts in and around the anus<br>Abscess in and around the anus<br>Other diseases of the rectum and anus<br>Other bowel disease	 DK05<br>DK12<br>DK25-27<br>DK60<br>DK61<br>DK62<br>DK63	Any time before cohort entry
Skin disease<br>Bullous skin disease<br>Dermatitis and eczema<br>Alopecia areata	 DL10-14<br>DL20-30<br>DL63	Any time before cohort entry

For peer review only

Vitiligo<br>Granulomatous disease in the skin and subcutaneous tissue<br>Lupus<br>Other localised connective tissue disease<br>Vasculitis limited to the skin	DL80<br>DL92<br>DL93<br>DL94<br>DL95	
Musculoskeletal disease<br>Generalised connective tissue diseases<br>Diseases of the muscles<br>Soft-tissue rheumatism	 DM30-<br>DM36<br>DM60-63<br>DM70-79	Any time before cohort entry
Medications		
Cardiovascular drugs	MC01-MC10	Any time before cohort entry
Antibiotics	MJ01	Any time before cohort entry
Oral anticoagulants	MB01AA<br>MB01AF	Any time before cohort entry
Drugs used in diabetes	MA10	Any time before cohort entry
Drugs for obstructive airway diseases	MR03	Any time before cohort entry
PSORIASIS COHORT		
Age	-	At cohort entry
Sex	-	At cohort entry
SARS-CoV-2 tests in the previous month	-	At cohort entry
Psoriasis-specific covariates		
Procedures<br>Ultraviolet therapy: UV-A with psoralen/broad-spectrum UVB / narrow-spectrum UVB	 BNGA1/<br>BNGA2/<br>BNGA3	Any time before cohort entry
Therapeutic steroid injection in the joints and soft tissues	BLHN0	Any time before cohort entry
5-ASA/sulfasalazine	A07EC0	1 year before cohort entry
Topical corticosteroids	D07	1 year before cohort entry
Antipsoriatic medication	D05	1 year before cohort entry
Topical calcineurin inhibitors	D11AH01-02,	1 year before cohort entry
Comorbidities		
Cardiovascular disease	DI1-I7	Any time before cohort entry
Pulmonary disease<br>Chronic disease of the lower airways<br>Other interstitial lung disease<br>Diseases with pus and necrosis in the lower airway<br>Interstitial lung emphysema<br>compensatory emphysema	 DJ4<br>DJ84<br>DJ85<br>DJ982<br>DJ983	Any time before cohort entry

For peer review only

Liver disease	DK70-K77	Any time before cohort entry
Kidney disease<br>Glomerular disease<br>Tubulointerstitial kidney disease and kidney insufficiency	 DN0<br>DN1	Any time before cohort entry
Other gastrointestinal diseases<br>Gingivitis and periodontal disease<br>Inflammation of the oral mucosa and related<br>Stomach and duodenal ulcers<br>Fissure and rifts in and around the anus<br>Abscess in and around the anus<br>Other diseases of the rectum and anus<br>Other bowel disease	 DK05<br>DK12<br>DK25-27<br>DK60<br>DK61<br>DK62<br>DK63	Any time before cohort entry
Skin disease<br>Bullous skin disease<br>Dermatitis and eczema<br>Alopecia areata<br>Vitiligo<br>Granulomatous disease in the skin and subcutaneous tissue<br>Lupus<br>Other localised connective tissue disease<br>Vasculitis limited to the skin	 DL10-14<br>DL20-30<br>DL63<br>DL80<br>DL92<br>DL93<br>DL94<br>DL95	Any time before cohort entry
Musculoskeletal disease<br>Generalised connective tissue diseases<br>Diseases of the muscles<br>Soft-tissue rheumatism	 DM30-<br>DM36<br>DM60-63<br>DM70-79	Any time before cohort entry
Medications		
Cardiovascular drugs	MC01-MC10	Any time before cohort entry
Antibiotics	MJ01	Any time before cohort entry
Oral anticoagulants	MB01AA<br>MB01AF	Any time before cohort entry
Drugs used in diabetes	MA10	Any time before cohort entry
Drugs for obstructive airway diseases	MR03	Any time before cohort entry

For peer review only

Supplementary Table 4. Risk of infection, hospitalisation and death associated with immunosuppressive therapy in vaccinated patients with immune-mediated inflammatory diseases.

	**Exposed**				**Unexposed**				**Crude HR (95% CI) *****	**Weighted HR (95% CI) *****
	**Total**	**Events**	**Incidence rate (95% CI) ****	**PY**	**Total**	**Events**	**Incidence rate (95% CI) ****	**PY**		
**COVID-19 Infection***										
IBD	10 477	287	54.7 (48.7-61.4)	5 246	46 974	885	42.5 (39.8-45.4)	20 809	1.7 (1.5-1.9)	1.6 (1.4-1.9)
Arthropathy	24 255	476	40.3 (36.8-44.1)	11 822	44 650	800	37.8 (35.3-40.6)	21 140	1.3 (1.1-1.4)	1.3 (1.1-1.4)
Psoriasis	5 023	103	45.3 (37.4-55.00)	2 273	20 995	392	41.5 (37.6-45.8)	9 441	1.1 (0.9-1.4)	1.1 (0.9-1.4)
Combined cohorts	39 755	866	-	19 341	112 619	2 077	-	51 390	1.4 (1.3-1.5)	1.4 (1.2-1.5)
**COVID-19 Hospitalization***										
IBD	10 477	15	2.8 (1.7-4.7)	5 280	46 980	43	2.1 (1.5-2.8)	20 892	2.8 (1.5-5.1)	2.1 (1.0-4.1)
Arthropathy	24 256	37	3.1 (2.3-4.3)	11 873	44 650	59	2.8 (2.2-3.6)	21 215	1.3 (0.9-2.0)	1.3 (0.9-2.0)
Psoriasis	5 023	<5	-	2 284	20 999	20	2.1 (1.4-3.3)	9 479	0.5 (0.1-2.3)	0.6 (0.1-2.5)
Combined cohorts	39 756	<57	-	19 437	112 629	122	-	51 594	1.6	1.4

For peer review only - http://bmjopen.bmj.com/site/about/guidelines.xhtml

									(1.1-2.2)	(1.0-2.0)
**Death***										
IBD	10 477	<5	-	5 281	46 980	6	0.3 (0.1-0.6)	20 897	1.1 (0.1-9.7)	0.4 (0-5.6)
Arthropathy	24 256	6	0.51 (0.2-1.1)	11 875	44 650	13	0.6 (0.4-1.1)	21 222	1.0 (0.4-2.7)	1.0 (0.4-2.6)
Psoriasis	5 023	0	-	2 284	20 999	<5	-	9 480	-	-
Combined cohorts	39 756	<11	-	19 440	112 629	<24	-	51 599	1.0 (0.4-2.5)	0.9 (0.4-2.2)

Abbreviations: CI = confidence interval; HR = hazard ratio; IBD= inflammatory bowel disease; PY = person-years.

*COVID19- Infection = confirmed positive antigen or PCR test result for SARS-CoV2; COVID-19 Hospital Admission = Hospitalisation with SARS-CoV2 infection as primary diagnosis code; Death within 60 days of positive SARS-CoV2 test.

**Events/1000 PY (95% CI)

*** Sex and age adjusted crude and weighted hazard ratios

Supplementary Table 5. Risk of COVID-19 infection associated with immunosuppressive therapy in vaccinated patients with immune-mediated inflammatory diseases at 0-3 months, 3-6 months, and 6-11 months post-vaccination.

	**Exposed**				**Unexposed**				**Crude HR (95% CI)** **	**Weighted HR (95% CI)** **
	**Total**	**Events**	**Incidence rate (95% CI)** *	**PY**	**Total**	**Events**	**Incidence rate (95% CI)** *	**PY**		
**COVID-19 infection 0-3 months post-vaccination**										
IBD	10 477	57	22.2 (17.1-28.8)	2 570	46 974	196	17.0 (14.8-19.5)	11 546	1.6 (1.2-2.2)	1.5 (1.1-2.2)
Arthropathy	24 255	96	16.1 (13.2-19.6)	5 978	44 650	166	15.1 (13.0-17.6)	10 990	1.3 (1.0-1.7)	1.3 (1.0-1.7)
Psoriasis	5 023	24	19.4 (13.0-29.0)	1 236	20 995	83	16.1 (13.0-19.9)	5 163	1.3 (0.3-2.1)	1.3 (0.8-2.1)
Combined cohorts	39 755	177	-	4 403	112 619	445	-	27 699	1.4 (1.2-1.7)	1.4 (1.1-1.6)
**COVID-19 infection 3-6 months post-vaccination**										
IBD	10 010	123	45.8 (38.4-54.7)	2 683	45 202	526	56.8 (52.1-61.8)	9 266	0.9 (0.8-1.2)	1.0 (0.8-1.2)
Arthropathy	23 503	284	48.6 (43.2-54.5)	5 849	43 186	482	47.5 (43.4-51.9)	10 149	1.2 (1.1-1.4)	1.2 (1.1-1.4)
Psoriasis	4 860	63	60.7 (47.4-77.7)	1 038	20 203	233	54.5 (47.9-61.9)	4 279	1.1 (0.8-1.4)	1.0 (0.8-1.4)
Combined cohorts	38 373	470	-	19 437	108 591	1 241	-	23 694	1.1 (1.0-1.2)	1.1 (1.0-1.3)

For peer review only

For peer review only - http://bmjopen.bmj.com/site/about/guidelines.xhtml

COVID-19 infection 6-11 months post-vaccination										
IBD	6 058	107	160.6 (132.9-194.1)	666	15 742	163	107.1 (91.9-124.9)	1 522	1.3 (0.9-1.7)	1.4 (1.1-1.9)
Arthropathy	12 223	96	82.7 (67.7-101.0)	1 160	19 839	152	80.8 (68.9-94.7)	1 881	1.1 (0.8-1.4)	1.1 (0.8-1.4)
Psoriasis	1 719	16	93.8 (57.5-153.1)	170	7 444	76	109.8 (87.7-137.5)	691	0.9 (0.5-1.5)	0.9 (0.5-1.5)
Combined cohorts	20 000	219	-	1 996	43 025	391	-	4 094	1.1 (0.9-1.3)	1.11 (0.9-1.3)

Abbreviations: CI = confidence interval; HR = hazard ratio; IBD= inflammatory bowel disease; PY = person-years.

*Events/1000 PY

**Sex and age adjusted crude and weighted hazard ratios

For peer review only

Supplementary Table 6. Risk of COVID infection associated with immunosuppressive therapy in vaccinated patients with immune-mediated inflammatory diseases by calendar period (1st January-31st November 2021)

	Exposed		Unexposed		Hazard Ratio (HR)	
	**Total**	**Events**	**Total**	**Events**	**Crude HR (95% CI)**	**Weighted HR (95% CI)** **
***COVID-19 infection from 1st January to 30th April**						
IBD	5 745	n<5	10 306	7	0.67 (0.17-2.61)	0.39 (0.09-1.71)
Arthropathy	9 646	9	13 044	17	0.75 (0.33-1.69)	0.78 (0.35-1.71)
Psoriasis	1 298	n<5	4 512	6	2.00 (0.50-8.02)	1.98 (0.48-8.19)
Combined IMID	16 689	<19	27 862	30	0.87 (0.48-1.59)	0.83 (0.44-1.54)
**COVID-19 infection 1st May to 31st August**						
IBD	10 141	56	45 486	121	1.61 (1.17-2.22)	1.19 (0.82-1.73)
Arthropathy	23 885	74	43 457	116	1.08 (0.81-1.45)	1.07 (0.79-1.43)
Psoriasis	4 912	12	20 337	53	0.88 (0.47-1.65)	0.84 (0.44-1.58)
Combined IMID	38 938	142	109 280	290	1.24 (1.01-1.52)	1.08 (0.87-1.34)
**COVID-19 infection 1st September to 30st November**						
IBD	1 0349	228	46 590	757	1.69 (1.45-1.97)	1.74 (1.48-2.04)
Arthropathy	23 898	393	44 079	667	1.34 (1.18-1.51)	1.33 (1.17-1.51)
Psoriasis	4 974	88	20 803	333	1.17 (0.93-1.48)	1.13 (0.89-1.44)
Combined IMID	39 221	709	111 472	1757	1.42 (1.30-1.55)	1.42 (1.29-1.56)

Abbreviations: CI = confidence interval; HR = hazard ratio; IBD= inflammatory bowel disease.

*COVID19- Infection = confirmed positive antigen or PCR test result for SARS-CoV2

** Sex and age adjusted crude and weighted hazard ratios

For peer review only

Supplementary Table 7. Risk of infection, hospitalisation and death associated with immunosuppressive therapy in vaccinated patients with immune-mediated inflammatory diseases by immunosuppressive drug class.

	**Exposed**				**Unexposed**				**Crude HR (95% CI) *****	**Weighted HR (95% CI) *****
	**Total**	**Events**	**PY**	**Incidence rate (95% CI) ****	**Total**	**Events**	**PY**	**Incidence rate (95% CI) ****		
**COVID-19 Infection***										
Selective Immunosuppressants	3 384	68	1 846	39.4 (31.0-49.9)	151 936	2 079	51 334	40.5 (38.8-42.3)	1.3 (1.0-1.7)	1.3 (1.0-1.6)
Tumour Necrosis Factor Inhibitors	14 277	418	6 908	62.5 (56.7-68.7)	140 797	2 070	51 355	40.3 (38.6-42.1)	1.8 (1.6-2.0)	1.8 (1.6-2.0)
Interleukin Inhibitors	2 781	52	1 385	40.1 (30.6-52.7)	152 584	2 082	51 352	40.5 (38.8-42.3)	1.1 (0.8-1.4)	1.0 (0.8-1.4)
Calcineurin Inhibitors	154	n<5	129	-	155 309	2 073	51 298	40.4 (38.7-42.2)	1.3 (0.3-5.2)	0.5 (0.2-1.5)
Systemic Corticosteroids	5 504	97	2 991	34.6 (28.4-42.3)	149 667	2 079	51 503	40.4 (38.7-42.1)	1.3 (1.0-1.6)	1.2 (1.0-1.5)
Rituximab and Other Immunosuppressants	21 038	426	10 703	41.2 (37.5-45.3)	112 593	2 075	51 409	40.4 (38.7-42.1)	1.3 (1.2-1.4)	1.3 (1.1-1.4)
**COVID-19 Hospitalization***										
Selective Immunosuppressants	3 384	7	1 735	4.0 (1.9-8.5)	112 479	122	51 531	2.4 (2.0-2.8)	3.0 (1.4-6.5)	2.2 (1.0-5.2)
Tumour Necrosis Factor Inhibitors	14 277	16	6 740	2.4 (1.5-3.9)	112 506	116	51 551	2.3 (1.9-2.1)	2.0 (1.2-3.3)	1.8 (1.0-3.3)

For peer review only

Interleukin Inhibitors	2 781	0	1 302	-	112 445	125	51 549	2.4 (2.0-2.9)	-	-
Calcineurin Inhibitors	154	0	83	-	112 404	122	51 493	2.4 (2.0-2.8)	-	-
Systemic Corticosteroids	5 504	18	2 811	6.4 (4.0-10.2)	112 587	122	51 699	2.4 (2.0-2.8)	2.4 (1.4-3.9)	1.8 (1.0-3.0)
Rituximab and Other Immunosuppressants	21 038	25	10 387	2.4 (1.6-3.6)	112 593	121	51 604	2.3 (2.0-2.8)	1.2 (0.5-1.0)	1.1 (0.7-1.8)
**Death***										
Selective Immunosuppressants	3 384	0	1 736	-	112479	23	51 545	0.5 (0.3-0.7)	-	-
Tumour Necrosis Factor Inhibitors	14 277	0	6 741	-	112506	19	5 1564	0.4 (0.2-0.6)	-	-
Interleukin Inhibitors	2 781	0	1 302	-	112 445	24	51 564	0.5 (0.3-0.7)	-	-
Calcineurin Inhibitors	154	0	83	-	112 404	24	51 507	0.5 (0.3-0.7)	-	-
Systemic Corticosteroids	5 504	6	2 812	2.1 (2.0-4.8)	112 587	23	51 713	0.4 (0.3-0.7)	4.0 (1.6-9.9)	2.3 (0.9-6.2)
Rituximab and Other Immunosuppressants	21 038	n<5	10 388	-	112 593	22	51 619	0.4 (0.3-0.7)	0.5 (0.1-2.3)	0.5 (0.1-2.4)

For peer review only

Abbreviations: CI = confidence interval; HR = hazard ratio; IBD= inflammatory bowel disease; PY = person-years.

Immunosuppressive Drug Class: Selective Immunosuppressants, Tumour Necrosis Factor Inhibitors, Interleukin Inhibitors, Calcineurin Inhibitors, Systemic Corticosteroids, and Other Immunosuppressants including Rituximab (See Supplementary for complete ATC codes)

*COVID19- Infection = confirmed positive antigen or PCR test result for SARS-CoV2; COVID-19 Hospital Admission = Hospitalisation with SARS-CoV2 infection as primary diagnosis code; Death within 60 days of positive SARS-CoV2 test.

**Events/1000 PY

*** Sex and age adjusted crude and weighted hazard ratios

For peer review only

SUPPLEMNETRAY FIGURES

Supplementary Figure 1. Kaplan-Meier plots for probability of infection with COVID-19 following second mRNA vaccination in immunosuppressive exposed compared to propensity score matched immunosuppressive unexposed a. IBD patients b. Arthropathy patients c. Psoriasis patients over the calendar year from January 1st to 31st November 2021. SMR weighted = standardized mortality ratio weighted.

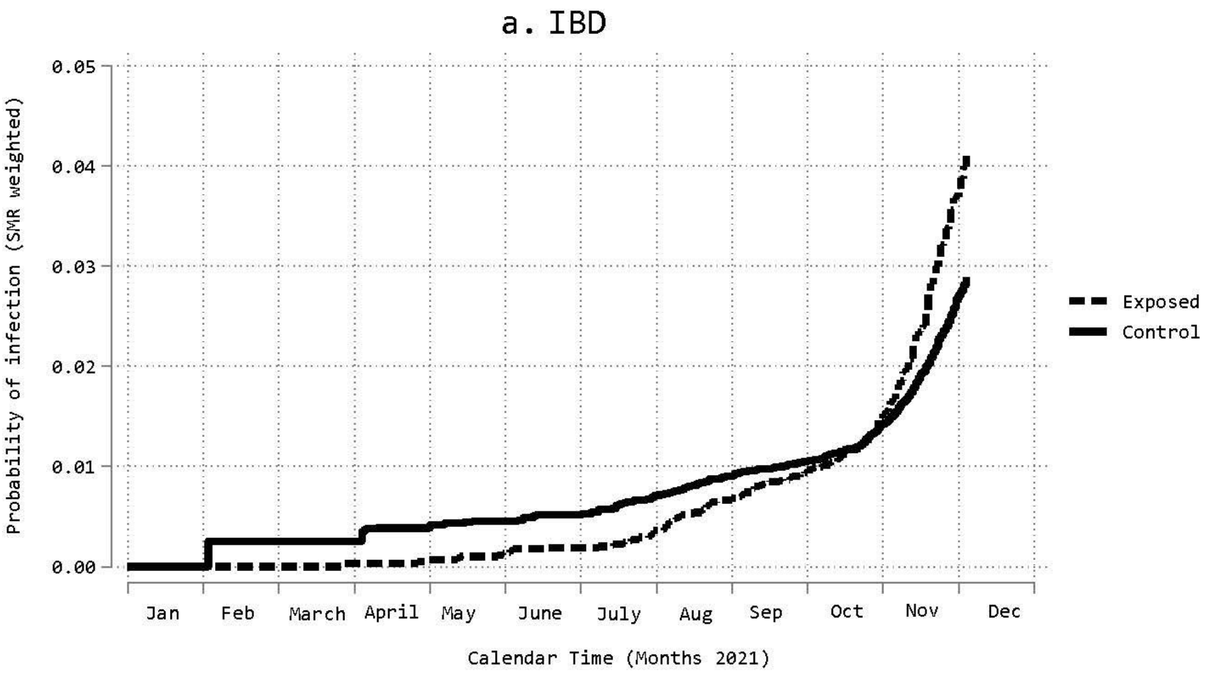

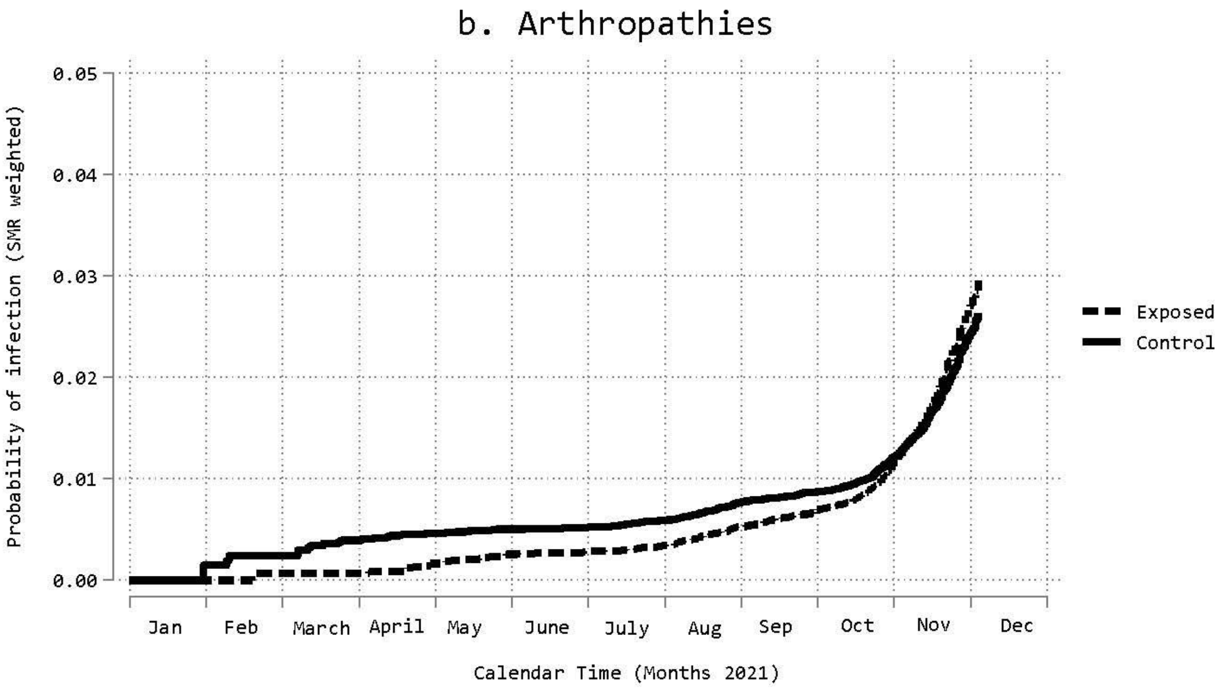

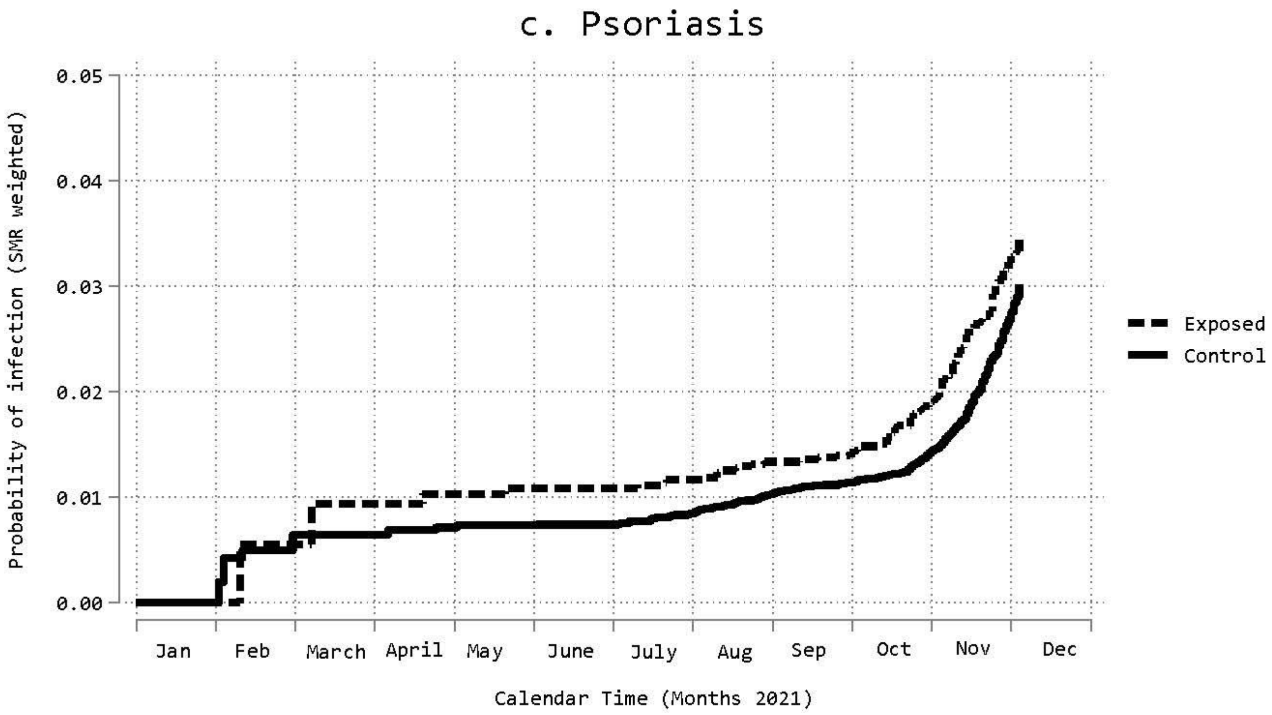

For peer review only

STROBE Statement—Checklist of items that should be included in reports of ***cohort studies***

The Impact of Immunosuppressive Therapy on SARS-CoV-2 mRNA Vaccine Effectiveness in Patients with Immune-mediated Inflammatory Diseases: A Nationwide Danish Cohort Study

	Item No	Recommendation	Page No
**Title and abstract**	1	(*a*) Indicate the study's design with a commonly used term in the title or the abstract	3
		(*b*) Provide in the abstract an informative and balanced summary of what was done and what was found	3
**Introduction**			
Background/rationale	2	Explain the scientific background and rationale for the investigation being reported	5
Objectives	3	State specific objectives, including any prespecified hypotheses	5, 6
**Methods**			
Study design	4	Present key elements of study design early in the paper	6-8
Setting	5	Describe the setting, locations, and relevant dates, including periods of recruitment, exposure, follow-up, and data collection	6, 7 & Fig.1
6-10Participants	6	(*a*) Give the eligibility criteria, and the sources and methods of selection of participants. Describe methods of follow-up	6-8 &
		(*b*) For matched studies, give matching criteria and number of exposed and unexposed	Fig.1
Variables	7	Clearly define all outcomes, exposures, predictors, potential confounders, and effect modifiers. Give diagnostic criteria, if applicable	7,8
Data sources/ measurement	8*	For each variable of interest, give sources of data and details of methods of assessment (measurement). Describe comparability of assessment methods if there is more than one group	7,8
Bias	9	Describe any efforts to address potential sources of bias	6-9
Study size	10	Explain how the study size was arrived at	Fig. 1
Quantitative variables	11	Explain how quantitative variables were handled in the analyses. If applicable, describe which groupings were chosen and why	7-9
Statistical methods	12	(*a*) Describe all statistical methods, including those used to control for confounding	7-9
		(*b*) Describe any methods used to examine subgroups and interactions	
		(*c*) Explain how missing data were addressed	
		(*d*) If applicable, explain how loss to follow-up was addressed	
		(*e*) Describe any sensitivity analyses	
**Results**			
Participants	13*	(a) Report numbers of individuals at each stage of study—eg numbers potentially eligible, examined for eligibility, confirmed eligible, included in the study, completing follow-up, and analysed	9,10 & Table 1
		(b) Give reasons for non-participation at each stage	
		(c) Consider use of a flow diagram	
Descriptive data	14*	(a) Give characteristics of study participants (eg demographic, clinical, social) and information on exposures and potential confounders	9,10

For peer review only - http://bmjopen.bmj.com/site/about/guidelines.xhtml

		(b) Indicate number of participants with missing data for each variable of interest<br>(c) Summarise follow-up time (eg, average and total amount)	
Outcome data	15*	Report numbers of outcome events or summary measures over time	9 Fig.2-4
Main results	16	(*a*) Give unadjusted estimates and, if applicable, confounder-adjusted estimates and their precision (eg, 95% confidence interval). Make clear which confounders were adjusted for and why they were included<br>(*b*) Report category boundaries when continuous variables were categorized<br>(*c*) If relevant, consider translating estimates of relative risk into absolute risk for a meaningful period	9,10 & Fig. 2-4
Other analyses	17	Report other analyses done—e.g. analyses of subgroups and interactions, and sensitivity analyses	10 Fig.4
**Discussion**			
Key results	18	Summarise key results with reference to study objectives	10
Limitations	19	Discuss limitations of the study, taking into account sources of potential bias or imprecision. Discuss both direction and magnitude of any potential bias	13
Interpretation	20	Give a cautious overall interpretation of results considering objectives, limitations, multiplicity of analyses, results from similar studies, and other relevant evidence	13
Generalisability	21	Discuss the generalisability (external validity) of the study results	11,12
**Other information**			
Funding	22	Give the source of funding and the role of the funders for the present study and, if applicable, for the original study on which the present article is based	Abstract & 14

*Give information separately for exposed and unexposed groups.

Note: An Explanation and Elaboration article discusses each checklist item and gives methodological background and published examples of transparent reporting. The STROBE checklist is best used in conjunction with this article (freely available on the Web sites of PLoS Medicine at http://www.plosmedicine.org/, Annals of Internal Medicine at http://www.annals.org/, and Epidemiology at http://www.epidem.com/). Information on the STROBE Initiative is available at http://www.strobe-statement.org.

For peer review only

For peer review only - http://bmjopen.bmj.com/site/about/guidelines.xhtml
